# Microbiome signature of Parkinson’s disease in healthy and genetically at-risk individuals

**DOI:** 10.1038/s41591-026-04318-5

**Published:** 2026-04-20

**Authors:** Elisa Menozzi, Yani Ren, Mallia Geiger, Jane Macnaughtan, Micol Avenali, Marco Toffoli, Marine Gilles, Rosaria Calabrese, Pierfrancesco Mitrotti, Luca Gallo, Alexandre Famechon, Sara Lucas Del Pozo, Roxana Mezabrovschi, Sofia Koletsi, Nadine Loefflad, Selen Yalkic, Naomi Limbachiya, Frederick Clasen, Suleyman Yildirim, Saeed Shoaie, Hervé Blottière, Christian Morabito, Aymeric David, Benoit Quinquis, Nicolas Pons, Emmanuelle Le Chatelier, Franco Valzania, Francesco Cavallieri, Valentina Fioravanti, Giulia Toschi, Fabio Blandini, Mathieu Almeida, Stanislav Dusko Ehrlich, Victoria Meslier, Anthony H. V. Schapira

**Affiliations:** 1 Department of Clinical and Movement Neurosciences, UCL Queen Square Institute of Neurology, London, UK; 2 Aligning Science Across Parkinson’s (ASAP) Collaborative Research Network, Chevy Chase, MD USA; 3 Université Paris-Saclay, INRAE, MetaGenoPolis, Jouy-en-Josas, France; 4 Liver Failure Group, Institute for Liver and Digestive Health, University College London, London, UK; 5 Department of Brain and Behavioral Sciences, University of Pavia, Pavia, Italy; 6 IRCCS Mondino Foundation, Pavia, Italy; 7 Centre for Host-Microbiome Interactions, Faculty of Dentistry, Oral & Craniofacial Sciences, King’s College London, London, UK; 8 Department of Medical Microbiology, Istanbul Medipol University International School of Medicine, Istanbul, Turkey; 9 Regenerative and Restorative Medicine Research Center (REMER), Research Institute for Health Sciences and Technologies (SABITA), Istanbul Medipol University, Istanbul, Turkey; 10 Nantes Université, INRAE PhAN, Nantes, France; 11 Neurology Unit, Azienda Unità Sanitaria Locale-IRCCS di Reggio Emilia, Reggio Emilia, Italy; 12 IRCCS Ca’ Granda Foundation, Ospedale Maggiore Policlinico, Milan, Italy

**Keywords:** Parkinson's disease, Predictive markers, Microbiome

## Abstract

Parkinson’s disease (PD) is a major cause of disability. *GBA1* variants are the most common genetic risk factor for PD and increase the risk up to 30-fold. Why only approximately 20% of *GBA1* variant carriers develop PD remains unknown. Here, by combining clinical and fecal metagenomics data from 271 patients with PD, from 43 carriers of *GBA1* variants not manifesting PD symptoms (GBA-NMC) and from 150 healthy controls, and using an innovative microbiome analysis, combining differential abundance of species and coherence of differential abundance variation between the groups as assessed by Cliff’s delta (*δ*), we show that the composition of a large component of the gut microbiome (approximately 25%) in GBA-NMC is intermediate between healthy controls and patients with PD. This component is strongly correlated with disease progression in patients and prodromal symptoms suggestive of future development of PD in both GBA-NMC and healthy individuals. We found microbiome alterations similar to those described here in three independent cohorts from the United States, Korea and Turkey, totaling 638 patients with PD and 319 healthy controls, and we conclude that gut microbiome alterations can identify both genetically and non-genetically at-risk individuals in the general population who may be progressing toward PD, thus serving as an early marker of disease development in the premanifest phase.

## Abstract (web summary)

Microbiome analysis suggests that gut microbial changes in Parkinson’s disease evolve progressively from healthy individuals to genetically at-risk individuals to clinically affected patients, with the degree of dysbiosis correlating with disease progression.

## Main

Among neurodegenerative disorders, PD is the fastest growing in prevalence, disability and deaths^1^. The economic burden associated with direct medical costs, disability income, costs for paid care and social productivity loss in the United States is estimated to increase from $52 billion in 2017 to $79 billion in 2037 (ref. ^2^). PD is characterized pathologically by loss of dopaminergic neurons of the substantia nigra pars compacta and accumulation of aggregated α-synuclein in the brainstem and several cortical regions^3^. When motor symptoms appear and a clinical diagnosis is possible, the extent of dopaminergic loss is already greater than 50%^4^. Symptomatic treatment is based on dopamine replacement by administration of its precursor levodopa^3^. Slowing, halting or preventing the neurodegenerative process before it translates into clinically evident or disabling symptoms requires early detection of those individuals at risk of, or progressing toward, disease.

The background of PD is likely multifactorial, resulting from both genetic factors, including single gene, risk gene and polygenic contributions, and potentially non-genetic factors^5^. Genetic variants in the *GBA1* gene, encoding the lysosomal enzyme glucocerebrosidase, are found in approximately 15% of PD cases^6^, representing the most common genetic risk factor for PD and one of the most appealing targets for new drug development^6,7^. However, only a proportion of individuals carrying *GBA1* variants will develop PD over their lifetime, with penetrance being estimated at 10% at 60 years up to 19% at 80 years^8^. To date, there are still limited clinical, imaging or biochemical markers that can stratify *GBA1* variant carriers for their future risk of developing PD^9^.

Evidence supports a role of the microbiota−gut−brain axis in the pathogenesis of PD. Although motor symptoms remain the core criteria to diagnose PD, non-motor symptoms, such as rapid eye movement (REM) sleep behavior disorder (RBD), hyposmia and autonomic dysfunction, including constipation, can predate the onset of motor symptoms by many years in some patients and, thus, are referred to as prodromal symptoms^5^. Based on presence or absence of prodromal RBD, patients with PD can be classified as body-first or brain-first, with the former predicted to manifest initial signs of neurodegeneration and accumulation of α-synuclein in the autonomic and enteric nervous system with subsequent spread to the central nervous system, whereas the latter is predicted to manifest initial pathology in the brain^10^. Alterations in gut microbiome composition have been detected in individuals with overt PD^11,12^, especially in body-first PD^13^. Moreover, gut dysbiosis can precipitate PD in animal models^14^, and exposure to microbial components can induce α-synuclein accumulation in gut enteroendocrine cells^15^.

By comparing gut microbiomes of a cohort of patients with PD (*n* = 271), disease-free *GBA1* variant carriers (*n* = 43) and healthy controls (*n* = 150), we found large-scale PD-specific alterations in individuals of the last two groups, which are associated with a PD prodromal clinical profile. We suggest that alterations in gut microbiome composition might help explain the incomplete penetrance of PD in *GBA1* variant carriers and identify those at highest risk of conversion to PD. We further suggest that similar changes in healthy individuals without known genetic risk may also help identify those predisposed toward PD development and be used as a marker of early disease development in the premanifest phase.

## Results

### Study participants

A total of 540 participants were included in the full analysis set for clinical data. Participants’ characteristics are shown in Table 1. Participants with PD (*n* = 314) included both carriers (*n* = 128) and non-carriers (*n* = 186) of *GBA1* variants (Appendix A). The individuals without PD included healthy controls non-carriers of *GBA1* variants (*n* = 175, HC) and non-manifesting *GBA1* variant carriers (*n* = 51, GBA-NMC). When age and sex of GBA-NMC were compared to the other groups, no differences were detected. Participants with PD were significantly older than HC (*P*= 0.006) and more frequently males (*P* = 0.028). More than half of the HC participants were partners of people with PD, to mitigate the effect of diet or other lifestyle-associated variables on gut microbiome composition.

**Table 1:** Overview of study cohort

	HC (*n* = 175)	GBA-NMC (*n* = 51)	PD (*n* = 314)	*P* (HC vs GBA-NMC)	*P* (GBA-NMC vs PD)
Demographics					
GBR cohort (*n*)	87	40	155	−	−
ITA cohort (*n*)	88	11	159	−	−
Age	60 ± 10	61 ± 11	63 ± 9	NS	NS
Sex (% F, *n*)	58% (102)	57% (29)	44% (139)	NS	NS
Positive PD family history (%, *n*)	32% (56)	51% (26)	29% (90)	0.0206	0.0026
Education (years)	14.7 ± 4.3	15.8 ± 4.0	14 ± 4.3	NS	0.0169
Participation with partner (%, *n*)	52% (91)	27% (14)	26% (83)	0.0035	NS
Participation with family member (%, *n*)	12% (21)	41% (21)	10% (32)	<0.001	<0.001
BMI (kg m^−^^2^)	25.6 ± 4.4	26.1 ± 3.9	25.7 ± 4.9	NS	NS
Disease-associated features					
PD age at onset	−	−	57 ± 10	−	−
PD duration	−	−	6.4 ± 5	−	−
Drug naive (%, *n*)	−	−	11% (36)	−	−
LEDD (mg)	−	−	590.7 ± 452.8	−	−
DBS (%, *n*)	−		13% (40)	−	−
Apomorphine pump infusion (%, *n*)	−	−	0.3% (1)	−	−
LCIG (%, *n*)	−	−	0.3% (1)	−	−
Clinical features					
MDS-UPDRS part I	3.4 ± 3.9	5.2 ± 4.7	10.3 ± 6.6	**0.0036**	**<0.0001**
MDS-UPDRS part II	0.4 ± 1	1.2 ± 1.8	10.8 ± 6.6	**0.0008**	**<0.0001**
MDS-UPDRS part III	2.2 ± 3.3	3.5 ± 3.8	27.4 ± 12.9	0.0153	**<0.0001**
MDS-UPDRS part IV	−	−	3.1 ± 3.5	−	−
MDS-UPDRS total	6 ± 6.4	9.9 ± 7.5	51.3 ± 22.1	**0.0002**	**<0.0001**
H&Y stage (median)	0	0	2	NS	**<0.0001**
SCOPA-AUT total	6.5 ± 4.5	7.9 ± 5.4	13.9 ± 7.5	NS	**<0.0001**
SCOPA Gastrointestinal	1.1 ± 1.3	1.2 ± 1.3	3.9 ± 2.9	NS	**<0.0001**
SCOPA Urinary	2.8 ± 2.3	3.7 ± 2.7	5 ± 3.0	0.0198	**0.0019**
SCOPA Cardiovascular	0.2 ± 0.6	0.4 ± 0.7	0.8 ± 1.1	NS	**0.0071**
SCOPA Thermoregulatory	1.2 ± 1.4	1.3 ± 1.7	2.3 ± 2.2	NS	**0.0009**
SCOPA Pupillomotor	0.4 ± 0.7	0.5 ± 0.7	0.5 ± 0.8	NS	NS
SCOPA Sexual	0.8 ± 1.2	0.7 ± 1.2	1.5 ± 1.8	NS	**0.0053**
WCSS	2.5 ± 2.5	3.2 ± 3.2	5.9 ± 4.5	NS	**<0.0001**
RBDSQ (score)*	1.6 ± 2	2.3 ± 2.3	5.3 ± 3.6	0.0407	<0.0001
RBDSQ above cutoff (%, *n*)	10% (17)	18% (9)	39% (122)	NS	**0.0042**
UPSIT	29.6 ± 4.5	29.7 ± 4.8	18.3 ± 6.3	NS	**<0.0001**
HADS anxiety	4 ± 3.3	4.1 ± 3.5	5.7 ± 3.8	NS	**0.0016**
HADS depression	2.7 ± 2.9	2.7 ± 2.7	5.4 ± 3.7	NS	**<0.0001**
BDI	5.2 ± 5.4	6 ± 6.7	9.8 ± 6.7	NS	**<0.0001**
MOCA (score)^a^	27.3 ± 2.1	27 ± 2.9	25.7 ± 3.8	NS	NS
MOCA below cutoff (%, *n*)	18% (32)	29% (15)	37% (117)	0.0197	NS

BDI, Beck Depression Inventory; DBS, deep brain stimulation; F, female; HADS, Hospital Anxiety and Depression Scale; H&Y, Hoehn & Yahr; LCIG, levodopa-carbidopa intestinal gel; GBR, Great Britain; ITA, Italy; SCOPA-AUT, Scales for Outcomes in Parkinsonʼs Disease-Autonomic Dysfunction. *P* values (two-sided *t*-test or non-parametric Wilcoxon rank-sum test) are reported as numerical values or as NS (not significant). *P* values related to clinical features that resisted adjustment for multiple comparisons are indicated in bold.

^a^ For RBDSQ and MOCA, total scores are reported in Table 1 for completeness, and *P* values of total scores’ comparisons are also reported, although they have not been included in the adjustment for multiple testing (as *P* values from binary regression models for RBDSQ and MOCA have been included).

### Clinical profile of GBA-NMC

To identify possible clinical elements that could stratify GBA-NMC for their risk of developing PD, we compared the severity of motor and non-motor symptoms between HC and GBA-NMC using a wide range of clinical scales and questionnaires (Table 1). We found worse motor symptoms in the GBA-NMC group, either subjectively reported (Movement Disorder Society Unified Parkinson’s Disease Rating Scale (MDS-UPDRS) part II, *P* = 0.0008, *q* = 0.0076) or objectively assessed (MDS-UPDRS part III, *P* = 0.0153, *q* = 0.0627), with the former remaining significant even after adjustment for multiple testing. In terms of non-motor symptoms, no differences in constipation or global autonomic function were detected; however, GBA-NMC showed significantly higher scores in the MDS-UPDRS part I (*P* = 0.0036, *q* = 0.0228) and more severe urinary symptoms (*P* = 0.0198) and cognitive impairment (*β* = 0.92, *P* = 0.0197, odds ratio = 2.5, 95% confidence interval: 1.2−5.5), although these did not survive to multiple testing adjustment (both *q* = 0.0627). No differences in depression, anxiety or olfactory function were observed. Within the GBA-NMC, 10 individuals reached the threshold for estimated probability according to the MDS prodromal criteria calculated based on available information (listed in Methods)^16^.

Overall, our clinical data suggest that, in our GBA-NMC cohort, there may be a group of individuals who exhibit some prodromal symptoms (for example, subthreshold parkinsonism and some dysautonomia) and, thus, might be in their prodromal phase of PD. Additional biological markers indicating PD proximity are needed to better refine individualized risk of PD.

### Significant microbiome alterations in PD

Microbiome profiles were successfully generated for 464 individuals (150 HC, 43 GBA-NMC and 271 PD). We first compared microbiomes of patients with PD, carriers (*n* = 109) or non-carriers (*n* = 162) of *GBA1* variants, and found 44 Metagenomic Species Pan-genomes (MSPs) out of 627 with prevalence of at least 10% in our cohort that were different in abundance at *P* < 0.05 by two-sided Wilcoxon rank-sum test. However, none remained significantly different after Benjamini−Hochberg correction for multiple testing at *q* < 0.05 (or even *q* < 0.6). By contrast, the comparison of either group with HC (*n* = 150) revealed numerous MSPs significant at *q* < 0.05 (50 and 83 for carrier and non-carrier groups, respectively; the difference is likely due to the higher statistical power for the more numerous non-carrier group). *β* diversity analysis of Bray−Curtis distances by PERMANOVA confirmed that there was no significant microbiome difference between carrier and non-carrier groups (adonis *P* = 0.46), whereas the difference between HC and either group was very significant (*P* = 0.001). We conclude that, in overt PD, *GBA1* genetic status impacts the microbiome composition much less than the disease itself.

**Extended Data Fig. 1:**
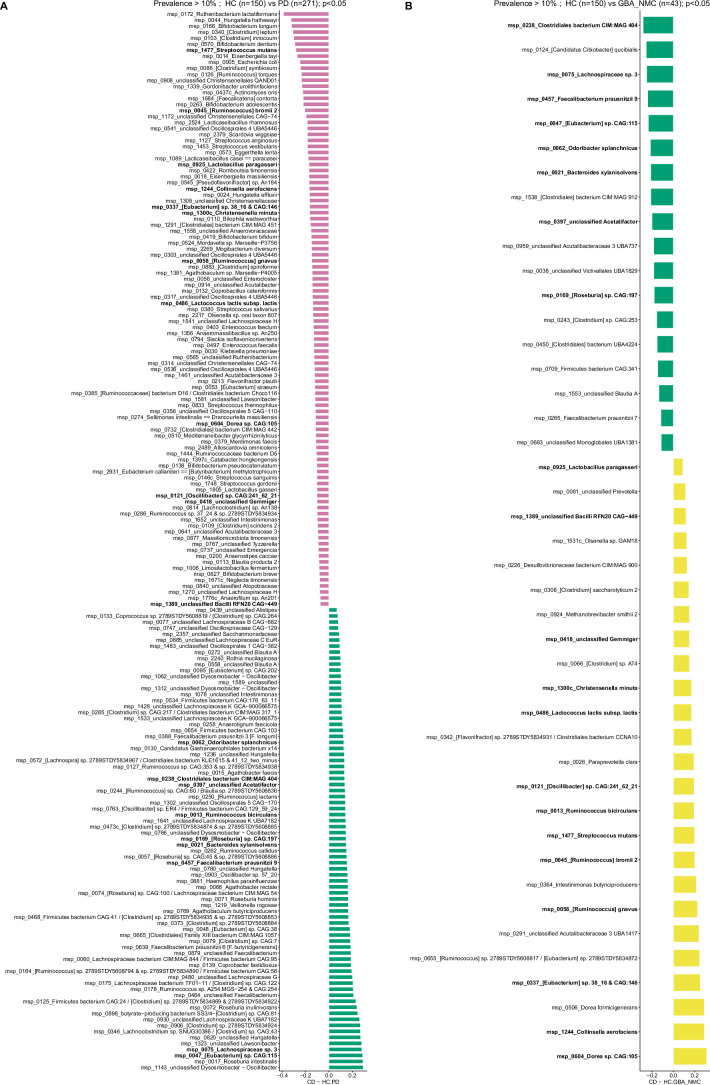
Gut microbiome variations in PD and GBA-NMC compared to HC. **a**. Barplot representing the 176 differentially abundant species observed in PD (n = 271) compared to HC (n = 150) individuals (two-sided Wilcoxon p < 0.05). Species enriched and depleted in PD are shown in mauve and green, respectively. **b**. Barplot representing the 43 differentially abundant species observed in GBA-NMC (n = 43) compared to HC (n = 150) individuals (two-sided Wilcoxon p < 0.05). Species enriched and depleted in GBA-NMC are shown in yellow and green, respectively. The 21 significant species commonly altered between HC-PD and HC-GBA-NMC are highlighted in bold. All species except one (*msp_0013 Ruminococcus bicirculans*) are coherently altered between HC-PD and HC-GBA-NMC. Wilcoxon p values are displayed in Supplementary Tables 3 and 4.

We, therefore, pooled all patients with PD regardless of their genetic status (*n* = 271) to identify the PD gut microbiome signature at maximal statistical power and compared them with HC (*n* = 150). A total of 176 species were differentially abundant at *P* < 0.05 (Supplementary Table 1 and Extended Data Fig. 1a; 103 at *q* < 0.05). In PD, Actinobacteriota were enriched at phylum level and *Bifidobacteriaceae* at family level (Supplementary Table 2), as previously reported^12,17,18^. By contrast, species enriched in HC belonged to *Lachnospiraceae C* and *Ruminococcaceae*. These families include butyrate producers such as *Roseburia* and *Dysosmobacter*, which may be antiinflammatory. *Bifidobacterium* and *Faecalibacterium* genera were enriched in PD and HC, respectively. Enrichment of *Bifidobacterium* and depletion of butyrate producers such as *Faecalibacterium* in patients with PD were previously reported^19,20^. Species that showed the greatest increase in PD included *Streptococcus mutans*, *Bifidobacterium longum*, *Bifidobacterium dentium* and *Lactobacillus paragasseri*, whereas species that showed the greatest depletion in PD included *Roseburia intestinalis*, *Roseburia inulinivorans* and an unclassified *Faecalibacterium*. Further analyses were based on the 176 species differentially abundant at *P* < 0.05.

### An intermediate and consistent microbiome alteration in GBA-NMC

**Fig. 1:**
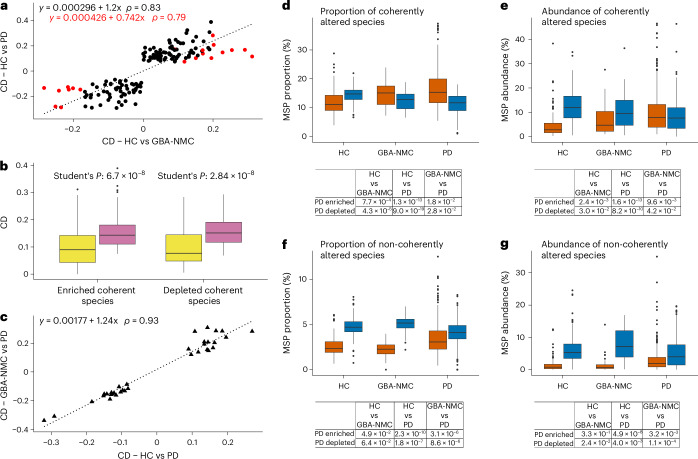
Variation of the coherent and non-coherent gut microbiome species in HC individuals (*n* = 150), GBA-NMC participants (*n* = 43) and patients with PD (*n* = 271). **a**, Correlations of Cliff’s *δ* (CD) of 142 coherent species found by comparing HC individuals with GBA-NMC or patients with PD. Negative CD values are species found enriched in HC, and positive CD values are species found enriched in both GBA-NMC and PD. Red dots highlight the 20 significant and coherently altered species (two-sided Wilcoxon *P* < 0.05) overlapping between HC−PD and HC−GBA-NMC comparisons. *ρ* denotes Spearmanʼs correlation coefficient when considering the 142 or 20 species. **b**, Average CD of coherently enriched (*n* = 81) or depleted (*n* = 61) species in GBA-NMC (yellow) and PD (mauve) relative to HC. **c**, Correlation of CD values of 34 species altered significantly (two-sided Wilcoxon *P* < 0.05) and non-coherently in the comparison of HC and patients with PD and in the comparison of PD and GBA-NMC. *ρ* denotes Spearmanʼs correlation coefficient. **d**,**e**, Abundance and proportion of coherent altered species in HC, GBA-NMC and PD and corresponding *P* values (determined by one-sided Studentʼs *t*-test for different comparisons); orange and blue refer to coherent enriched and depleted species, respectively. **f**,**g**, Abundance and proportion of non-coherent altered species in the three study groups and corresponding *P* values (determined by one-sided Studentʼs *t*-test); orange and blue refer to non-coherent enriched and depleted species, respectively. In the box plots, central line denotes the median, boxes the central quartiles, whiskers the extreme quartiles and dots the outliers.

We then investigated whether some of the PD-related gut microbiome alterations were detectable in the GBA-NMC group (*n* = 43) relative to HC (*n* = 150) by comparing species abundance in the two groups. A total of 43 species were significantly different at *P* < 0.05 (Supplementary Table 3 and Extended Data Fig. 1b), fewer than in the comparison of HC with PD, possibly, at least in part, because of the loss of statistical power due to the low number of GBA-NMC. Of these, 21 were common with the 176 species significantly altered in patients with PD compared to HC, and all but one were enriched or depleted in GBA-NMC individuals coherently with enrichment or depletion in patients with PD, using the direction of Cliff’s *δ* as a guide (positive or negative, respectively), which is unlikely to happen by chance (*χ*^2^ test, *P* = 5.7 × 10^−3^). The effect sizes observed for the common MSPs in the comparisons between GBA-NMC versus HC and PD versus HC were highly correlated (red dots, Fig. 1a).

Notably, using the direction of Cliff’s *δ*, examination of the coherence of variation of the 176 MSPs significantly different between HC and PD individuals revealed that 142 were coherently altered in GBA-NMC and PD relative to HC (Supplementary Table 4), which is very unlikely to happen by chance (*χ*^2^ test, *P*= 3.9 × 10^−16^). Of these, 81 MSPs were enriched and 61 were depleted in PD; we term these coherent enriched and coherent depleted species, respectively. Effect sizes of these species in GBA-NMC and PD relative to HC were highly correlated (Fig. 1a) and were, on average, significantly lower in GBA-NMC than in PD (Fig. 1b), suggesting that the GBA-NMC microbiome may be in an intermediate state between that of HC and PD. The remaining 34 species were found not to vary coherently in GBA-NMC and PD relative to HC and are hereafter termed non-coherent species; 18 were non-coherent enriched and 16 were non-coherent depleted in PD (Supplementary Table 4). Their Cliff’s *δ* values in GBA-NMC and HC relative to PD were highly correlated (Fig. 1c). Among the coherent species, we observed enrichment of oral residents (*S. mutans* and *L. paragasseri*) and proinflammatory *Ruminococcus gnavus* and the depletion of butyrate producers (*Roseburia* or *Faecalibacterium prausnitzii*). By contrast, non-coherent enriched species included *Bifidobacteria*, one of the characteristic alterations of the PD gut microbiome^17^, suggesting that their enrichment may take place at the clinical onset of the disease or develop during its course.

The species coherently altered in GBA-NMC and PD relative to HC represented a similar fraction (slightly over 25%) of the microbiome in all study groups, but the abundance and proportion of PD-enriched species significantly increased from HC over GBA-NMC to PD, whereas the abundance and proportion of PD-depleted species significantly decreased (Fig. 1d,e). The non-coherent species represented approximately 7–9% of microbiome abundance and 7% of species proportion. This part of the microbiome varied little between HC and GBA-NMC but very significantly increased and decreased for enriched and depleted species, respectively, in PD relative to HC or GBA-NMC (Fig. 1f,g), supporting the view that it evolves mostly once PD is manifest.

In sum, over a quarter of the gut microbiome significantly changes in PD relative to HC, of which we distinguished two components. A major one evolves consistently from HC across GBA-NMC to PD, the extent of changes being lower in GBA-NMC than in PD. A minor component changes abruptly in overt PD. Because similar coherent changes in gut microbiome composition are observed in at-risk individuals, such as GBA-NMC, and in patients with PD, we suggest that the coherently altered species of the PD gut microbiome may represent a prodromal feature of PD (the ‘prodromal-PD microbiome’), possibly contributing to PD development.

### Associations of microbial alterations and clinical variables in PD

**Extended Data Fig. 2:**
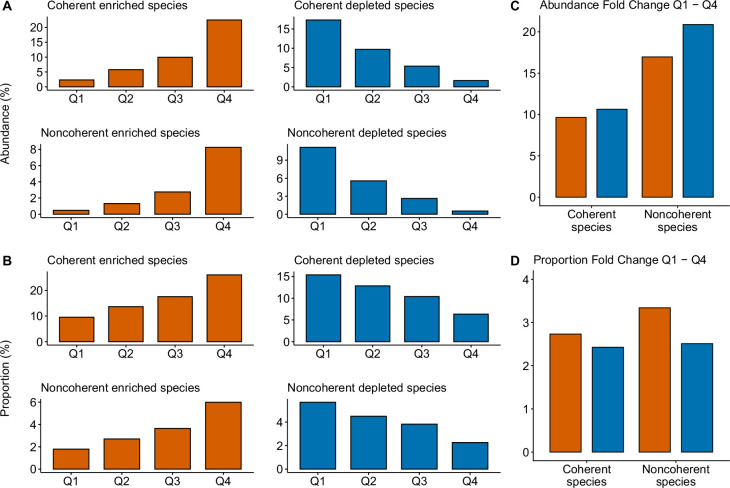
Microbiome variations in PD patients. **a** and **b**. Average values of abundance (**a**) and proportion (**b**) per participant (n = 271) of the 176 significantly differentially abundant species found between PD and HC (PD enriched and coherent n = 81, PD enriched and non-coherent n = 18, PD depleted and coherent n = 61, PD depleted and non-coherent species n = 16) over the quartiles (Q) of individual distribution (Q1, 2 and 4; n = 68, Q3; n = 67 patients). **c**. Abundance fold change (FC) between Q1 and Q4. **d**. Proportion FC between Q1 and Q4.

We estimated microbiome alterations by eight different tests, which capture related but different aspects of microbiome composition. The first four tests were based on abundance of different species types (coherent enriched or depleted; non-coherent enriched or depleted); the other four were based on proportion of the same species types. Abundance and proportion were computed as described in the Methods. Intuitively, an individual could have a higher proportion of coherent enriched species but a lower overall abundance of these species than another; the microbiome of the former would appear more altered than that of the latter by the test of proportion but less altered by the test of abundance. We ordered patients with PD by the fraction of the microbiome estimated by each of the eight tests and found that different quartiles vary greatly in all cases—Q1 and Q4 differed 10−20-fold by abundance and 2−3-fold by proportion (Extended Data Fig. 2a–d). This analysis shows considerable heterogeneity of microbiome composition among patients with PD.

**Fig. 2:**
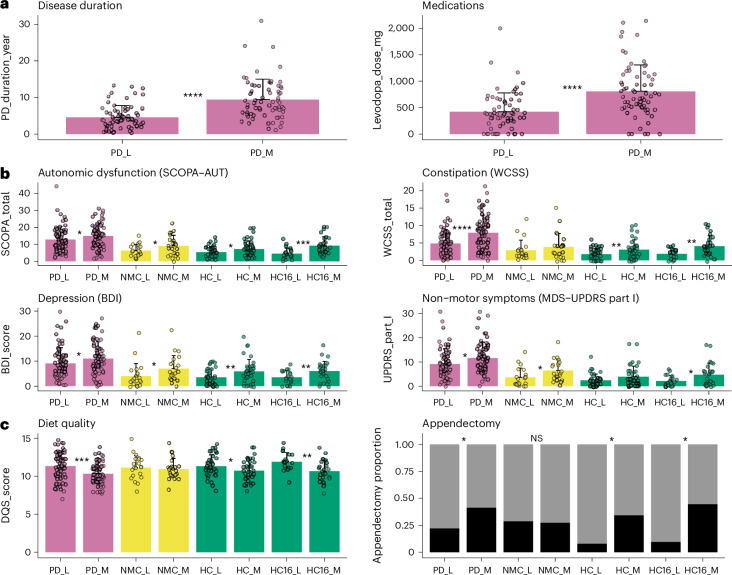
Association of microbiome alterations with clinical variables. Comparison of individuals with least (L) and more (M) altered microbiome across the study groups: for PD individuals, top and bottom quartiles of the distribution according to the abundance of coherent depleted species (*n* = 68 each); for GBA-NMC individuals, those below and above the median according to the proportion of coherent enriched species (*n* = 21 and *n* = 22, respectively); for HC individuals, top and bottom quartiles of the distribution according to the proportion of coherent enriched species (HC_L and HC_M, *n* = 38 each). HC individuals having PDMS-16 ≤ −5 (HC16_L, *n* = 21) and PDMS16 ≥ 3 (HC16_M, *n* = 27) are also shown. **a**, Disease-associated variables of patients with PD (disease duration and medication dosage). **b**, Clinical variables for all groups (automatic dysfunction, constipation, depression and non-motor symptoms). **c**, Health-related variables for all groups (DQS; appendectomy colored in gray if individuals did not undergo appendectomy and colored in black if individuals underwent appendectomy). The values of clinical variables are the means listed in Extended Data Table 1. Standard deviations and statistical significance are indicated by the thin bars, and stars indicate the significance level of the one-sided Studentʼs *t*-test for each comparison, except for appendectomy where comparisons are tested using two-sided *χ*^2^ test. Significance of *P* values: NS (not significant): *P* ≥ 0.05; *P < 0.05; **P < 0.01; ***P < 0.001; ****P < 0.0001. The exact *P* values are listed in Extended Data Table 1. BDI, Beck Depression Inventory; SCOPA-AUT, Scales for Outcomes in Parkinsonʼs Disease-Autonomic Dysfunction.

We hypothesized that higher gut microbiome alterations were associated with worse clinical features in patients with PD. To test this hypothesis, we compared clinical variables of individuals from quartiles with highest (*n* = 68) and lowest (*n* = 68) microbiome alterations, estimated by each of the eight tests. Extended Data Table 1 shows the results from the comparison using the abundance of coherent depleted species as test of microbiome alterations; all comparisons are displayed in Supplementary Table 5. Some variables were significantly different in less than 25% of comparisons (≤2/8 comparisons; for example, age or body mass index (BMI)); we considered them to be either weakly correlated or even not correlated with microbiome alterations. By contrast, certain variables were significantly different in more than 75% of comparisons (≥6/8 comparisons), and we suggest that they are highly correlated with microbiome alterations (Fig. 2 and in bold in Extended Data Table 1). They fell in two classes.

The first class corresponded to variables associated with disease severity, found to be higher in individuals in the top quartile of microbiome alterations, in agreement with our hypothesis. Among these, we detected depression, autonomic dysfunction, constipation and motor dysfunction. A higher percentage of individuals who underwent appendectomy prior to PD development was found in the top quartile; association of appendectomy and PD has been reported but remains controversial^21–24^.

The second class included variables associated with health, found to be worse in individuals with more altered microbiome, also in agreement with our hypothesis. These variables included cognitive and olfactory functions. Interestingly, variables related to nutrition, such as the Dietary Quality Score (DQS) or fruit and vegetable consumption, were also worse, suggesting a possible relation between food quality and milder disease profile.

**Extended Data Fig. 3:**
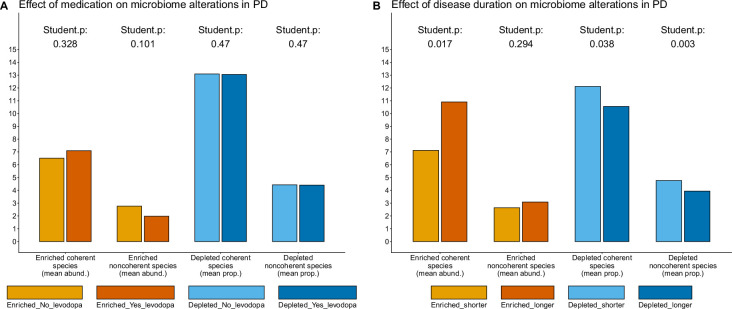
Effect of medication and disease duration on microbiome alterations in PD. **a**. Comparison of patients medicated (365 mgs, on average, n = 47) with drug naive (n = 32), having on average similar disease duration (2.02 and 2.01 years, respectively). The highest possible number of medicated patients to match the average disease duration of all non-medicated patients of our cohort was included, to maximize statistical power of the analysis. **b**. Comparison of patients with different disease duration, taking, on average, similar medication levels (602 mgs on average). Shorter disease corresponds to 4.2 years, on average, (n = 30) and longer to 7.2 years (n = 31). P-value was computed by the one-sided Student t-test.

We noted that the two most significantly differing variables between top and bottom quartiles were PD duration and levodopa equivalent daily dose (LEDD) (that is, the amount of antiparkinsonian medications), both associated with disease severity (Extended Data Table 1). We thus compared the microbiome of patients with PD medicated or drug naive, adjusting for disease duration, and the microbiome of patients with PD with different disease duration, adjusting for medication dose (Extended Data Fig. 3a,b). No significant differences were observed in the level of PD-enriched or PD-depleted species in medicated and non-medicated patients with the same disease duration, whereas significant differences were observed in patients with the same medication dose and different disease duration. We suggest that the microbiome evolves with the progression of the disease rather than in response to treatment.

### Associations of microbial alterations and clinical variables in disease-free GBA-NMC and HC

**Fig. 3:**
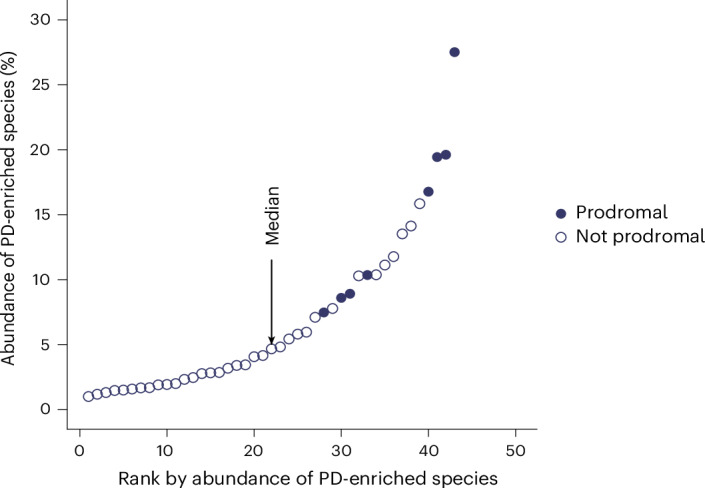
GBA-NMC individuals (*n* = 43) ranked by the abundance of PD-enriched species. Individuals assigned to the prodromal group by the MDS research criteria (*n* = 8) are represented by filled dots.

**Extended Data Fig. 4:**
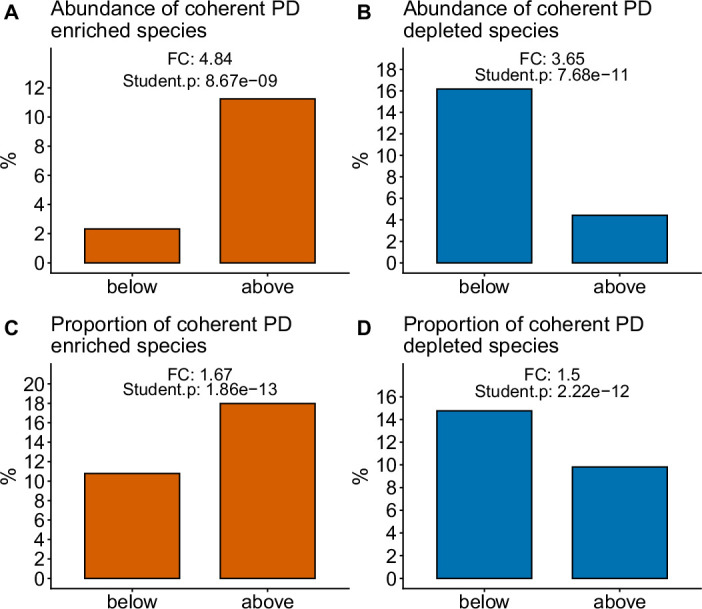
Microbiome variations in GBA-NMC. **a** and **c**. Average values of abundance (**a**) and proportion (**c**) of enriched coherent species above (n = 22) and below (n = 21) the median. **b** and **d**. Average values of abundance (**b**) and proportion (**d**) of depleted coherent species above (n = 21) and below (n = 22) the median. FC refers to Fold Change. P-value was computed by the one-sided Student t-test.

To investigate whether the PD microbiome signature was associated with any prodromal clinical profile in GBA-NMC, we compared clinical variables of GBA-NMC individuals with lower and higher microbiome alterations, below or above the median of the abundance and proportion of coherent PD-enriched/depleted species; expectedly, non-coherent species, which change significantly in abundance only in overt disease, were not informative (Extended Data Fig. 4; split around median rather than comparison of quartiles was used to conserve statistical power). We used the threshold of 75% comparisons, also used in patients with PD, to define variables highly associated with microbiome alterations. We identified numerous significantly different parameters, mainly representative of non-motor symptoms, which can be present in the prodromal phase (Fig. 2, Extended Data Table 1 and Supplementary Table 6). Among these, scores representing motor and non-motor disability and autonomic dysfunction were all significantly higher in individuals showing microbiome alterations above the median. Notably, all GBA-NMC individuals who were identified as prodromal PD, based on the MDS research criteria^16^, had the abundance of coherent PD-enriched species above the median, a highly significant bias (*χ*^2^ test, *P* = 4.7 × 10^−3^), and four were at the very top of the distribution (Fig. 3; *P* = 5.7 × 10^−4^). We conclude that GBA-NMC individuals presenting with the microbiome signature closer to PD are the ones showing the strongest prodromal profile, especially in terms of autonomic and peripheral nervous system dysfunction (body-first PD). Paralleling the observation with patients with PD, where the duration of overt disease appears to be strongly associated with microbiome alterations, we suggest that, in the GBA-NMC group, the progression toward overt PD is associated with progressive microbiome alterations and that a combination of clinical and microbiome assessments may help identify those individuals who will convert to disease.

**Extended Data Fig. 5:**
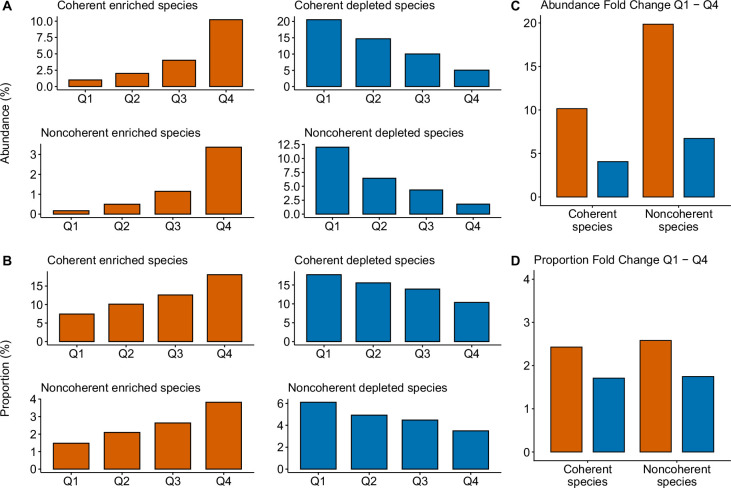
Microbiome variations in HC. **a** and **b**. Average values of abundance (**a**) and proportion (**b**) per participant (n = 150) of the 176 significantly differentially abundant species found between PD and HC (PD enriched and coherent n = 81, PD enriched and non-coherent n = 18, PD depleted and coherent n = 61, PD depleted and non-coherent species n = 16) over the quartiles (Q) of individual distribution (Q1 and Q4; n = 38, Q2 and Q3; n = 37 individuals). **c**. Abundance fold change (FC) between Q1 and Q4. **d**. Proportion FC between Q1 and Q4.

We then investigated microbiome alterations across quartiles of distribution in healthy individuals, using the same four tests based on coherent species (depleted and enriched; abundance and proportion), and found very significant differences, resembling those observed for patients with PD, albeit with lower values for the PD-enriched species and higher for the PD-depleted species in the extreme quartiles (Extended Data Fig. 5). Interestingly and notably, the comparison of clinical variables of HC individuals assigned to the extreme quartiles, denoted as lower (*n* = 38) and higher (*n* = 38) microbiome alterations for analogy with the PD and GBA-NMC groups (Fig. 2, Extended Data Table 1 and Supplementary Table 7), revealed significant differences of most variables differing in the GBA-NMC group, including depression and global motor and non-motor disability. Furthermore, these HC differed in eating habits (DQS and fruit and vegetable consumption) and appendectomy frequency, as observed for patients with PD. We conclude that microbiome alterations resembling those of patients with PD take place in a healthy population free of *GBA1* genetic risk, raising the possibility that, as in the individuals with *GBA1* variants, such alterations might be associated with progression toward overt disease. Of note is that, for both GBA-NMC and HC, one of the tests, based on the abundance of coherent species, revealed fewer differences than the other three (Supplementary Tables 6 and 7). We do not have an explanation for this observation, which indicates the value of using multiple tests to assess microbiome alterations, and we suggest that clinical variables significantly different by at least two of the other three tests correlate well with microbiome alterations in disease-free individuals (as reported in Extended Data Table 1).

### Correlation of microbial species and clinical variables

**Fig. 4:**
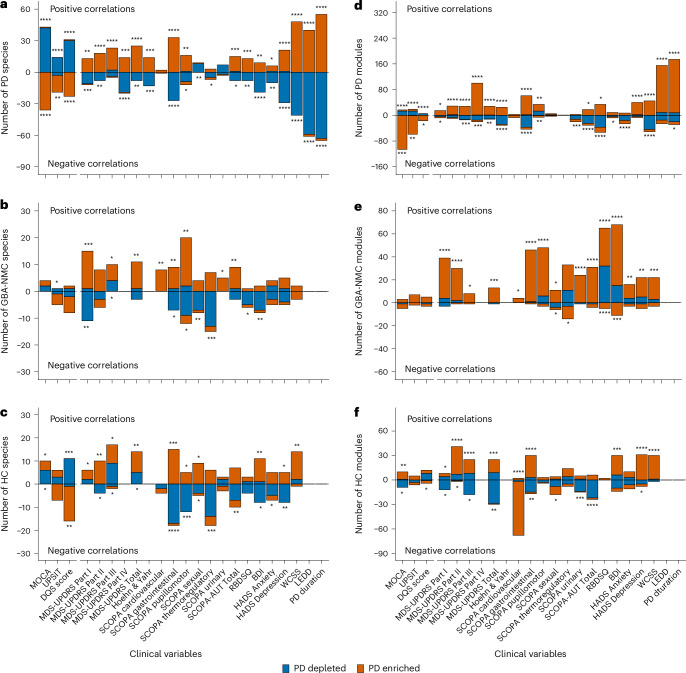
Correlation of microbial species and functional modules with clinical parameters. **a**−**c**, Number of microbial species significantly (Spearmanʼs correlation coefficient *P* < 0.05) correlated with different clinical parameters is indicated on the ordinate for patients with PD (**a**), GBA-NMC participants (**b**) and HC individuals (**c**). Orange and blue colors refer to species enriched and depleted in PD; positive and negative integers refer to positive and negative correlations, respectively. **d**−**f**, Number of functional modules correlated significantly (Spearmanʼs correlation coefficient *P* < 0.05) with different clinical parameters is indicated on the ordinate for patients with PD (**d**), GBA-NMC participants (**e**) and HC individuals (**f**). Orange and blue colors refer to modules enriched and depleted in PD; positive and negative integers refer to positive and negative correlations, respectively. Stars indicate the significance of the correlation bias of enriched and depleted species (two-sided *χ*^2^ goodness-of-fit test) on the positive and negative half of the graph, respectively. Significance of *P* values: *P < 0.05; **P < 0.01; ***P < 0.001; ****P < 0.0001. BDI, Beck Depression Inventory; HADS, Hospital Anxiety and Depression Scale; SCOPA-AUT, Scales for Outcomes in Parkinsonʼs Disease-Autonomic Dysfunction.

We next investigated whether the species that compose the altered microbiome might individually be correlated with clinical variables, focusing on the 176 species differentially abundant in HC and PD. In the PD group, abundance variations of 94% of species were significantly (*P* < 0.05) correlated with at least one clinical variable (Supplementary Fig. 1a and Supplementary Table 8); the number of species correlated with a variable is shown in Fig. 4a. PD-enriched species and PD-depleted species were associated positively and negatively, respectively, with disease severity (for example, non-motor symptoms such as constipation, autonomic dysfunction, depression and RBD) and, conversely, negatively and positively with health (cognition and olfactory function and DQS score).

**Extended Data Fig. 6:**
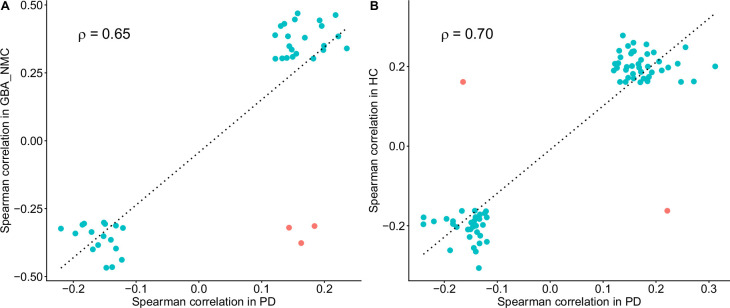
Significant Spearman correlation coefficients between species and clinical parameters in PD and GBA-NMC (A), or PD and HC (B). Blue dots indicate correlations with the same directions in both groups, and orange dots indicate correlations with different directions in the two groups. *ρ* denotes the Spearman’s correlation coefficient between the Spearman values with the same direction.

Notably, we found a similar profile of correlations between species and clinical variables in GBA-NMC (Fig. 4b), with variables associated with severity being mostly positively correlated with species enriched in PD and negatively with species depleted in PD (Supplementary Fig. 1b); the converse was observed for variables associated with health. However, the correlation bias was less clear than in the PD group (for example, cognition or constipation—Montreal Cognitive Assessment (MOCA) or Wexner Constipation Scoring System (WCSS), respectively), possibly because of lower statistical power due to the smaller group size and/or lower variance of the variables in the GBA-NMC group. The correlations involved 64% of the 176 species (Supplementary Table 9). Some 15% of correlations were between the same species and clinical variables as in patients with PD (Supplementary Table 10), and an overwhelming majority of those (39/42) had the same direction in both groups (Extended Data Fig. 6a). Other correlations were different, possibly due to relatively small variations of species abundances within a study group, resulting in a statistical significance in one group but not another.

Similar correlations were found in healthy individuals (Fig. 4c and Supplementary Table 11). The correlation bias was less clear than in the PD group, as observed in the GBA-NMC group, possibly for the same reasons. The correlations involved 77% of the 176 species, with 24% of them being between the same species and clinical variables as in the PD group (Supplementary Table 12) and showing the same direction in both groups (75/77; Extended Data Fig. 6b).

A notable conclusion from our analyses is that the microbial species correlated with disease severity in patients with PD appear correlated with clinical variables that estimate disease severity both in healthy individuals at higher risk of PD development due to their *GBA1* genetic background and in those without known *GBA1* genetic predisposition.

### Stratification of HC individuals using microbiome features

**Extended Data Fig. 7:**
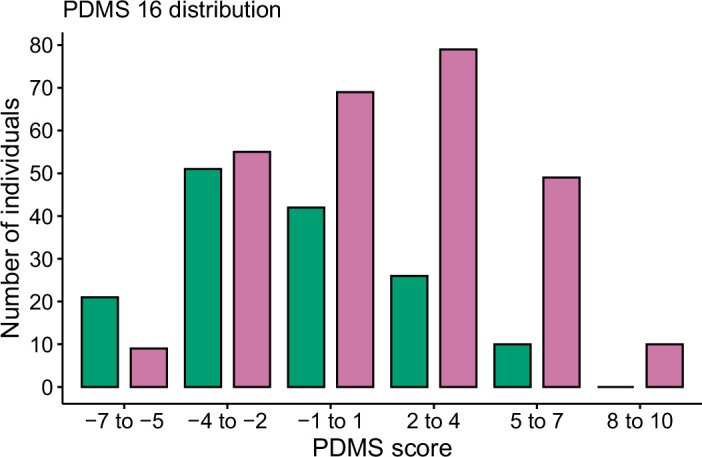
Distribution of PDMS-16 in HC (n = 150) and PD (n = 271) groups. Green and mauve bars refer to HC and PD, respectively.

In view of similar correlations between clinical variables and microbial species in all study groups, we explored the possibility of stratifying HC individuals who may be closer to disease development by the microbial species they harbor. To do so, we first selected the species involved in the same significant correlations with clinical variables in PD and the disease-free GBA-NMC (33 species). Among those, we then selected species with the highest prevalence difference in PD relative to HC (over 10%), assuming that they should be the best indicators of disease-related microbiome alterations, and the species correlated coherently with PD duration in patients, hypothesizing that they indicate alterations of the microbiome due to progression of disease in patients and, thus, possibly approaching the disease in HC. As a result, we identified 16 species, of which 10 were enriched and six were depleted in PD (Supplementary Table 13). By summing the number of PD-enriched species and deducing the number of PD-depleted species that each individual harbored, we computed a score, termed Parkinsonʼs Disease Microbiome Score-16 (PDMS-16), referring to the number of species used. PDMS-16 distribution among HC and PD individuals is shown in Extended Data Fig. 7.

We then compared clinical variables between the subgroup of HC presenting with the highest PDMS-16 (3−7; *n* = 27, 18% of the group) with (1) 21 individuals with the lowest PDMS-16 score (−5 and −6) and (2) the remainder of the HC group (*n* = 123). The individuals with the highest PDMS-16 score had more severe depression and anxiety, autonomic dysfunction and constipation, consumed less healthy food and had a more frequent history of appendectomy (Fig. 2 and Extended Data Table 1). We conclude that the PDMS-16 score can identify individuals with a clinical profile closer to that of patients with PD than the remainder of the HC group in our study population, and we suggest that, as proof of concept, it could be a lead for developing a broadly applicable, species-based score of microbiome alterations indicative of PD risk in disease-free individuals, when much larger, adequately phenotyped cohorts become available.

### Alterations of microbiome functions in PD and GBA-NMC

Comparison of HC with patients with PD revealed that 146 of the 357 complete functional microbial modules from three sources (Kyoto Encyclopedia of Genes and Genomes (KEGG), Gut Metabolic Modules (GMM) and Gut Brain Modules (GBM)) were differentially abundant at *q* < 0.05: 121 were enriched in PD and 25 were depleted (Supplementary Table 14). The PD microbiome was enriched for modules related to neurotransmitters metabolism (dopamine, acetylcholine and GABA), nucleic acid degradation and amino acid degradation (threonine, phenylalanine and putrescine). Enrichment of the modules for dopamine degradation and DOPAC synthesis (MGB023 and MGB024, respectively) could possibly be due to levodopa treatment of patients with PD, as Actinobacteria reportedly can degrade and use dopamine^18^. The degradation pathway of the bacterial byproduct of protein degradation, p-cresol, was also found to be enriched in our PD cohort. The modules depleted in PD were involved in riboflavin biosynthesis, dietary carbohydrate degradation (lactose and mannose) pathways and the conversion of acetyl-coenzyme A into acetate pathway. These findings, combined with the enrichment in pathways implicated in the conversion of acetate into acetyl-coenzyme A, or of propionate into succinate, and the tricarboxylic acid cycle, support a metabolic shift from carbohydrate fermentation toward proteolysis as an energy source, in line with previous findings^17,20^.

**Extended Data Fig. 8:**
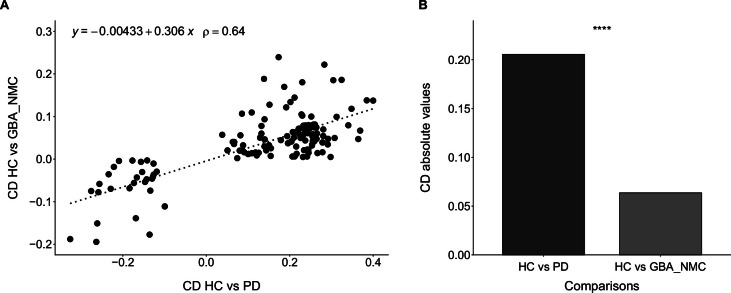
Variations of functional modules in PD and GBA-NMC relative to HC. **a**. Correlation of Cliff Delta for coherently varying modules, either enriched or depleted. ρ denotes Spearmanʼs correlation coefficient. **b**. Absolute values of Cliff delta in HC vs PD or GBA-NMC. P value was computed by the one-sided Student test, p = 3.241E-09. Significance of P values: *, p < 0.05; **, p < 0.01; ***, p < 0.001; **** p < 0.0001.

The comparison of HC and GBA-NMC individuals identified only six significantly different modules at *P* < 0.05. This low number may reflect, in part, fewer alterations of functions in the GBA-NMC group and, in part, insufficient statistical power. We, therefore, examined the coherence of Cliffʼs *δ* variation of the modules significantly different in HC and PD in the GBA-NMC group. Of the 180 modules significant at *P* < 0.05, 143 varied coherently and 37 varied non-coherently (Supplementary Table 14), a very significant bias (*χ*^2^ test, *P* = 2.8 × 10^−15^). Cliffʼs *δ* values of the coherently varying modules were significantly correlated (Extended Data Fig. 8a) and significantly higher in the HC versus PD comparison than in the HC versus GBA-NMC comparison (Extended Data Fig. 8b).

We conclude that functional potential of the microbiome displays variations like those observed for the taxonomic differences.

### Correlations of microbiome functions and clinical variables

Abundance variations of 92% of modules were significantly correlated with at least one of 25 clinical variables in the PD group (Supplementary Table 15). Correlations were biased in a similar way observed for individual species: enriched modules largely correlated positively with variables related to the severity of the disease and negatively to those related to health, whereas the depleted modules had an opposite bias (Fig. 4d).

Similar correlation profiles were observed for the other two study groups, GBA-NMC and HC (Fig. 4e,f and Supplementary Tables 16 and 17), albeit with a lower number of modules, likely due to lower statistical power (58.3% and 72.5% for GBA-NMC and HC, respectively).

We conclude that, expectedly, functional potential of the microbiome correlates with clinical variables similarly to species that encode it.

### Validation of microbiome alterations in independent cohorts

We examined three different publicly available cohorts composed of patients with PD and healthy individuals where stool microbiome was analyzed by shotgun metagenomics. One was from the United States (PD, *n* = 491; HC, *n* = 234)^12^, one from Turkey (PD, n = 69; HC, *n* = 17)^25^ and one from Korea (PD, n = 78; HC, *n* = 68)^26^. There were *n* = 498, *n* = 606 and *n* = 572 metagenomic species, respectively, present in at least 10% of the cohort individuals (Supplementary Tables 18−20), of which the large majority was also present in our cohort (*n* = 408, 81.9%; *n* = 474, 78.2%; and *n* = 448. 78.3%, respectively). By abundance, these common species represented 94.2%, 78.7% and 87.8% of the microbiome, respectively.

**Fig. 5:**
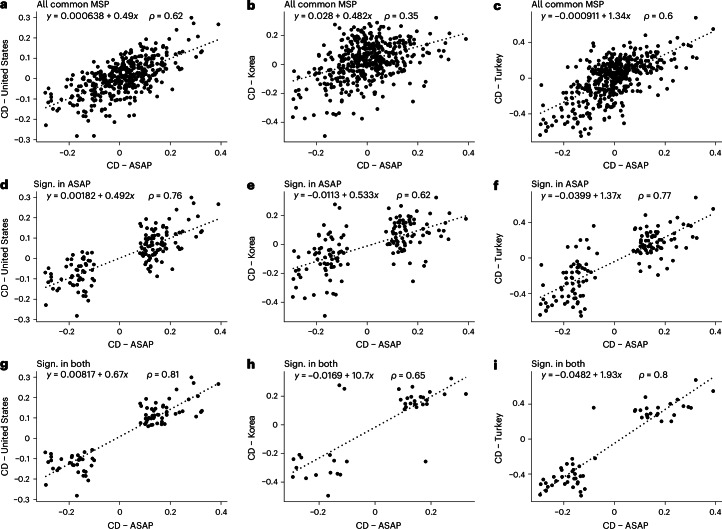
Correlation of Cliffʼs *δ* for species common to three independent cohorts compared to the one cohort studied here (named ASAP). **a**,**d**,**g**, United States cohort. **b**,**e**,**h**, Korean cohort. **c**,**f**,**i**, Turkish cohort. Top panels: all species. Middle panels: 176 species significant in our study (two-sided Wilcoxon *P* < 0.05). Bottom panels: species significant in both cohorts (two-sided Wilcoxon *P*< 0.05). Wilcoxon values are displayed in Supplementary Tables 1 and 18–20. *ρ* denotes Spearmanʼs correlation coefficient. CD, Cliffʼs *δ*; Sign., significant.

Cliffʼs *δ* values computed for the HC/PD comparisons in each of the three cohorts were well correlated with the Cliffʼs *δ* values observed in our cohort, for all species common between a given cohort and our cohort (Fig. 5a–c). The correlations were higher for species significantly different in our cohort only (*P* < 0.05; Fig. 5d−f) and raised even further for species significantly different in both our cohort and another given cohort (Fig. 5g–i). Furthermore, the direction of Cliffʼ *δ* values for species significant in both cohorts was fully conserved in two cohorts (United States and Turkey) and 93.3% conserved in one cohort (Korea). This indicates that similar microbiome alterations take place in PD worldwide, given the geographic diversity of the cohorts studied, and that they extend to species that do not appear significantly different (*P* > 0.05).

**Extended Data Fig. 9:**
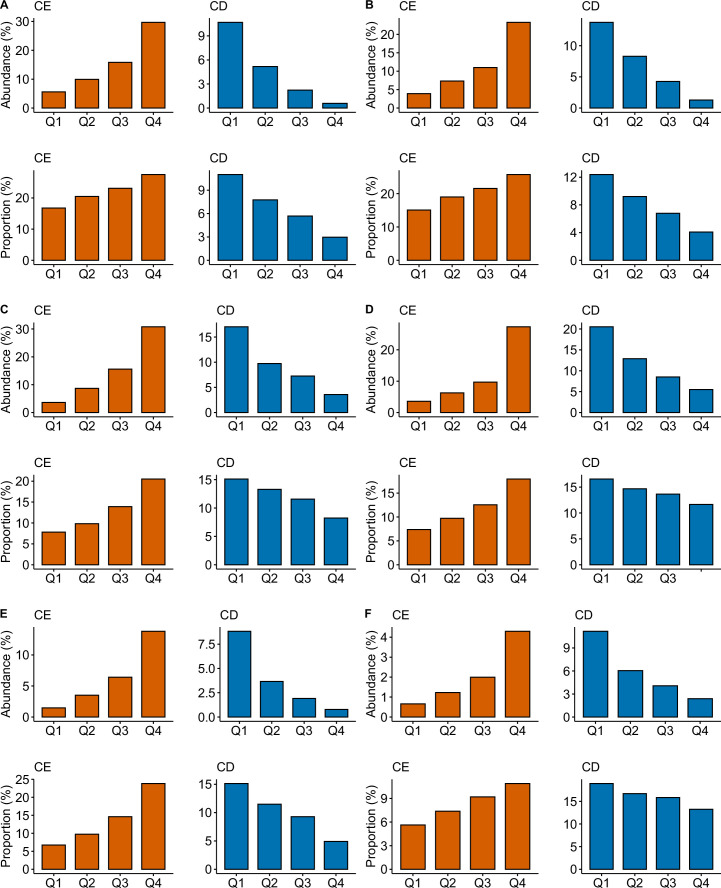
Microbiome variations in PD patients and HC. **a, b**: USA cohort (PD, n = 491; HC = 234); **c, d**; Korean cohort (PD, n = 78; HC = 68); **e**, **f**: Turkish cohort (PD, n = 69; HC = 17). Left panels (**a, c, e**): PD patients, right panels (**b, d, f**): HC. Average values of abundance (top) and proportion (bottom) of significantly differentially abundant species found in PD/HC comparisons of our cohort for two species types (CE, coherent enriched; CD, coherent depleted). Orange and blue refer to coherent enriched and depleted species, respectively.

We next examined the intragroup heterogeneity of the microbiome in the three cohorts, using as lead the coherent PD-enriched and PD-depleted species defined in our cohort. Heterogeneity like the one we observed in our cohort was found in all other cohorts (Extended Data Fig. 9), indicating that, among both patients and healthy individuals, some have more altered microbiomes than others. These observations largely validate conclusions from our cohort analysis.

Clinical phenotypes were available only for the Turkish cohort (Supplementary Table 21), where microbiome profiles of sufficient quality were generated for 69 PD individuals and for 17 HC individuals with our pipeline, using the published sequencing reads. We first analyzed the patients with PD, comparing the clinical variables of the top and bottom terciles with least and most altered microbiome (*n* = 23 each; Supplementary Table 22). Significant differences were observed for most variables (17/24; 71%) in at least some tests; the highest number of variables (12/24, 50%) was found using abundance of depleted non-coherent species. The disease duration was significantly different (*P* < 0.05) in seven of eight tests, chiming with the observations in our cohort. Two other variables, the Neuropsychiatric Inventory (NPI-severity) and Wechsler Memory Scale-Visual Reproduction (WMS-VR_Short-Term_Memory), were also significantly different in the large majority (six or more) of tests. Other variables were significantly different in fewer tests, possibly because of the lower statistical power than in our cohort, but their tendency was in the expected direction—variables associated with disease severity were higher for individuals with more altered microbiome, whereas variables associated with health were lower. We conclude that the association of clinical parameters and microbiome alterations in PD that we describe for our cohort is largely validated in an independent cohort.

Analysis of the HC group (*n* = 17), split around the median, yielded few clear results (Supplementary Table 23), likely because of a small group size and the limited nature of clinical data available (cognitive and neuropsychiatric scales and not other recognized prodromal features of PD). We conclude that fuller validation of our observations for disease-free individuals may have to await larger, appropriately phenotyped cohorts.

## Discussion

Using combined clinical and metagenomics data from a large population of individuals with overt PD and those harboring genetic predisposition to PD (GBA-NMC), we identified similar, coherent alterations in the gut microbiome profile of patients with PD, in genetically at-risk individuals presenting a higher burden of prodromal symptoms and in approximately 20% of healthy individuals without known genetic predisposition for PD. This was possible by the examination of the coherence of microbiome alterations between the study groups, in addition to the more common assessment of their statistical significance, allowing detection of small abundance alterations of many species and not only large alterations of a small number of species. This innovative paradigm of microbiome analysis may be useful in studies with a design like ours, where at-risk groups are compared with both healthy and diseased groups, and might guide the design of studies yet to come, as it informs on a much larger fraction of the microbiome that might be changed.

Previous studies conducted on other individuals at high risk of developing PD (for example, isolated RBD/idiopathic RBD (iRBD)) also found that the gut microbiome of patients with iRBD was at an intermediate state between healthy and early-stage PD individuals^27^. The body-first model of PD has gained attention in recent years, and results from our correlation analyses support the hypothesis that microbiome alterations might occur in the prodromal phase of body-first subtype of PD. Clinically, patients with body-first PD are characterized by a higher burden of non-motor symptoms, in particular autonomic symptoms^28^. Our results showed that PD individuals with the most marked microbiome alterations had more severe non-motor symptoms. In non-parkinsonian individuals, we found similar results, with autonomic dysfunction and depression being more severe in those individuals with more marked PD-like microbiome alterations. Notably, the four GBA-NMC individuals with the highest abundance of coherently PD-enriched species met the criteria for prodromal PD; this fits the expectation that 10–20% of *GBA1* variant carriers will develop overt disease (four individuals out of *n* = 43 included in our study) during their lifetime. These data highlight the importance of screening populations at risk of PD (*GBA1* variant carriers, iRBD, etc.) based on a combination of clinical, genetic and environmental factors and then stratify them based on specific markers, such as their gut microbiome profile. The lifetime risk for PD in the general population is approximately 3%^29^; therefore, discovering PD-like microbiome alterations in approximately 20% of participants means that other genetic and/or environmental factors interact with and influence PD development.

The present study has some limitations. First, this is a cross-sectional study, and longitudinal studies are needed to follow-up the potential conversion to PD in some individuals. Second, the relatively small number of GBA-NMC individuals might have undermined the results. Third, the lack of independent cohorts with adequate phenotyping of disease-free individuals limited the full validation of our results, even if we could show intergroup and intragroup microbiome alterations similar to the ones we report in three independent cohorts with wide geographic distribution (United States, Turkey and Korea) and the association of the alterations with clinical phenotypes in patients with PD in the one with clinical data available.

In conclusion, we found that both genetically predisposed and healthy individuals without genetic risk can have gut microbiome changes like those typical of PD. As these alterations appear associated with disease progression in patients and with the development of prodromal features in individuals at risk of PD, screening of healthy individuals using microbiome analysis as proposed here may enable identification of healthy individuals at higher risk of neurodegeneration. Full assessment of the power of such an approach to combat PD will have to await longitudinal follow-up of sufficiently large groups of individuals and for adequate length of time.

## Methods

### Study design

This was a cross-sectional, multicenter, international study with participants recruited from the United Kingdom and Italy. Four groups of individuals were enrolled: patients with PD carrying a variant in the *GBA1* gene; patients with PD not carrying a variant in the *GBA1* gene; HC individuals; and non-manifesting carriers of a *GBA1* variant (that is, not diagnosed with PD (GBA-NMC)). We aimed to recruit groups of individuals who were age matched and sex matched, where sex was defined based on self-report. As HC, we aimed to recruit the partners of patients with PD to mitigate differences in diet and lifestyle habits and, therefore, the influence of these factors on the gut microbiome. Some HC individuals who were not partners/spouses of patients with PD were also recruited as population control subjects.

### Recruitment strategy and *GBA1* gene sequencing

Recruitment strategy of study participants was performed, as previously described^30^. Participants from the United Kingdom were recruited via the RAPSODI GD and PD Frontline portals, from University College London (UCL). This study was approved by the local ethics committees (London – Queen Square REC: 15/LO/1155), and all participants signed informed consent upon enrollment. In the United Kingdom, the analysis of the *GBA1* gene was performed on saliva samples collected with the DNA OG-500 kit from DNA Genotek (https://www.dnagenotek.com/row/products/collection-human/oragene-dna/500-series/OG-500); after collection, following the manufacturer’s instructions (https://www.dnagenotek.com/row/support/collection-instructions/oragene-dna/OG-500andOG-600), samples were posted back from study participants to UCL. DNA extraction and long-range sequencing of an 8.9-kb amplicon, including all coding exons and introns of the *GBA1* gene, were performed using bespoke primers on the Oxford Nanopore MinION platform, as previously described^30^, partially at the laboratories of the Department of Clinical and Movement Neurosciences, UCL Institute of Neurology, United Kingdom, and partially at the Exeter Clinical Laboratory International, United Kingdom, a diagnostic clinical laboratory accredited by the National Accreditation Body (UKAS 8092). The *GBA1*-positive results were confirmed by Sanger sequencing at Exeter Clinical Laboratory International. Two healthy participants with synonymous variants (G264 = /WT, and V499 = /WT) have been considered as WT/WT for the *GBA1* gene, as synonymous variants are generally considered to be silent for any effect, because they do not change the amino acid sequence of the protein^31^. Participants from Italy were recruited from two neurology tertiary centers: the IRCCS Mondino Foundation in Pavia and the Neurology Unit, Neuromotor & Rehabilitation Department, Azienda USL-IRCCS in Reggio Emilia. The sequencing of the *GBA1* gene in the Mondino Pavia cohort was performed by a next-generation sequencing (NGS)-based method, which includes the selective amplification of the whole *GBA1* gene in one long polymerase chain reaction (PCR) fragment (6 kb) followed by Nextera sequencing and a customized bioinformatics pipeline aimed at masking the *GBAP1* pseudogene. The *GBA1*-positive results were confirmed by conventional Sanger sequencing through *GBA1* amplification in three overlapping fragments using specific primer pairs. Pathogenic variants in 15 PD-related genes linked to autosomal dominant PD (*SNCA*, *LRRK2*, *VPS35* and *GBA1*), X-linked PD (*RAB39B*) and autosomal recessive PD (*PRKN*, *PINK1*, *PARK7*, *ATP13A2*, *PLA2G6*, *DNAJC6*, *SYNJ1*, *FBXO7*, *VPS13C* and *PTRHD1*) were also evaluated using NGS-based sequencing of PD gene virtual panel as well as multiplex ligation-dependent probe amplification (MLPA) analysis (SALSA Kit P51-P52; MRC Holland). In the Reggio Emilia cohort, the sequencing of the *GBA1* gene for patients with PD was performed by using a *GBA1*-specific long-range PCR with subsequent *GBA1* exon-specific PCR and NGS of the resulting products, as previously described^32^. Patients with PD were also tested for 11 pathogenic or likely pathogenic variants in the *LRRK2* gene, and, if negative, 50 target genes were analyzed by means of NGS-based sequencing of PD gene virtual panel^32^. For non-parkinsonian individuals, the genetic sequencing of *GBA1* was performed either by long PCR approach or NGS-based method^33,34^. This study was approved by the local ethics committees (of Pavia, code P-20210009687, and of Area Vasta Emilia Nord, code 2021/0092531), and written informed consent was obtained upon enrollment from all study participants.

### Clinical evaluation

Participants were invited for an in-person assessment in the clinic. Information about demographics and family history of PD was collected from each participant. Each participant underwent a clinical assessment to evaluate motor and non-motor symptoms of PD, including autonomic function, RBD, olfaction, mood and cognition. The selected scales and questionnaires, with related references, are reported in Supplementary Appendix B. For patients with PD, information about time at diagnosis and medications was collected, and the LEDD was calculated^35,36^.

The MDS diagnostic criteria for prodromal PD were applied to the GBA-NMC group using the online Prodromal PD Calculator (https://www.movementdisorders.org/Prodromal-PD-Calculator.htm), as reported in the main text. Combining the estimated prior probability of PD—that is, the age-adjusted prevalence of PD—with the total likelihood ratio of prodromal PD obtained by multiplying the likelihood ratios of each marker, a post-test probability percentage of developing PD can be obtained for each individual. In this study, the following markers were used: age, sex, coffee consumption, smoking status, *GBA1* genetic status, diagnosis of type II diabetes mellitus, physical inactivity, presence of RBD (as defined by screening questionnaire), history of excessive daytime somnolence, olfactory loss, constipation, urinary dysfunction, severe erectile dysfunction, depression, cognitive deficit and subthreshold parkinsonism defined as MDS-UPDRS part III total score greater than 6 (excluding postural and action tremor).

All participants provided information about their weight and height, and BMI was calculated. Participants were asked to provide information about their dietary habits by completing a short form of a food frequency questionnaire that was previously validated against an extensive food quality questionnaire in a UK-based population^37^. A DQS based on fruit, vegetable, oily fish, non-milk extrinsic sugar (NMES) and fat intake was calculated using the DQS calculator provided by the authors.

Study data were collected and managed using REDCap electronic data capture tools hosted at UCL and the IRCCS Mondino Foundation^38^.

### Sample collection

Participants were asked to collect a fecal sample at home, using the sample collection kit OMNIgene•GUT | OM-200, purchased from DNA Genotek (https://www.dnagenotek.com/row/products/collection-microbiome/omnigene-gut/OM-200). Participants were instructed on how to collect and return the samples within a maximum of 7days from collection (https://www.protocols.io/view/stool-sample-collection-protocol-261gerypjl47/v1). The OMNIgene•GUT | OM-200 stabilizes microbial DNA from feces. Returned samples were stored in a freezer at −80 °C until transfer to INRAE, MetaGenoPolis, Paris laboratories for analyses. Individuals with reported use of antibiotics during the month prior to collection of fecal samples were excluded from the metagenomics analysis.

### Statistical analysis for clinical data

Categorical variables were compared using *χ*^2^ test. Continuous variables are presented as means ± s.d., unless otherwise specified. Group demographics and continuous clinical variables were compared using two-sided *t*-test or non-parametric Wilcoxon rank-sum two-sided test for two-group comparison (HC versus GBA-NMC, HC versus PD and GBA-PD versus idiopathic PD). For REM Sleep Behavior Disorder Screening Questionnaire (RBDSQ) scores, data were categorized using clinically validated thresholds, and binary regression was used to determine associations between categorized variables and the grouping variable. MOCA scores were analyzed using both a linear regression model and a binary regression model, adjusted for covariates (age, sex and education level). For University of Pennsylvania Smell Identification Test (UPSIT) scores, a linear regression model was applied with adjustment for covariates (age and sex). The Benjamini−Hochberg procedure for multiple testing was applied for the comparison of clinical features (19 variables for HC versus GBA-NMC or for GBA-NMC versus PD, and 20 variables for GBA-PD versus idiopathic PD, as indicated in Table 1 and Supplementary Appendix A, respectively). Statistical analyses of clinical data were performed using R (version 4.2.3; https://www.r-project.org/).

### DNA extraction and high-throughput sequencing

Frozen fecal materials were aliquoted to ≤1,000 µl with or without the addition of a liquefaction liquid (OMNIgene Liquefaction Reagent OM-LQR; DNA Genotek) to facilitate sample recovery. DNA extraction was performed following the procedure described in https://www.protocols.io/view/protocol-for-whole-shotgun-metagenomics-pipeline-f-5qpvo9ox7v4o/v1. The samples were transferred into a deep-well plate containing 400 µl of 0.1-mm glass beads (not in suspension) and centrifuged at 3,486*g* for 20 minutes prior to discarding the supernatant and adding 250 µl of guanidium thiocyanate, 40 µl of N-lauroyl sarcosine (10% solution) and 500 µl of N-lauroyl sarcosine (5% solution in 1× PBS) to the samples. Subsequently, the sample plate was incubated at 70 °C in a thermomixer for 1 hour, with stirring at 1,400 r.p.m. After centrifugation of the plate at 3,486*g* for 5 minutes, the lysate was collected in a new plate. The pellet of the previous plate was then washed with 500 µl of TENP (50 mM Tris-HCL, 20 mM EDTA, 10 mM NaCl, saturated with PVPP). The plate was then vortexed and centrifuged at 3,486*g* for 5 minutes, after which the recovered lysate was pooled with the previous one. Finally, the final lysate was centrifuged for a 10-minute period at 3,486*g*, after which 800 µl was collected in a new plate. This plate was employed for purification with magnetic beads on the QIASymphony. The used protocol was designed for MetaGenoPolis with the Qiagen DSP Virus/Pathogen Kit. DNA was quantified using Qubit Fluorometric Quantitation (Thermo Fisher Scientific) and qualified using DNA size profiling on a Fragment Analyzer (Agilent Technologies). Either 500 ng or 1 μg of high-molecular-weight DNA (>10 kbp) was used to build the library. Shearing of DNA into fragments of approximately 150 bp was performed using an ultrasonicator (Covaris), and DNA fragment library construction was performed using Ion Plus Fragment Library and Ion Xpress Barcode Adapters Kits (Thermo Fisher Scientific). Purified and amplified DNA fragment libraries were sequenced using the Ion Proton Sequencer (Thermo Fisher Scientific), with a minimum of 20 million high-quality 150-bp reads generated per library^39^.

### Read mapping

We performed quality control check to remove any low-quality sequences using AlienTrimmer software (version 2.0)^40^, with the following parameters: ‘-k 10 -l 45 -m 5 -p 40 -q 20’, and potential host-related reads with Bowtie 2 version 2.5.1 (ref. ^41^) and using the human reference genome *Homo sapiens* T2T-CHM13v2.0 (accession GCF_009914755.1)^42^. High-quality reads were mapped onto the 10.4 million gut human gene reference catalog version 1 (10.15454/FLANUP)^43^ and the 8.4 million human oral microbial catalog version 1 (ref. ^44^), using Meteor software^45^. Read mapping was performed in a two-step procedure, using an identity threshold of 95% to the reference gene catalogs. First, unique mapped reads were attributed to their corresponding genes. Second, shared reads were weighted according to the ratio of unique mapping counts. A downsizing procedure was performed to normalize gene counts between samples by randomly selecting a subset of reads depending on the sequencing depth (18 million reads for an average 24 million reads depth sequencing). The gene abundance table was then normalized using the fragments per kilobase of transcript per million mapped reads (FPKM) strategy and analyzed using the MetaOMineR (momr) R package (version 1.31, https://forge.inrae.fr/metagenopolis/momr)^46^. Finally, we performed a final quality control validation on the MSP species composition using CroCoDeEL methodology (https://github.com/metagenopolis/CroCoDeEL)^47^, combined with a Spearmanʼs correlations threshold of >0.65, to remove any low-quality samples, and yielding the final cohort of 464 individuals (*n* = 271 PD, *n* = 150 HC and *n* = 43 GBA-NMC).

### MSP microbial species determination

The 10.4 million gut gene and the 8.4 million oral gene catalogs were previously organized into 1,990 and 853 MSP species^44,48–50^ that correspond to clusters of co-abundant genes used as proxies for microbial species and containing core and accessory genes. We removed duplicated species obtained from the catalogs, yielding a total of 2,055 MSPs, further filtered at a 10% occurrence threshold for a final MSP species count at 627. Taxonomical annotation was assigned using the Genome Taxonomy Database GDTB R07-RS207 (ref. ^51^), using an in-house pipeline as described below. First, all genes were aligned on public databases (National Center for Biotechnology Information (NCBI) and Whole Genome Shotgun (WGS))^52^ using Blast^53^. MSPs were annotated with the lowest taxonomical rank (from species to superkingdom) that brought consensus in at least 50% of its genes. To avoid misleading annotations due to error in databases, for each gene the 20 first hits were considered. MSP definition and taxonomy are available from Data INRAE (10.15454/WQ4UTV and 10.15454/FLANUP). Relative abundance of a given MSP was computed as the mean abundance of its 100 ‘marker’ genes (that is, the genes that correlate the most altogether). If fewer than 10% of ‘marker’ genes were seen in a sample, the abundance of the MSP was set to 0. MSP richness was computed as the number of detected MSP species in a particular sample, before proceeding to occurrence filtering.

### Microbial functional potentials determination

To assess the functional potential of the gut microbiota at the module level, we used Meteor software, which includes several functional databases as previously described^54^. Three databases were used to predict gene functions: KEGG^55^, eggNOG database (version 3.0)^56^ and TIGRFAM (version 15.0)^57^. First, genes of the catalogs were annotated using KEGG107 database using DIAMOND^58^ and further clustered into functional pathway modules according to KEGG Orthology groups, GMM^59^ and GBM^60^, the latter using the annotations from the three KEGG, enggNOG and TIGRFAM datasets. Second, KEGG, GMM and GBM modules were reconstructed in each MSP using their reaction pathways based on their detected annotated KEGG Orthology genes. GMM and GBM functional modules were selected because they are specific to gut bacterial and gut−brain axis functions. For each pair of MSP/individual, the completeness of any given functional module was calculated by considering the set of genes detected in the MSP of each individual and the MSP completeness in each individual. For a given MSP in a specific individual, completeness of the modules was corrected by the abundance of the MSP. After correction, functional modules in each MSP/individual were considered as complete if at least 90% of the involved reactions were detected. Abundance of functional modules in each sample was computed as the sum of the MSP abundances containing the complete functional module.

### Computational analysis

Statistical analyses and figure computing were conducted using R environment version 4.4.1 (https://www.r-project.org/) unless stated otherwise.

The comparison of species and functional modules between groups was performed using the testRelation2 function from the momr package (version 1.31)^61^. This function generates a matrix that provides statistical results calculated with the two-sided Wilcoxon rank-sum test, with the Benjamini−Hochberg procedure for multiple testing, and quantifies the magnitude of these differences using Cliff’s *δ* for each species and functional module.

Enrichment of species from the same taxon (ranging from phyla to genera) among HC or patients with PD was assessed by the *χ*^2^ test using Excel, by comparing the observed number of species in the two groups with the number expected if they were equipartitioned.

Coherent and non-coherent enriched or depleted species and functional modules were identified based on the testRelation2 output for the HC versus PD and the HC versus GBA-NMC comparisons, using Cliff’s *δ* direction as guide.

All linear models were performed using the ‘lm’ function from the ‘stats’ R package (version 4.4.1)^62^. Regression plots and bar plots displaying Cliff’s *δ* values of coherent enriched and depleted species in the comparison HC versus PD and HC versus GBA-NMC were created using the ggplot2 package (version 3.5.1)^63^, and the equation formula was generated with stat_poly_eq from the ggpmisc R package (version 0.6.0)^64^.

The contribution of coherent and non-coherent species (enriched and depleted) to the microbiome was evaluated by calculating their proportion and abundance for each sample. Proportion was computed as the number of cognate species present in the sample divided by the total number of species in the sample. Abundance was computed as total abundance of cognate species divided by abundance of all species in the sample. Significance of the difference of abundance and prevalence between the study groups was estimated by the one-sided Studentʼs *t*-test performed with the t_test function from the rstatix R package (version 0.7.2)^65^.

Abundance and prevalence of cognate species in the quartiles of PD and HC samples were determined by first sorting the samples by a relevant parameter (abundance or prevalence of coherent or non-coherent, enriched or depleted species) and then computing the mean abundance and prevalence of each species type for the four quartiles and the average fold change of abundance and prevalence between the first and fourth quartiles.

Abundance and prevalence of cognate species above and below the median in GBA-NMC samples were determined by first sorting the samples by a relevant parameter (abundance or prevalence of coherent enriched species) and then computing the mean abundance and prevalence of each species type.

For each clinical variable, the significance of the difference between the study groups was assessed by the one-sided Studentʼs *t*-test with the t_test function.

Correlation between abundance of individual species or functional modules and clinical variables was computed by pairwise Spearmanʼs rank correlation tests, using the cor.test function from the ‘stats’ R package. For each clinical variable significantly correlated with species and modules (*P* < 0.05), the bias of PD-enriched and PD-depleted species was assessed by a *χ*^2^ goodness-of-fit test. For each study group, bar plots were created to display the number of species and functional modules showing positive and negative correlations with each clinical variable. The plots were generated using the ggplot2 package, with data manipulation facilitated by the dplyr package (version 1.1.4)^66^, the tibble package (version 3.2.1)^67^ and the tidyr package (version 1.3.1)^68^ and layout adjustments handled by the grid package (version 4.4.1)^69^. Finally, the bar plots were combined into a single visualization using the ggarrange function from the ggpubr package (version 0.6.0)^70^ and the wrap_plots function from the patchwork package (version 1.2.0)^71^.

Heatmaps illustrating the correlation values between species and clinical variables were generated using the Heatmap, Legend and packLegend functions from the ComplexHeatmap package (version 2.20)^72^. The color gradient was created using colorRamp2 from the circlize R package (version 0.4.16)^73^. Pairwise Spearmanʼs rank correlation tests were performed using the rcorr function from the R package Hmisc (version 5.1.3)^74^.

Regression plots were created using the ggplot and stat_poly_eq functions. For each group, pairwise Spearmanʼs correlation tests were performed using the rcorr function from the Hmisc package, between the abundances of the 176 species that showed differences in the HC versus PD comparison and the clinical variables. Once correlation tables were generated for each study group, the tables for the PD and GBA-NMC groups were merged as well as the tables for the HC and PD groups. For each merged table, a new variable was created to indicate whether the correlation values between each species and each clinical variable were in the same direction (positive or negative) between the two groups compared.

Other R packages were used for various tasks: to read data files in Excel format using the functions read_excel from the readxl package (version 1.4.3)^75^ and read.xlsx from the xlsx package (version 0.6.5)^76^; to manipulate variable names with the str_rename function from the stringr package (version 1.5.1)^77^; to process data using the function foldchange from the gtools package (version 3.9.5)^78^ and dataframe format with the reshape2 package (version 1.4.4)^79^; and to create composite figures comprising multiple plots with the grid.arrange function from the gridExtra package (version 2.3)^80^.

The complete DNA extraction procedure, shotgun metagenomic sequencing, read quality control and bioinformatical preprocessing of the data are described in 10.17504/protocols.io.5qpvo9ox7v4o/v1.

### Reporting summary

Further information on research design is available in the Nature Portfolio Reporting Summary linked to this article.

## Online content

Any methods, additional references, Nature Portfolio reporting summaries, source data, extended data, supplementary information, acknowledgements, peer review information; details of author contributions and competing interests; and statements of data and code availability are available at 10.1038/s41591-026-04318-5.

## Supplementary information

Supplementary Information Pages 2−3: Appendix A. Overview of clinical features of patients with PD, carriers of *GBA1* variants (GBA-PD) or non-carriers (idiopathic PD). Pages 4−5: Appendix B. References of clinical scales. Pages 6–8: Supplementary Fig. 1: Heatmaps for Spearmanʼs correlations of differentially abundant species and clinical parameters.

Reporting Summary

Peer Review File

Supplementary Table Spreadsheet-based data.

## Figures and Tables

**Extended Data Table 1:**
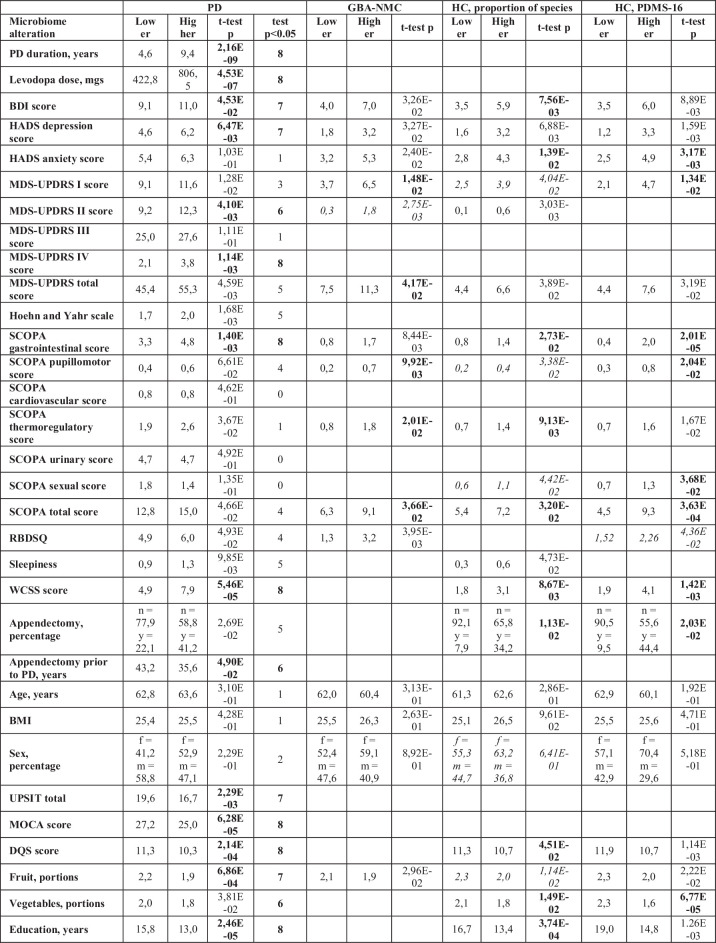
Association of clinical variables and microbiome alterations

PD patients. Comparison of top (n = 68) and bottom (n = 68) quartiles; the values shown were obtained with the abundance of coherent depleted species, the number of statistically significant tests (enriched and depleted coherent and non-coherent species, by abundance and proportion) is shown. Variables significant in 6 or more tests are highlighted in bold.

GBA-NMC individuals. Comparison of individuals below and above the median; the significant values were obtained using the proportion of coherent enriched/depleted species, bold print indicates significant values by both tests.

HC individuals, proportion of species. Comparison of individuals in the top (n = 38) and bottom (n = 38) quartiles; the values shown were obtained with the proportion of coherent PD-enriched species. In bold are the variables that were also significant with the proportion of coherent PD-depleted species, significant difference of the UPDRS part I score was found in the latter but not the former comparison (in italics).

HC individuals, PDMS-16. Comparison of individuals having PDMS-16 of −5 or less (lower microbiome alteration, n = 21) with those having PDMS-16 of 3 or more (higher microbiome alteration, n = 27); significantly different parameters in comparison of the latter individuals with the remainder of the HC cohort (n = 123) are indicated by p-values in bold print, significant difference of the RBDSQ score was found in the latter but not the former comparison (in italics).

p values were computed by the one-sided Student t-test for continuous variables and by the two-sided Chi2 test for categorical variables.

n, no; y, yes; f, female; m, male; BMI, body mass index (m/kg^2^); BDI, Beck Depression Inventory; DQS, Dietary Quality Score; HADS, Hospital Anxiety and Depression Scale; UPDRS, Unified Parkinson’s Disease Rating Scale; MOCA, Montreal Cognitive Assessment; PD, Parkinson’s disease; SCOPA, SCales for Outcomes in PArkinson’s disease; RBDSQ, REM Sleep Behavior Disorder Questionnaire; UPSIT, University of Pennsylvania Smell Identification Test; WCSS, Wexner Constipation Scoring System.

## Data Availability

The data, code, protocols and key laboratory materials used and generated in this study are listed in a Key Resource Table alongside their persistent identifiers at the Software Heritage URL: https://archive.softwareheritage.org/browse/origin/directory/?origin_url=https://github.com/metagenopolis/ASAP_human; GitHub URL: https://github.com/metagenopolis/ASAP_human. The clinical data used in the preparation of this article are available through the UCL Research Data Repository at the following link: 10.5522/04/30710741. The metagenomics sequencing data that support the findings of this study are available through the Aligning Science Across Parkinson’s Collaborative Research Network Cloud (ASAP CRN Cloud) (RRID: SCR_023923): ‘Human fecal shotgun metagenomic sequencing in Parkinson’s disease individuals, non-manifesting *GBA1* variant carriers and healthy controls’ (10.5281/zenodo.18353680). The data are controlled. Researchers can register for access to these data by submitting a Data Use Application through the ASAP CRN Cloud website (https://cloud.parkinsonsroadmap.org/collections). Data dictionaries, README files, protocols used to collect the data and data processing pipelines are openly available at https://cloud.parkinsonsroadmap.org/collections.

## Code availability

The code used to generate the results of this article can be found at the Software Heritage URL: https://archive.softwareheritage.org/browse/origin/directory/?origin_url=https://github.com/metagenopolis/ASAP_human; GitHub URL: https://github.com/metagenopolis/ASAP_human.
