# Supplementary material for: Microbiome signature of Parkinson’s disease in healthy and genetically at-risk individuals
Source: Nat Med. 2026 Apr 20;32(6):2096–106. doi: 10.1038/s41591-026-04318-5 (PMC13279262; doi:10.1038/s41591-026-04318-5)
Supplement: Supplementary file 1 — Pages 2−3: Appendix A. Overview of clinical features of patients with PD, carriers of GBA1 variants (GBA-PD) or non-carriers (idiopathic PD). Pages 4−5: Appendix B. References of clinical scales. Pages 6–8: Supplementary Fig. 1: Heatmaps for Spearmanʼs correlations of differentially abundant species and clinical parameters. [file 41591_2026_4318_MOESM1_ESM.pdf]

nature medicine

Supplementary information https://doi.org/10.1038/s41591-026-04318-5

Microbiome signature of Parkinson's disease in healthy and genetically at-risk individuals

In the format provided by the authors and unedited

Supplementary Information

Microbiome signature of Parkinson's disease in healthy and genetically at-risk individuals

Table of Content:

1) **Page 2-3: Appendix A. Overview of clinical features of PD patients, carriers of *GBA1* variants (GBA-PD) or non-carriers (idiopathic PD).**
2) **Page 4-5: Appendix B. References of clinical scales.**
3) **Page 6-8: Supplementary Fig. 1: Heatmaps for Spearman correlations of differentially abundant species and clinical parameters.**

Appendix A. Overview of clinical features of PD patients, carriers of *GBA1* variants (GBA-PD) or non-carriers (idiopathic PD).

	GBA-PD (N=128)	Idiopathic PD (N=186)	*p values*
**DEMOGRAPHICS**			
**GBR cohort (N)**	63	92	-
**ITA cohort (N)**	65	94	-
**Age**	61 ± 9	65 ± 9	0.0002
**Sex (% F, N)**	48% (61)	42% (78)	ns
**Positive PD Family History (%, N)**	37% (47)	23% (43)	0.0127
**Education (yrs)**	14.3 ± 4.1	13.9 ± 4.4	ns
**Participation with partner (%, N)**	28% (36)	25% (47)	ns
**Participation with family member (%, N)**	14% (18)	8% (14)	ns
**BMI (kg/m^2)**	25.6 ± 4.5	25.8 ± 5.1	ns
**DISEASE-ASSOCIATED FEATURES**			
**PD Age at onset**	54 ± 10	59 ± 9	<0.0001
**PD Duration**	6.8 ± 5	6.1 ± 5.1	ns
**Drug naïve (%, N)**	6% (8)	15% (28)	0.0260
**LEDD (mg)**	646.3 ± 479.5	552.4 ± 430.7	ns
**DBS (%, N)**	20% (25)	8% (15)	0.0047
**Apomorphine pump infusion (%, N)**	0.8% (1)	0	ns
**LCIG (%, N)**	0.8% (1)	0	ns
**CLINICAL FEATURES**			
**MDS-UPDRS part I**	10.9 ± 6.2	9.9 ± 6.9	ns
**MDS-UPDRS part II**	11.5 ± 6.1	10.4 ± 7.0	0.0273
**MDS-UPDRS part III**	28 ± 13.1	27 ± 12.8	ns
**MDS-UPDRS part IV**	3.8 ± 3.7	2.6 ± 3.4	**0.0026**
**MDS-UPDRS total**	53.9 ± 20.7	49.4 ± 22.9	0.0321
**H&Y stage (median)**	2	2	ns
**SCOPA-AUT total**	14.3 ± 7.5	13.6 ± 7.5	ns
**SCOPA Gastrointestinal**	4.1 ± 2.8	3.8 ± 2.9	ns
**SCOPA Urinary**	4.8 ± 2.9	5.1 ± 3.1	ns
**SCOPA Cardiovascular**	0.9 ± 1.1	0.7 ± 1.0	0.0343
**SCOPA Thermoregulatory**	2.6 ± 2.4	2.0 ± 1.9	ns
**SCOPA Pupillomotor**	0.5 ± 0.9	0.5 ± 0.7	ns
**SCOPA Sexual**	1.4 ± 1.8	1.5 ± 1.8	ns
**WCSS**	6.1 ± 4.6	5.7 ± 4.4	ns
**RBDSQ (score)***	5.7 ± 3.6	5.0 ± 3.6	ns
**RBDSQ above cut-off (%, N)**	44% (55)	36% (67)	ns
**UPSIT**	17.4 ± 5.6	18.9 ± 6.7	**0.0020**
**HADS anxiety**	6.4 ± 3.6	5.3 ± 3.9	**0.0033**
**HADS depression**	6.1 ± 3.9	5.0 ± 3.5	**0.0081**
**BDI**	11.4 ± 6.5	8.6 ± 6.5	**<0.0001**
**MOCA (score)***	25.7 ± 4.1	25.7 ± 3.5	ns
**MOCA below cut-off (%, N)**	36% (46)	38% (71)	ns

BMI, body mass index; BDI, Beck Depression Inventory; DBS, deep brain stimulation; DQS, Dietary Quality Score; HADS, Hospital Anxiety and Depression Scale; H&Y, Hoehn & Yahr; LCIG, levodopa-carbidopa intestinal gel; LEDD, levodopa equivalent daily dose; GBR, Great Britain; ITA, Italy; MDS-UPDRS, Movement Disorder Society (MDS) Unified Parkinson's Disease Rating Scale; MOCA, Montreal Cognitive Assessment; PD, Parkinson disease; SCOPA-AUT, SCales for Outcomes in PArkinson's disease; RBDSQ, REM Sleep Behavior Disorder Questionnaire; UPSIT, University of Pennsylvania Smell Identification Test; WCSS, Wexner Constipation Scoring System. P values are reported as numerical values or as ns (not significant). P values (two-sided t-test or non-parametric Wilcoxon rank-sum) related to clinical features which resisted to adjustment for multiple comparisons are indicated in bold. *: for RBDSQ and MOCA, total scores have been reported in Appendix A for completeness and p values of total scores' comparisons have been also reported although they have not been included in the adjustment for multiple testing (as p values from binary regression models for RBDSQ and MOCA have been included).

Appendix B. References of clinical scales.

Clinical scale full name	Clinical scale abbreviation	Reference
Movement Disorder Society-Unified Parkinson's Disease Rating Scale (part I-IV)	MDS-UPDRS	Goetz, C. G. et al. Movement Disorder Society-sponsored revision of the Unified Parkinson's Disease Rating Scale (MDS-UPDRS): scale presentation and clinimetric testing results. Mov Disord 23, 2129-2170 (2008). https://doi.org/10.1002/mds.22340
Hoehn & Yahr	H&Y	Hoehn, M. M. & Yahr, M. D. Parkinsonism: onset, progression and mortality. Neurology 17, 427-442 (1967). https://doi.org/10.1212/wnl.17.5.427
SCales for Outcomes in PArkinson's disease	SCOPA-AUT	Visser, M., Marinus, J., Stiggelbout, A. M. & Van Hilten, J. J. Assessment of autonomic dysfunction in Parkinson's disease: the SCOPA-AUT. Mov Disord 19, 1306-1312 (2004). https://doi.org/10.1002/mds.20153
Wexner Constipation Scoring System	WCSS	Agachan, F., Chen, T., Pfeifer, J., Reissman, P. & Wexner, S. D. A constipation scoring system to simplify evaluation and management of constipated patients. Dis Colon Rectum 39, 681-685 (1996). https://doi.org/10.1007/BF02056950
REM Sleep Behaviour Disorder Questionnaire	RBDSQ	Stiasny-Kolster, K. et al. The REM sleep behavior disorder screening questionnaire--a new diagnostic instrument. Mov Disord 22, 2386-2393 (2007). https://doi.org/10.1002/mds.21740
University of Pennsylvania Smell Identification Test	UPSIT	Doty, R. L., Shaman, P. & Dann, M. Development of the University of Pennsylvania Smell Identification Test: a standardized microencapsulated test of olfactory function. Physiol Behav 32, 489-502 (1984). https://doi.org/10.1016/0031-9384(84)90269-5
Hospital Anxiety and Depression Scale	HADS	Zigmond, A. S. & Snaith, R. P. The hospital anxiety and depression scale. Acta Psychiatr Scand 67, 361-370 (1983). https://doi.org/10.1111/j.1600-0447.1983.tb09716.x
Beck Depression Inventory	BDI	Beck, A. T., Epstein, N., Brown, G. & Steer, R. A. An inventory for measuring clinical anxiety: psychometric properties. J Consult Clin Psychol 56, 893-897 (1988). https://doi.org/10.1037//0022-006x.56.6.893
Montreal Cognitive Assessment (cognitive impairment defined as MOCA score <26/30; normal	MOCA	Nasreddine, Z. S. et al. The Montreal Cognitive Assessment, MoCA: a brief screening tool for mild cognitive impairment. J Am Geriatr Soc 53, 695-699 (2005). https://doi.org/10.1111/j.1532-5415.2005.53221.x<br><br>Lawson, R. A. et al. Stability of mild cognitive impairment in newly diagnosed Parkinson's disease. J

cognition defined as MOCA score ≥26/30)		Neurol Neurosurg Psychiatry 88, 648-652 (2017). https://doi.org/10.1136/jnnp-2016-315099<br><br>Rossetti, H. C., Lacritz, L. H., Cullum, C. M. & Weiner, M. F. Normative data for the Montreal Cognitive Assessment (MoCA) in a population-based sample. Neurology 77, 1272-1275 (2011). https://doi.org/10.1212/WNL.0b013e318230208a

A

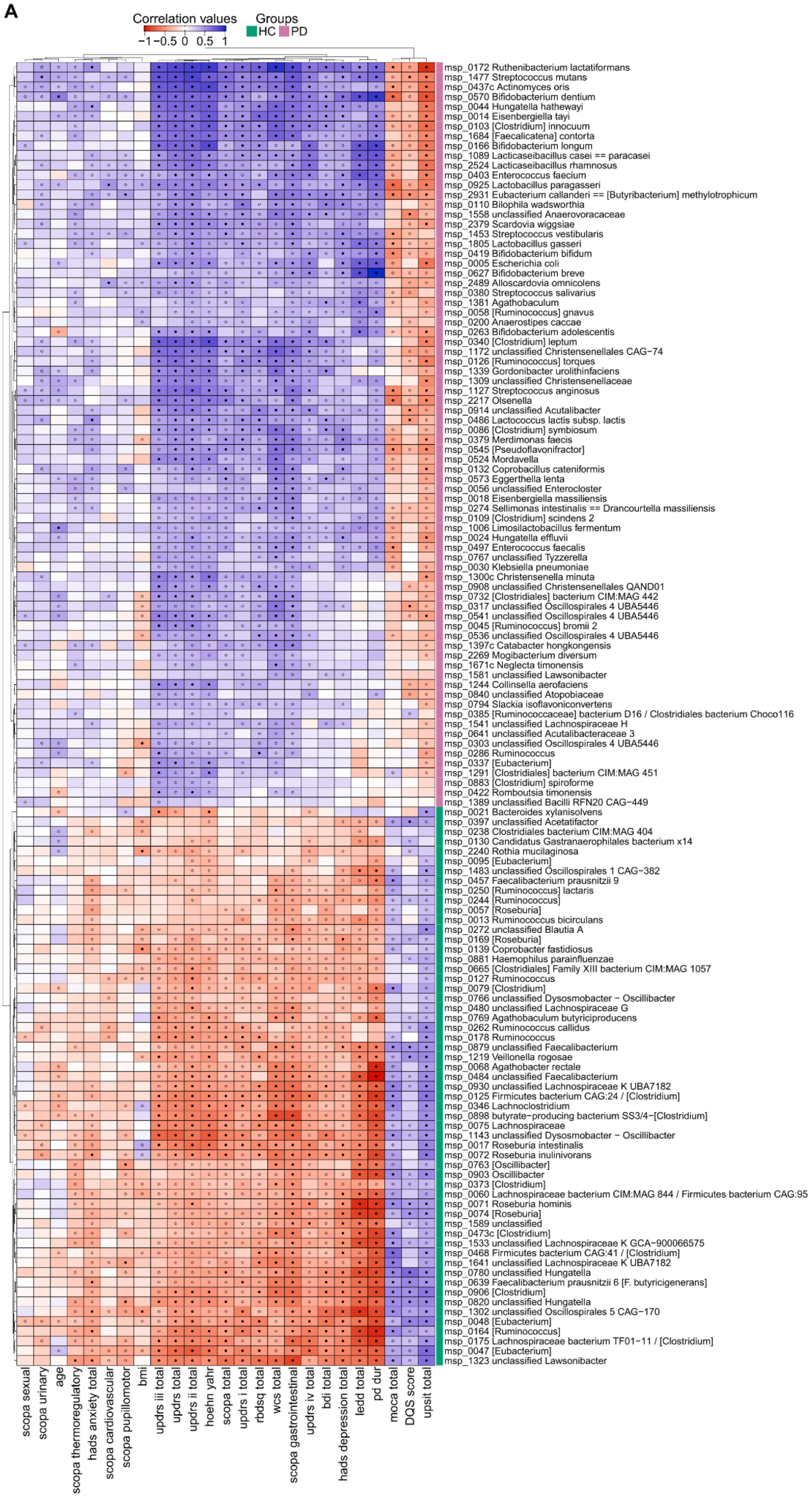

Correlation values
−1 −0.5 0 0.5 1
Groups
HC
PD
msp_0172 Ruthenibacterium lactatiformans
msp_1477 Streptococcus mutans
msp_0437c Actinomyces oris
msp_0570 Bifidobacterium dentium
msp_0044 Hungatella hathewayi
msp_0014 Eisenbergiella tayi
msp_0103 [Clostridium] innocuum
msp_1684 [Faecalicatena] contorta
msp_0166 Bifidobacterium longum
msp_1089 Lacticaseibacillus casei == paracasei
msp_2524 Lacticaseibacillus rhamnosus
msp_0403 Enterococcus faecium
msp_0925 Lactobacillus paragasseri
msp_2931 Eubacterium callanderi == [Butyribacterium] methylotrophicum
msp_0110 Bilophila wadsworthia
msp_1558 unclassified Anaerovoracaceae
msp_2379 Scardovia wiggsiae
msp_1453 Streptococcus vestibularis
msp_1805 Lactobacillus gasseri
msp_0419 Bifidobacterium bifidum
msp_0005 Escherichia coli
msp_0627 Bifidobacterium breve
msp_2489 Alloscardovia omnicolens
msp_0380 Streptococcus salivarius
msp_1381 Agathobaculum
msp_0058 [Ruminococcus] gnavus
msp_0200 Anaerostipes caccae
msp_0263 Bifidobacterium adolescentis
msp_0340 [Clostridium] leptum
msp_1172 unclassified Christensenellales CAG−74
msp_0126 [Ruminococcus] torques
msp_1339 Gordonibacter urolithinfaciens
msp_1309 unclassified Christensenellaceae
msp_1127 Streptococcus anginosus
msp_2217 Olsenella
msp_0914 unclassified Acutalibacter
msp_0486 Lactococcus lactis subsp. lactis
msp_0086 [Clostridium] symbiosum
msp_0379 Merdimonas faecis
msp_0545 [Pseudoflavonifractor]
msp_0524 Mordavella
msp_0132 Coprobacillus cateniformis
msp_0573 Eggerthella lenta
msp_0056 unclassified Enterocloster
msp_0018 Eisenbergiella massiliensis
msp_0274 Sellimonas intestinalis == Drancourtella massiliensis
msp_0109 [Clostridium] scindens 2
msp_1006 Limosilactobacillus fermentum
msp_0024 Hungatella effluvii
msp_0497 Enterococcus faecalis
msp_0767 unclassified Tyzzerella
msp_0030 Klebsiella pneumoniae
msp_1300c Christensenella minuta
msp_0908 unclassified Christensenellales QAND01
msp_0732 [Clostridiales] bacterium CIM:MAG 442
msp_0317 unclassified Oscillospirales 4 UBA5446
msp_0541 unclassified Oscillospirales 4 UBA5446
msp_0045 [Ruminococcus] bromii 2
msp_0536 unclassified Oscillospirales 4 UBA5446
msp_1397c Catabacter hongkongensis
msp_2269 Mogibacterium diversum
msp_1671c Neglecta timonensis
msp_1581 unclassified Lawsonibacter
msp_1244 Collinsella aerofaciens
msp_0840 unclassified Atopobiaceae
msp_0794 Slackia isoflavoniconvertens
msp_0385 [Ruminococcaceae] bacterium D16 / Clostridiales bacterium Choco116
msp_1541 unclassified Lachnospiraceae H
msp_0641 unclassified Acutalibacteraceae 3
msp_0303 unclassified Oscillospirales 4 UBA5446
msp_0286 Ruminococcus
msp_0337 [Eubacterium]
msp_1291 [Clostridiales] bacterium CIM:MAG 451
msp_0883 [Clostridium] spiroforme
msp_0422 Romboutsia timonensis
msp_1389 unclassified Bacilli RFN20 CAG−449
msp_0021 Bacteroides xylanisolvens
msp_0397 unclassified Acetatifactor
msp_0238 Clostridiales bacterium CIM:MAG 404
msp_0130 Candidatus Gastranaerophilales bacterium x14
msp_2240 Rothia mucilaginosa
msp_0095 [Eubacterium]
msp_1483 unclassified Oscillospirales 1 CAG−382
msp_0457 Faecalibacterium prausnitzii 9
msp_0250 [Ruminococcus] lactaris
msp_0244 [Ruminococcus]
msp_0057 [Roseburia]
msp_0013 Ruminococcus bicirculans
msp_0272 unclassified Blautia A
msp_0169 [Roseburia]
msp_0139 Coprobacter fastidiosus
msp_0881 Haemophilus parainfluenzae
msp_0665 [Clostridiales] Family XIII bacterium CIM:MAG 1057
msp_0127 Ruminococcus
msp_0079 [Clostridium]
msp_0766 unclassified Dysosmobacter − Oscillibacter
msp_0480 unclassified Lachnospiraceae G
msp_0769 Agathobaculum butyriciproducens
msp_0262 Ruminococcus callidus
msp_0178 Ruminococcus
msp_0879 unclassified Faecalibacterium
msp_1219 Veillonella rogosae
msp_0068 Agathobacter rectale
msp_0484 unclassified Faecalibacterium
msp_0930 unclassified Lachnospiraceae K UBA7182
msp_0125 Firmicutes bacterium CAG:24 / [Clostridium]
msp_0346 Lachnoclostridium
msp_0898 butyrate−producing bacterium SS3/4−[Clostridium]
msp_0075 Lachnospiraceae
msp_1143 unclassified Dysosmobacter − Oscillibacter
msp_0017 Roseburia intestinalis
msp_0072 Roseburia inulinivorans
msp_0763 [Oscillibacter]
msp_0903 Oscillibacter
msp_0373 [Clostridium]
msp_0060 Lachnospiraceae bacterium CIM:MAG 844 / Firmicutes bacterium CAG:95
msp_0071 Roseburia hominis
msp_0074 [Roseburia]
msp_1589 unclassified
msp_0473c [Clostridium]
msp_1533 unclassified Lachnospiraceae K GCA−900066575
msp_0468 Firmicutes bacterium CAG:41 / [Clostridium]
msp_1641 unclassified Lachnospiraceae K UBA7182
msp_0780 unclassified Hungatella
msp_0639 Faecalibacterium prausnitzii 6 [F. butyricigenerans]
msp_0906 [Clostridium]
msp_0820 unclassified Hungatella
msp_1302 unclassified Oscillospirales 5 CAG−170
msp_0048 [Eubacterium]
msp_0164 [Ruminococcus]
msp_0175 Lachnospiraceae bacterium TF01−11 / [Clostridium]
msp_0047 [Eubacterium]
msp_1323 unclassified Lawsonibacter
scopa sexual
scopa urinary
age
scopa thermoregulatory
hads anxiety total
scopa cardiovascular
scopa pupillomotor
bmi
updrs iii total
updrs total
updrs ii total
hoehn yahr
scopa total
updrs i total
rbdsq total
wcs total
scopa gastrointestinal
updrs iv total
bdi total
hads depression total
ledd total
pd dur
moca total
DQS score
upsit total

B

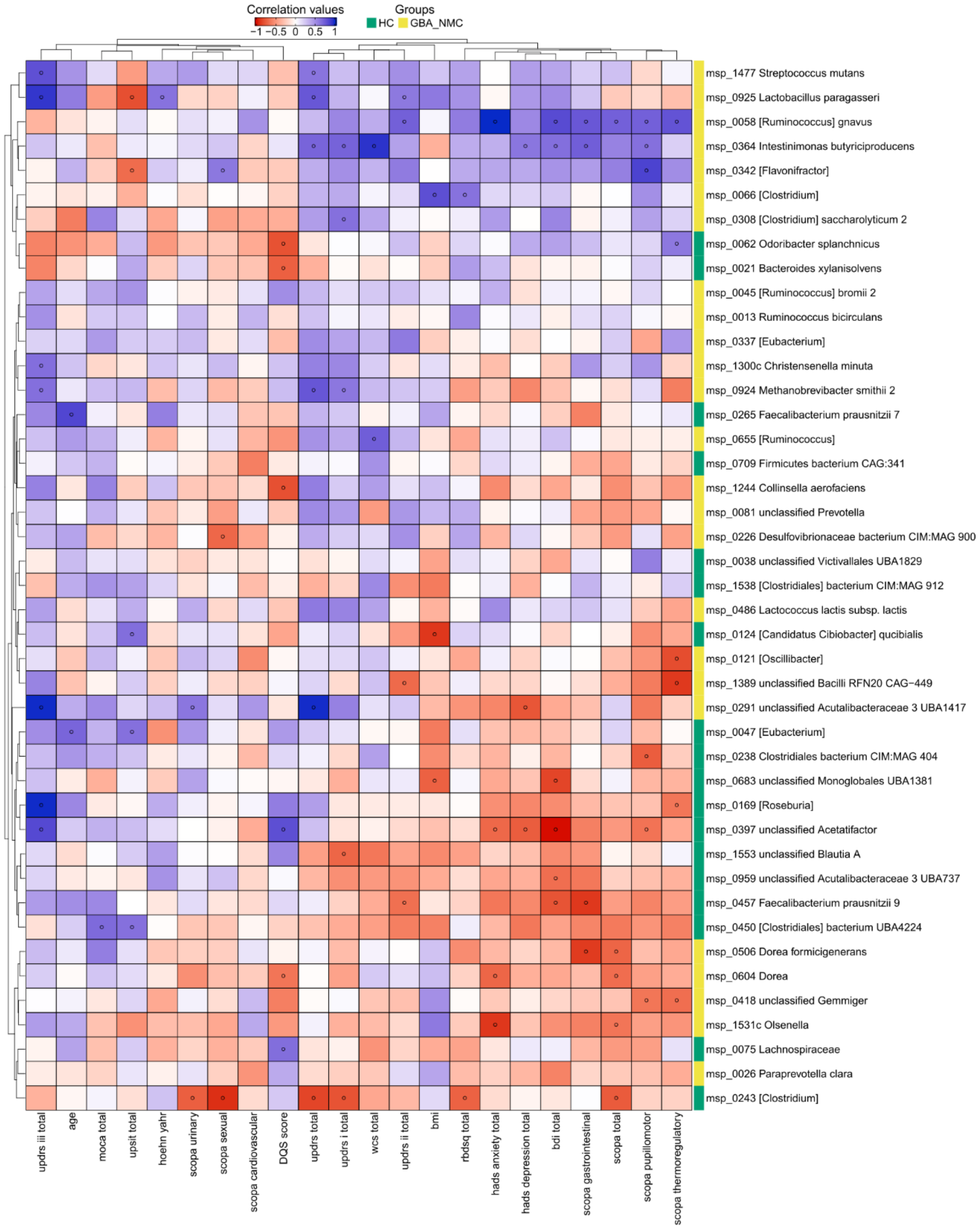

Supplementary Fig. 1: **Heatmaps for Spearman correlations of differentially abundant species and clinical parameters.** A. Correlation heatmap for the 133 differentially abundant MSP species enriched in HC (n=150, green) and PD (n=271, mauve) participants, according to q<0.1. B. Correlation heatmap for the 43 differentially abundant MSP species enriched in HC

(green) and GBA-NMC (n=43, yellow) participants. Positive correlations are indicated in blue, negative correlations in red. Solid and open dots referred to significant Spearman correlations of $p<0.001$ and $p<0.05$, respectively. P-values of the correlation analyses between microbial species and clinical parameters within PD and GBA-NMC groups are displayed in Supplementary Tables 8 and 9.
