# Supplementary material for: Microbiome signature of Parkinson’s disease in healthy and genetically at-risk individuals
Source: Nat Med. 2026 Apr 20;32(6):2096–106. doi: 10.1038/s41591-026-04318-5 (PMC13279262; doi:10.1038/s41591-026-04318-5)
Supplement: Supplementary file 2 — Reporting Summary [file 41591_2026_4318_MOESM2_ESM.pdf]

nature portfolio

Corresponding author(s): Stanislav Dusko Ehrlich, Victoria Meslier, Anthony HV Schapira

Last updated by author(s): Mar 2, 2026

Reporting Summary

Nature Portfolio wishes to improve the reproducibility of the work that we publish. This form provides structure for consistency and transparency in reporting. For further information on Nature Portfolio policies, see our Editorial Policies and the Editorial Policy Checklist.

Statistics

For all statistical analyses, confirm that the following items are present in the figure legend, table legend, main text, or Methods section.

n/a	Confirmed	
☐	☒	The exact sample size (*n*) for each experimental group/condition, given as a discrete number and unit of measurement
☐	☒	A statement on whether measurements were taken from distinct samples or whether the same sample was measured repeatedly
☐	☒	The statistical test(s) used AND whether they are one- or two-sided<br>*Only common tests should be described solely by name; describe more complex techniques in the Methods section.*
☐	☒	A description of all covariates tested
☐	☒	A description of any assumptions or corrections, such as tests of normality and adjustment for multiple comparisons
☐	☒	A full description of the statistical parameters including central tendency (e.g. means) or other basic estimates (e.g. regression coefficient) AND variation (e.g. standard deviation) or associated estimates of uncertainty (e.g. confidence intervals)
☐	☒	For null hypothesis testing, the test statistic (e.g. F, t, r) with confidence intervals, effect sizes, degrees of freedom and P value noted<br>*Give P values as exact values whenever suitable.*
☒	☐	For Bayesian analysis, information on the choice of priors and Markov chain Monte Carlo settings
☐	☒	For hierarchical and complex designs, identification of the appropriate level for tests and full reporting of outcomes
☐	☒	Estimates of effect sizes (e.g. Cohen's *d*, Pearson's *r*), indicating how they were calculated

Our web collection on statistics for biologists contains articles on many of the points above.

Software and code

Policy information about availability of computer code

Data collection	REDCap software was used for clinical data collection. No software was used for microbiome data collection.
Data analysis	R program was used for data analysis; the code used is available at: https://archive.softwareheritage.org/browse/origin/directory/?origin_url=https://github.com/metagenopolis/ASAP_human; GitHub URL: https://github.com/metagenopolis/ASAP_human.

For manuscripts utilizing custom algorithms or software that are central to the research but not yet described in published literature, software must be made available to editors and reviewers. We strongly encourage code deposition in a community repository (e.g. GitHub). See the Nature Portfolio guidelines for submitting code & software for further information.

Data

Policy information about availability of data

All manuscripts must include a data availability statement. This statement should provide the following information, where applicable:

- Accession codes, unique identifiers, or web links for publicly available datasets
- A description of any restrictions on data availability
- For clinical datasets or third party data, please ensure that the statement adheres to our policy

The data, code, protocols, and key lab materials used and generated in this study are listed in a Key Resource Table alongside their persistent identifiers at the Software Heritage URL: https://archive.softwareheritage.org/browse/origin/directory/?origin_url=https://github.com/metagenopolis/ASAP_human; GitHub URL: https://github.com/metagenopolis/ASAP_human.

The clinical data used in the preparation of this article are available through the UCL Research Data Repository at the following link: https://doi.org/10.5522/04/30710741.

The metagenomics sequencing data that support the findings of this study are available through the Aligning Science Across Parkinson's Collaborative Research Network Cloud (ASAP CRN Cloud) (RRID:SCR_023923): "Human fecal shotgun metagenomic sequencing in Parkinson's disease individuals, non-manifesting GBA1 variant carriers and healthy controls"; DOI: 10.5281/zenodo.18353680. The data are controlled. Researchers can register for access to these data by submitting a Data Use Application through the ASAP CRN Cloud website (https://cloud.parkinsonsroadmap.org/collections). Data dictionaries, README files, protocols used to collect the data, and data processing pipelines are openly available at https://cloud.parkinsonsroadmap.org/collections.

Research involving human participants, their data, or biological material

Policy information about studies with human participants or human data. See also policy information about sex, gender (identity/presentation), and sexual orientation and race, ethnicity and racism.

Reporting on sex and gender	Sex was defined based on self-report.
Reporting on race, ethnicity, or other socially relevant groupings	This information is not present in the dataset.
Population characteristics	Age- ans sex-matched individuals with Parkinson's disease, healthy controls and non-manifesting GBA1 carriers
Recruitment	For the UK cohort, recruitment was conducted through the RAPSODI study.<br>For the ITALIAN participants, recruitment was conducted through IRCCS Mondino Foundation-Pavia, and Neurology Unit, Neuromotor & Rehabilitation Department, Azienda USL-IRCCS-Reggio Emilia.
Ethics oversight	London – Queen Square REC: 15/LO/1155; EC of Pavia: code P-20210009687; EC of Area Vasta Emilia Nord: code 2021/0092531

Note that full information on the approval of the study protocol must also be provided in the manuscript.

Field-specific reporting

Please select the one below that is the best fit for your research. If you are not sure, read the appropriate sections before making your selection.

☒ Life sciences ☐ Behavioural & social sciences ☐ Ecological, evolutionary & environmental sciences

For a reference copy of the document with all sections, see nature.com/documents/nr-reporting-summary-flat.pdf

Life sciences study design

All studies must disclose on these points even when the disclosure is negative.

Sample size	A 77.6% decrease in the composition of bacterial families (specifically Prevotellaceae) is reported in Parkinson's disease patients compared to control subjects. Considering an effect size d=0.77, standard deviation of each group of 1.01, significance level 0.05, power 0.8 and Bonferroni correction for multiple comparison, the sample size needed to detect a significantly difference between the three groups was estimated to be N=54 subjects per group.
Data exclusions	Samples that did not pass QC were excluded from the analysis.
Replication	The study results have been replicated using data from 3 independent cohorts from the United States (PD, n=491; HC=234), from Turkey (PD, n=69; HC=17) and from Korea (PD, n=78; HC=68).
Randomization	Not applicable: this was a cross-sectional study, not a randomised study. No intervention was performed on subjects, and therefore no random allocation into groups.
Blinding	Not applicable: this was a cross-sectional study, not a randomised study. The investigators were not blinded during data collection nor data analysis.

Reporting for specific materials, systems and methods

We require information from authors about some types of materials, experimental systems and methods used in many studies. Here, indicate whether each material, system or method listed is relevant to your study. If you are not sure if a list item applies to your research, read the appropriate section before selecting a response.

Materials & experimental systems

n/a	Involved in the study
☒	☐ Antibodies
☒	☐ Eukaryotic cell lines
☒	☐ Palaeontology and archaeology
☒	☐ Animals and other organisms
☐	☒ Clinical data
☒	☐ Dual use research of concern
☒	☐ Plants

Methods

n/a	Involved in the study
☒	☐ ChIP-seq
☒	☐ Flow cytometry
☒	☐ MRI-based neuroimaging

Clinical data

Policy information about clinical studies

All manuscripts should comply with the ICMJE guidelines for publication of clinical research and a completed CONSORT checklist must be included with all submissions.

Clinical trial registration: This study is not a clinical trial, so there is no registration number.

Study protocol: This study is not a clinical trial, so there is no trial protocol associated. The study protocol for the clinical studies have been approved by the specific REC.

Data collection: Data were collected from the 3 study centers (UCL London, Mondino Pavia, Reggio Emilia), between 2021 and 2024.

Outcomes: This study is not a clinical trial, so there are no primary and secondary outcomes associated. Clinical features suggestive of Parkinson's disease and microbiome features were compared between GBA1 non-manifesting carriers and either healthy subjects or Parkinson's disease patients.

Plants

Seed stocks: *Report on the source of all seed stocks or other plant material used. If applicable, state the seed stock centre and catalogue number. If plant specimens were collected from the field, describe the collection location, date and sampling procedures.*

Novel plant genotypes: *Describe the methods by which all novel plant genotypes were produced. This includes those generated by transgenic approaches, gene editing, chemical/radiation-based mutagenesis and hybridization. For transgenic lines, describe the transformation method, the number of independent lines analyzed and the generation upon which experiments were performed. For gene-edited lines, describe the editor used, the endogenous sequence targeted for editing, the targeting guide RNA sequence (if applicable) and how the editor was applied.*

Authentication: *Describe any authentication procedures for each seed stock used or novel genotype generated. Describe any experiments used to assess the effect of a mutation and, where applicable, how potential secondary effects (e.g. second site T-DNA insertions, mosiacism, off-target gene editing) were examined.*
