# Supplementary material for: Microbiome signature of Parkinson’s disease in healthy and genetically at-risk individuals
Source: Nat Med. 2026 Apr 20;32(6):2096–106. doi: 10.1038/s41591-026-04318-5 (PMC13279262; doi:10.1038/s41591-026-04318-5)
Supplement: Supplementary file 3 — Peer Review File [file 41591_2026_4318_MOESM3_ESM.pdf]

nature portfolio

# Peer Review File

# Microbiome signature of Parkinson’s disease in healthy and genetically at-risk individuals

Corresponding Author: Professor Anthony Schapira

**This file contains all reviewer reports in order by version, followed by all author rebuttals in order by version.**

Version 0:

Reviewer comments:

Reviewer #1

(Remarks to the Author)
A. Summary: GBA1 mutation carriers are at increased risk for Parkinson’s disease. In this paper, Menozzi et al. report that the PD-relevant alterations in microbiome composition of GBA1 carriers is intermediate between PD and controls. They identify patterns that start in controls, gain strength in GBA1-carriers and come to full bloom in overt PD. This is a timely and important topic. The paper is well-written and succinct. Reads easily. But when you try to understand what exactly is being done, there are prohibitive gaps in description of methods and inconsistencies in approach, as listed in C, D.

B. Originality: Authors point out innovative methodology. Indeed, interesting paths were taken in data analysis, but descriptions are insufficient to evaluate them. There is not much on GBA/PD/microbiome in the literature. This study can help fill an important gap.

C,D. Data, methods, statistics: Study includes 109 PD with GBA1 mutation,162 PD w/out GBA1, 43 controls with GBA1, and 150 controls w/out GBA, with fecal metagenome, and rich clinical data.

First, they compared differential abundances (DA) of species in the various groups. The issue of significance/power is a concern, not only because we are interested in knowing which species are associated in which groups, but also because the “significant” findings from DA analysis are used to define “coherent” species, which is the interesting part of the paper. In -omics association studies significance is often set at multiple testing corrected $q<0.05$. Here, they used $q<0.1$ in the first DA test and abandoned it thereafter. Comparing PD w/GBA vs. PD w/o GBA, they state no species survived multiple testing correction ($q<0.1$) and concluded “in overt PD, GBA1 genetic status has little or no impact on the microbiome composition” and they proceeded to pool them. But comparing DA in controls w/GBA vs. control w/o GBA did not produce anything reaching $q<0.1$ (Table S3), yet in text they report finding 43 species with significantly different abundances. The issue of significance needs to be dealt with, a threshold decided, stated at the start, and adhered to consistently.

Second, they used the “significant” results of DA tests, to categorize species into “coherently enriched” or “coherently depleted” and made observations based on “coherence” to place GBA1-carrier controls intermediate to PD and non-carrier controls. I assumed that coherent means concordant. I am using my imagination; there is little explanation on what is being defined and how. For example, if two species have a positive direction in two datasets, are they counted as “coherently enriched”, irrespective of the strength of associations and statistical significance? On face value, I find the conclusions of coherent analyses important and interesting, as it seems to map the appearance and progression of microbial alterations from healthy control to control w/GBA to PD. But I need more detail to understand what is being done.

Third, they stratified subjects into quartiles of “most and least altered microbiome”, or divided them at median, and compared clinical phenotypes. It is not clear what is being divided in four, or two. What measure is being used to quantify “altered microbiome”? Next, they use another unconventional rule: “When p values were significant in 3 comparisons or less, we considered them to be only weakly or even not correlated with microbiome alterations…(if significant) in at least 6 different comparisons we suggest that these parameters were highly correlated with microbiome alterations”. How did they decide on 3 and 6 and their ties to significance? Later, they state “Interestingly and importantly, comparison of clinical variables of HC individuals assigned to the extreme quartiles, ….(Supplementary Table 7), revealed significant differences of most variables.”, but nothing in Supplementary Table 7 exceeds 3, which was decided not to be significant. The rules should be applied consistently. The clinical data are very rich. Ability to assign prodromal phenotype is cool. There is a struggle with small sample sizes (especially of GBA1-carrier controls) dwindling further with stratification. Could a more streamlined study design overcome the hurdles of power?

Fourth, they create a new metric, PDMS-16, to predict which healthy controls are closer to developing PD. I have concerns about PDMS-16. First, they chose 16 species with largest absolute difference in abundance in PD vs. control. Species that are less common and therefore have a smaller absolute difference, e.g. pathogens, were excluded, while they may have more substantial ties to the origin and progression of PD than say common fiber degraders that made the list. Then, "summing the number of PD-enriched species and deducing the number of PD-depleted species that each individual harbored we computed a score, termed PD Microbiome Score-16 (PDMS-16)." As written, they have now switched from relative abundance to presence/absence of a species in an individual. This is hard to evaluate as all the foundation work done to this point is on relative abundance. Moreover, not every depleted species weighs as much and therefore cancels out just any enriched species. Lacking one PD-associated butyrate producer does not cancel out having a pathogen, the former could be compensated by other butyrate producers whereas the latter could be a triggering cause of disease in of itself.

E. Conclusions. Discussion is reasonable. Conclusions made in various sections of results are reasonable. The concluding statement of abstract is not supported by this or any data. It is not known if nutrition can mitigate enrichment of harmful species, there is no evidence that mitigating microbiome alterations could modify disease progression, and no evidence that microbiome alteration can decrease risk of disease development. I am concerned that results and conclusions may be affected by ascertainment bias. 51% of GBA carriers had a positive family history of PD, far greater than familial component of PD itself. Moreover, 41% GBA carriers were genetic relatives. Skewed ascertainment toward positive family history of PD inadvertently selects for familial risk for PD, and inclusion of genetically related individuals further enriches the familial/genetic predisposition to PD in the cohort. The partial similarity to PD microbiome in this GBA cohort might therefore be partly, or entirely, due to PD component in the GBA1 families. Is there a way to evaluate or correct for this ascertainment bias?

F. Suggested improvements. Included in B-E.

G. References: I suggest adding couple of references on DA analyses of PD vs controls and make the point that despite the concerns over power and significance, it is reassuring that the signals they see recapitulate the previously published associations.

H. Clarity and context. Abstract, introduction and discussion are clear. Results are easy to read but a challenge to evaluate because methods are not explained sufficiently.

I. Supplementary tables should be made consistent (some show q value, some don't; some tests have full results in supplement, some don't). Please add data dictionary for headings and legends with explanations.

J. Data should be made publicly available. Privacy of subjects can be protected by anonymizing the data before posting.

(Remarks on code availability)

Reviewer #2

(Remarks to the Author)
This is an impressively creative and ambitious manuscript with compelling results. The authors compare people who are undiagnosed with PD but who have GBA1 mutations with both traditional PD and healthy controls, as this group is at high risk for developing PD and may also serve as a proxy for prodromal PD. They use a somewhat non-traditional approach to not only compare differences in specific microbial features, but they also explore differences in the overall microbial trends in a manner designed to be translatable to a clinical setting. While some parts of the manuscript are not novel (comparisons of PD vs healthy controls), they effectively ground the other comparisons which are novel. Significant care has gone into the formatting of the manuscript, making it generally very readable. Due to the complexity of the analysis, some changes are still required to improve interpretability, but overall the authors should be commended for their attention to detail.

With that in mind, there are several key issues with the statistical approach which will need to be addressed before publication. These generally include the statistical tool used for differential abundance analysis, the overreliance on t tests throughout the manuscript, and the limited/absent use of p value correction and covariable correction. The format of some figures and presentation of some data will also need modification, and some conclusions are not fully justified. While these constitute major revisions, I believe that there is a good chance that the core of the manuscript will be conserved after implementing these changes, as currently the results match the literature quite well and the effect sizes are strong. The paper as a whole is of interest to the field, and I would like to see the revised story published. My hope is that the authors are encouraged by my feedback. I have tried to be detailed in each of my comments in order to clarify the expectations for revisions.

Please find minor and major comments below:

Minor:

1. The term 'Parkinson disease' should be replaced with 'Parkinson's disease' throughout the manuscript.

2. Line 51 - the meaning of the phrase "we show that a large component of the gut microbiome evolves from healthy across

GBA-NMC towards PD" is a bit unclear - please reword.

3. Line 72 - there are also non-genetic causes of PD, such as pesticide exposure, that don't seem to require gene mutations. This sentence is therefore slightly misleading, and it would be a good idea to rephrase this sentence a little.

4. Line 125: 'estimated probability' - is this the estimated prior probability of PD?

5. Line 149: "interestingly, the former two include oral species." Many families are found in both locations, so I would make the connection to your later results (line 180) a bit clearer or remove this line.

6. Please include the full name of each species the first time it is introduced.

7. I found the section describing Fig 1 a little hard to follow, as there are many moving parts. The authors should consider rewording this section to make it a little clearer what is being compared, as the results are interesting and this will make it more impactful. From my understanding, they first compared HC with GBA-NMC and PD separately, then looked at the 20 species that were perfectly coherent, then expanded to evaluate all 176 species that were altered in PD (but not necessarily GBA-NMC), where the 142 with coherent effect sizes included the 20 perfectly coherent species. They then looked at the species whose effect sizes were not coherent with PD, and found that the effect sizes were similar to the HC group.

8. Use of clinical data: you mention at one point in the manuscript that when comparing 8 groups of microbial quantiles, you considered variables associated with 6+ groups to be relevant. The cutoffs used later on when assessing 4 groups should also be transparent (ex. supplementary table 7).

9. Lines 287, 292: Please define 'identical'.

10. The plots in general are very nicely arranged; however, I suggest using a colorblind palette since the colours are critical to many of the plots. http://www.cookbook-r.com/Graphs/Colors_(ggplot2)/#a-colorblind-friendly-palette

11. Supplementary data: Type (CD, CE, NE, ND) should be defined.

12. Line 306: what is the biological rationale for restricting the microbes to those that correlate positively with disease duration?

13. Not all of the reported methods seem to be relevant to the manuscript - for example, line 721 references the eggNOG and TIGRFAM databases, which don't seem to be mentioned elsewhere. The MOMR package has been removed from CRAN, and so the link provided at line 693 is broken. The link to the GitHub repo is also nonfunctional (maybe the repo is set to private?). I believe Nature requires code availability statements, which is currently missing. Please review the methods and remove/update things as needed.

Major:

1. Line 108/Table 1: according to Table 1, there are several significant differences between GBA-NMC and the other two groups which seems to go against the manuscript text. Similarly, Line 355, Fig 4d-f: the manuscript reports that similar trends are seen in the non-PD groups, but the results appear to be significantly more random, especially in the GBA group (very few negative green bars in GBA, somewhat opposite trends in the MOCA etc in the HC group, etc). This should be reflected in the text.

2. Table 1: Since the carriers and non-carriers in the PD group are compared in text and there is an unusually high proportion of carriers, the characteristics of these two groups should ideally be compared as well (either by expanding Table 1 or as a supplementary table if it's too cluttered). I'm curious as to whether there is a reason why the proportion of GBA1 carriers is so high in this cohort's PD group (>40% vs ~15% average), as it seems to have been tested after the participant was already recruited (i.e. this wasn't part of the study design).

3. Line 136: using the Wilcoxon rank-sum test on rarefied data is not ideal for differential abundance analysis - although the momr package was originally designed for microbiome analysis, it is relatively inaccurate due to the quirks of microbiome data distribution and has since been replaced by more sophisticated tools. Unless the authors can provide strong justification for using a deprecated tool, the authors should implement at least one of the top-performing tools that are specially designed for this data type, such as ANCOM-BC2, MaAsLin2, ALDEx2, and LinDA. This will also let the authors control for confounding variables as discussed below. Please see this paper for a general overview of contemporary differential abundance tools: https://www.nature.com/articles/s41467-022-28034-z. Similarly, it is unclear how the Chi2 test is being used to test microbial enrichment, but similar issues likely apply as microbiome data are not normal.
* Note: the Chi2 test is appropriately used in tests such as line 167 - the above refers to parts such as line 149.

4. Line 137: q<0.05 is the typical cutoff for Benjamini-Hochberg corrected data, especially if only one differential abundance tool is used. Please lower the cutoff or justify the higher cutoff used.

5. Line 141: "We conclude that, in overt PD, GBA1 genetic status has little or no impact on the microbiome composition, whereas the disease itself has a strong impact." This conclusion can't quite be drawn from the current analysis, as it's unclear how strongly the 82 and 123 microbial differences overlap. This is a valuable question, however, so I recommend

the following two additions (B at the very least, but A would be a strong addition):
- A) Run a beta diversity analysis on the three (or four, including GBA-NMC) groups. If the two PD groups overlap (P>0.05) but are separate from the controls (P < 0.05), then this supports your conclusion. This would also be a good test to include for your paper in general.
- B) Instead of running the statistics on each group separately, control for genetic status within the differential abundance tools discussed in 3).
*The authors should also control for other relevant factors such as sequencing depth (necessary for most differential abundance tools except for ANCOM-BC2), constipation/stool firmness if available, anti/probiotic use, geographical location, etc.

6. Line 187 etc: the use of proportion as a metric is a bit confusing to me. I understand that a higher proportion might indicate that the PD microbes are more relevant to a given participant, but the biological interpretation does not seem different enough from abundance to be justified - the number of PD-related species might be interesting, but proportion is arguably confounded by alpha diversity. Because the results with proportion seem to mostly mirror that of the abundance, I would actually suggest removing the proportion half of each analysis while performing revisions. This would dramatically streamline the manuscript, both for the reader and also in terms of the statistics (since testing two closely related variables is tricky due to the idea of multiple comparisons, as discussed in #8 below). If kept in, the authors should justify/better highlight the value of its inclusion.

7. Fig. 1:
- a,c) A non-parametric correlation test (ex. Wilcoxon, Kendall) should be used instead of Pearson as the data are not normally distributed. The bimodality artificially increases significance. In cases of strong bimodal separation (ex. Ext Fig 7), it might be best to run the correlation in each cluster individually to prove that the trend is truly conserved (or otherwise control for the variable that causes bimodality).
- b) It would be helpful to provide boxplots instead of means to visualize the distribution.
- d-g) Similar to the above, boxplots should be used if comparing individual species/samples, or chi square if comparing means with the existing plots.
*Please apply the above recommendations throughout all analyses as appropriate.

8. Fig 2: It's very interesting that the HC and GBA trends are nearly identical to the PD trends, just with smaller effect sizes. I assume that the magnitude of microbial differences in the PD quartiles are much larger than in the other groups (since by definition that should be true) - can this be touched upon via an analysis or in the discussion? For example, a sensitivity analysis could be to apply the same quantile cutoffs to the HC as was used in the PD group - the sample sizes will be much smaller, but you'd likely still have enough to see if the effect sizes are comparable to PD.

9. Fig. 2:
- The words 'diet quality' appear to have been mistakenly added to line 514.
- Since appendectomy is a Boolean variable, a Boolean statistical test should be used (i.e. chi2).
- The bar plot/use of 1 and 2 isn't quite appropriate with Boolean variables - please replace with something like a dot plot, or bars representing the fraction of participants who underwent appendectomies.
* Note: the above points apply to all Boolean variables, such as sex (Ex. extended table 1).

10. Some necessary P value correction is missing. It's good that the authors applied corrections to the differential abundance results, but the rest of the manuscript also contains a very large number of correlations between microbes/microbial groups/functions and clinical data. In some areas the results are used to characterize the cohort (ex. table 1) or are discussed as a group (ie. the number of significant correlations rather than focusing on individual correlations), and so using uncorrected p values is more justifiable; however, there are some areas where this is not true, leaving the analysis vulnerable to false positives. For illustration: the authors report how many species/modules correlate with at least one clinical variable, but with 25 variables, the chance that there will be at least one random p<0.05 correlation is already roughly 70% for each species/module (depending on sample size). The authors should take multiple comparisons into account and integrate some p value correction into their manuscript where appropriate.

11. Please include a discussion of the study limitations.

12. One of the main conclusions of the study is that better nutrition rescues the microbiome; while the data are encouraging, the trends do not seem strong enough to reach this conclusion. For PD, the microbiome correlates with disease duration - those in later PD stages have difficulty eating, which likely contributes to the observed trends. Otherwise, there are many lifestyle factors that may be acting as lurking variables - for example, the healthy controls may be more likely to have a PD-like diet since many of them live with one of the PD participants. Please temper the conclusions related to nutrition in order to reflect the uncertainty.

(Remarks on code availability)
The above link is invalid, and there is no code availability statement in their manuscript. I am unable to review their code.

Reviewer #3

(Remarks to the Author)
The manuscript by Menozzi and coworkers explores the potential influence or connection between mutations in GBA1, Parkinson's disease (PD), and the gut microbiome. In a large and adequately phenotype cohort, the study deeply profiles the

stool microbiome and correlates microbial species and biosynthetic pathway (ie., functions) to PD status and disease phenotypes. Overall, this is an important and timely area of research as the microbiome has been widely implicated in PD, but whether there are specific gene-microbiome interactions remains speculative and whether the microbiome can be used as a diagnostic for predicting who may develop PD is unknown. This last point is relevant to the field and to this study, due to the long prodromal phase prior to PD which may open the possibility of interventions to slow disease progression and the fact that the authors do identify microbiome signatures across the samples that may help identify individuals at risk for developing PD beyond genetic risk.

While the study finds several interesting outcomes, overall the lack of a GBA specific microbiome profile and lack of validation for many of the claims in the work limit enthusiasm. Firstly, the goal of the work, presumably, was to determine if individuals with GBA mutations show stereotypical changes in the gut microbiome—the study finds no or minor differences in the microbiome of individuals with PD regardless of GBA status. Secondly, many of the most striking differences in taxa and in some functions that distinguish PD from non-PD (HC or GBA-NMC) have been previously reported in numerous prior studies. Therefore, the proposal that the gut microbiome may explain the incomplete penetrance of GBA mutations to PD manifestation are not supported by the data generated.

It is interesting that there is a gradient of microbiome effects from HC to GBA-NMC to PD, which may be one of the key findings of this study. However, the assertion that this observation may delineate a “prodromal PD microbiome” is unlikely since the actual at-risk population in the GBA-NMC is not the entire group but some subset of this stratification. While some attempts are made to find people who may indeed have prodromal PD in the GBA-NMC group, the small number (n=4) makes this comparison unsubstantiated. A discovery-validation study design is needed to determine if the microbiome can in fact identify people who may go on the develop PD, and the specificity of this prediction needs to be strong (and validated by other studies) before it may serve as a clinical biomarker.

Finally, the authors spend significant effort to correlate microbiome features with symptoms and PD severity, where they do make some interesting observations of microbial species in HC and GBA-NMC that may predispose to disease. However, additional work is needed to determine if these signatures are indeed specific to PD or are more generally associated with lack of health. Many of the PD-related phenotypes mapped to microbiome alterations are general features of health, and thus the study needs to cross reference their findings with non-PD phenotypes (e.g., cognition, olfaction, poor diet are too broad to assign as PD-related). The notion that dysbiosis (whether in PD or otherwise) is a function of diet and can be improved by increased nutrition is generalizable and doesn’t provide actionable insights into PD.

(Remarks on code availability)

Version 1:

Reviewer comments:

Reviewer #1

(Remarks to the Author)
I gave a full review for the first submission. A major concern in the first submission was statistical significance. If not addressed properly, all other concerns are of no consequence. Unfortunately, this study fails statistical rigor. I empathize with authors on difficulty to identify and recruit GBA group, but the fact remains that study does not have sufficient sample size for the analyses presented. As a result, claims are not reliable. Starting with Clinical profile of GBA-NMC Lines 127-143: Having conducted 22 tests in table 1, none of the reported p-values are significant, hence claims in this section are unsupported. Moving next to “Significant microbiome alterations in PD. A total of 176 species were differentially abundant ($p<0.05$, Supplementary Table 1).” $P<0.05$ is unacceptable for differential abundance analysis. Supplementary Table 1 is not results of differential abundance. “Further analyses were based on the 176 species differentially abundant at $p<0.05$.” Indeed, the rest of the paper is further mining/testing of results that were probably not significant to begin with. Dicing the small dataset into smaller pieces, adding test after test with numerous variables, is bound to have high false positive rate. I liked the idea. It is truly unfortunate that sample size was so small and study design did not or could not correct.

(Remarks on code availability)

Reviewer #2

(Remarks to the Author)
The authors have done a good job revising the manuscript, but there are some remaining concerns. In my original review, my primary concerns revolved around certain aspects of the statistical analysis. While I accept many of their justifications, there are a few items that will need to be addressed before the manuscript could be considered for publishing.

I previously noted that the lack of P value correction was an issue in this manuscript. While I would argue this is appropriate in certain contexts (the demographics table, the differential results that are then aggregated and assessed as groups of features), there are multiple instances in the manuscript where it is objectively incorrect:

• Results/Discussion: differential abundance of individual microbes and features are discussed at some length, but use P values instead of corrected P values.

• Downstream analyses: To be considered significant, variables need to pass 6/8 tests (etc). However, these tests also use P values. The issue with this is that the tests are not orthogonal – as shown in the Reviewer Response, the abundance and prevalence outcomes are relatively strongly correlated. While the correlation isn't perfect, this still means that their correlations with any other variable are still much more likely to be similar, potentially making correlations artifactually appear more robust. Using this approach is therefore not a robust substitution for P value correction, although I very much like the idea of looking at the microbiome from multiple angles.

o I recommend keeping the 6/8 tests, but using corrected P values instead of raw P values. This may be slightly more strict than choosing one metric, but this is fair given the instability of such a downstream analysis, and would lend more credence to the results given that the approach is non-standard (but interesting).

o This statement in the Reviewer Response exemplifies the potential issue: "Using both the proportion and the abundance of species allows us to identify a higher number of clinical parameters which associate significantly with microbiome alterations." While this approach will increase sensitivity, it loses specificity. An increase in significance suggests that the results may be becoming less robust. The researchers seem to be working under the assumption that the microbiome will correlate with clinical features (ex. lines 233-5 of the revised manuscript).

In line with the above, abundance and proportion should also be defined in the manuscript as the way the manuscript/response is written suggests that they may have been used differently than is typical in the field. Without a definition, it is difficult to follow the related discussions (ex. 'Associations of microbial alterations and clinical variables in PD').

Supplementary Table 1 seems to be an abundance table instead of the differential abundance results – please update.

I have one very minor remaining point that does not constitute a barrier to publishing: after looking at the code, I believe I misunderstood how 'proportion' was defined in the original manuscript. Adding the definition into the manuscript would be very helpful since there are multiple ways that it can be interpreted.

(Remarks on code availability)
The code looks to be very straightforward and understandable. There are plenty of comments and the code chunks are well partitioned. There are no obvious errors. I anticipate no difficulties in replicating the findings.

Reviewer #3

(Remarks to the Author)
The manuscript has been satisfactorily revised and my comments addressed.

(Remarks on code availability)

**Open Access** This Peer Review File is licensed under a Creative Commons Attribution 4.0 International License, which permits use, sharing, adaptation, distribution and reproduction in any medium or format, as long as you give appropriate credit to the original author(s) and the source, provide a link to the Creative Commons license, and indicate if changes were made.
In cases where reviewers are anonymous, credit should be given to 'Anonymous Referee' and the source.
The images or other third party material in this Peer Review File are included in the article's Creative Commons license, unless indicated otherwise in a credit line to the material. If material is not included in the article's Creative Commons license and your intended use is not permitted by statutory regulation or exceeds the permitted use, you will need to obtain permission directly from the copyright holder.
To view a copy of this license, visit https://creativecommons.org/licenses/by/4.0/

Dear Professor Schapira,

Your Article, "Microbiome signature of Parkinson disease in healthy and genetically at-risk individuals" has now been seen by 3 referees. You will see from their comments below that while they find your work of interest, some important points are raised. We are interested in the possibility of publishing your study in Nature Medicine but would like to consider your response to these concerns in the form of a revised manuscript before we make a final decision on publication.

The editorial team echo the concerns of some of the reviewers regarding the need for these findings to be independently validated.

- We thank the editorial team and the reviewers for raising this important point and we acknowledge the need for validation. We have pursued validation using three separate cohorts from the US, Turkey and Korea and have added a new section describing this in the main manuscript ("*Validation of microbiome alterations across independent cohorts*"). These analyses have indicated that similar microbiome alterations like the ones observed by us take place in three independent cohorts of Parkinson's disease patients, with a different geographical background. No previous cohort has conducted the comprehensive and integrated clinical and metagenomic analyses presented in our manuscript. Clinical phenotypic data were available only for the Turkish cohort. The associations between clinical parameters and microbiome alterations identified in our Parkinson's disease cohort were largely confirmed in this independent cohort. However, due to insufficient phenotyping of a substantial number of healthy individuals in these and other studies, we were unable to assess the relationship between microbiome changes and clinical variables in healthy controls as we did in our study. This limitation has been acknowledged in our manuscript.

Secondly, all the referees raise concerns about the statistical approaches used and we ask that the authors address these concerns appropriately. Finally, it is a requirement that appropriately anonymized data be made available to the all the reviewers. This data is kept confidential and is necessary to evaluate the paper.

We therefore invite you to revise your manuscript taking into account all reviewer and editor comments. Please highlight all changes in the manuscript text file.

- We thank the Editor for the opportunity of revising our manuscript. We have addressed the concerns of the reviewers as outlined in the Responses below.

  We have worked closely with our Funding agency (ASAP) to provide secure access to you and reviewers and to guarantee reviewers' anonymity during the process.

  The metagenomic data is available through the ASAP CRN Cloud platform. Instructions to access the CRN Cloud have been submitted as a separate document in the Supplementary Material ("CRN Cloud - Journal Review Access"). We would like to emphasise that to apply for access, when the Editor or the reviewers navigate the CRN Cloud, they can select any collection card (they do not need to search for any specific card related to the present study). In the Intended Use Statement section of the Access Application, the Editor or the reviewers can provide the following information:
    - ASAP Team Name = Schapira
    - Principal Investigator = Schapira

As mentioned in the Instructions, details of reviewers will not be provided to the submitting researchers to guarantee reviewers' anonymity. The CRN Cloud team is available to assist reviewers at any stage of the process and can be contacted at cloud@parkinsonsroadmap.org.

Clinical data have been deposited and will be released through GP2 (Michael J. Fox Foundation). Access requests for GP2 data should be submitted via AMP PD: https://amp-pd.org. To facilitate timely review, we have made clinical data temporarily available for reviewers through the UCL Research Data Repository at the following link: https://figshare.com/s/1f82998108e487fd47e4. We hope this approach provides a straightforward and convenient solution for you and for the reviewers.

We are committed to providing a fair and constructive peer-review process. Do not hesitate to contact us if there are specific requests from the reviewers that you believe are technically impossible or unlikely to yield a meaningful outcome.

When revising your manuscript:

* Include a "Response to referees" document detailing, point-by-point, how you addressed each referee comment. If no action was taken to address a point, you must provide a compelling argument. This response will be sent back to the referees along with the revised manuscript.

* If you have not done so already please begin to revise your manuscript so that it conforms to our policy and format instructions here: https://www.nature.com/nm/submission-guidelines/aip-and-formatting Refer also to any guidelines provided in this letter.

* Include a revised version of any required reporting checklist. It will be available to referees (and, potentially, statisticians) to aid in their evaluation if the manuscript goes back for peer review. A revised checklist is essential for re-review of the paper.

Nature journals have recently announced an update to our guidance on reporting on sex and gender in research studies (see here). We strongly encourage researchers to follow the 'Sex and Gender Equity in Research – SAGER – guidelines' and to include sex and gender considerations for studies involving humans, vertebrate animals and cell lines where relevant to the topic of study (an overview can be found here). Authors should use the terms sex (biological attribute) and gender (shaped by social and cultural circumstances) carefully in order to avoid confusing both terms.

When preparing your revised manuscript, please be aware of our guidance on Sex and Gender reporting). Please note that:

1. If the research findings apply to only one sex or gender, that must be indicated in the title and/or abstract.

- The research findings did not apply to only one sex, hence we have not indicated this in the title and/or abstract.

2a. For studies involving vertebrates animal and cell lines- The Reporting Summary should include whether sex was were considered in the study design.

2b. For studies involving human research participants- The Reporting Summary should include whether sex and/or gender was considered in the study design and whether sex and/or gender of participants was determined based on self-report or assigned (and methodology used).

- Sex was considered in the study design as we aimed to recruit individuals who were sex-matched across the groups. As recommended by the SAGER guidelines, we have used the term sex as a classification of male or female based on biological distinction. We have specified this in the 'Methods – Study design' section (line 657).

3. Data should be reported disaggregated for sex and gender where this information has been collected and consent has been obtained for reporting and sharing individual-level data; disaggregated numbers for individual experiments must be provided in the source data as appropriate whereas overall numbers may be provided in the Nature Portfolio Reporting Summary.

- Our metadata file contains individual-level information including sex.

Information on the 3 points above should be included in the revised manuscript and detailed in the cover letter.

- Thank you for the above comments. The three points above have been integrated in the 'Methods – Study design' section (line 657). The metadata file has been made available using the instructions provided in our 'Data Availability Statement'.

In addition, please note that if sex- and gender-based analyses have been performed a priori, results should be reported regardless of positive or negative outcome. We discourage conducting post hoc sex- and gender-based analysis if the study design is insufficient (for example, low sample size) to enable meaningful conclusions.

If no sex- and gender-based analyses have been performed, please indicate the reasons for the lack of these analyses in the Reporting Summary.

- We have not performed sex-based analyses as our recruitment goal was to recruit homogenous and sex-matched groups of individuals.

When submitting the revised version of your manuscript, please also pay close attention to our href="https://www.nature.com/nature-research/editorial-policies/image-integrity">Digital Image Integrity Guidelines. and to the following points below:

-- that unprocessed scans are clearly labelled and match the gels and western blots presented in figures.
-- that control panels for gels and western blots are appropriately described as loading on sample processing controls
-- all images in the paper are checked for duplication of panels and for splicing of gel lanes.

- We confirm that no scans or gels or western blots were presented in this study.

Finally, please ensure that you retain unprocessed data and metadata files after publication, ideally archiving data in perpetuity, as these may be requested during the peer review and production process or after publication if any issues arise.

- We confirm that we have made unprocessed data, metadata files, sequencing data files and codes available for reviewers. A plan of making data publicly available after publication is in place as highlighted above and in our 'Data Availability Statement'.

Please use the link below to submit your revised manuscript and related files:

https://mts-nmed.nature.com/cgi-bin/main.plex?el=A3H5BQD1A2CRmM5J4A9ftdZAIVYn98WK0SXAx3ScOkRgZ

**Note:** This URL links to your confidential home page and associated information about manuscripts you may have submitted, or that you are reviewing for us. If you wish to forward this email to co-authors, please delete the link to your homepage.

We hope to receive your revised manuscript within three to four months. If you cannot send it within this time, please let us know. We will be happy to consider your revision so long as nothing similar has been accepted for publication at Nature Medicine or published elsewhere. Please note that we generally only allow one cycle of major experimental revision and we advise that you take the required time to properly respond to the editorial and reviewers comments.

Nature Medicine is committed to improving transparency in authorship. As part of our efforts in this direction, we are now requesting that all authors identified as 'corresponding author' on published papers create and link their Open Researcher and Contributor Identifier (ORCID) with their account on the Manuscript Tracking System (MTS), prior to acceptance. This applies to primary research papers only. ORCID helps the scientific community achieve unambiguous attribution of all scholarly contributions. You can create and link your ORCID from the home page of the MTS by clicking on 'Modify my Springer Nature account'. For more information please visit please visit www.springernature.com/orcid.

Please do not hesitate to contact me if you have any questions or would like to discuss these revisions further.

We look forward to seeing the revised manuscript and thank you for the opportunity to review your work.

Sincerely,

Jerome A. Staal
Senior Editor
Nature Medicine

Reviewers' Comments:

Reviewer #1 (Remarks to the Author):

A. Summary: GBA1 mutation carriers are at increased risk for Parkinson's disease. In this paper, Menozzi et al. report that the PD-relevant alterations in microbiome composition of GBA1 carriers is intermediate between PD and controls. They identify patterns that start in controls, gain strength in GBA1-carriers and come to full bloom in overt PD. This is a timely and important topic. The paper is well-written and succinct. Reads easily. But when you try to understand what exactly is being done, there are prohibitive gaps in description of methods and inconsistencies in approach, as listed in C, D.

B. Originality: Authors point out innovative methodology. Indeed, interesting paths were taken in data analysis, but descriptions are insufficient to evaluate them. There is not much on GBA/PD/microbiome in the literature. This study can help fill an important gap.

C, D. Data, methods, statistics: Study includes 109 PD with GBA1 mutation,162 PD w/out GBA1, 43 controls with GBA1, and 150 controls w/out GBA, with fecal metagenome, and rich clinical data.

First, they compared differential abundances (DA) of species in the various groups. The issue of significance/power is a concern, not only because we are interested in knowing which species are associated in which groups, but also because the "significant" findings from DA analysis are used to define "coherent" species, which is the interesting part of the paper. In -omics association studies significance is often set at multiple testing corrected q<0.05. Here, they used q<0.1 in the first DA test and abandoned it thereafter. Comparing PD w/GBA vs. PD w/o GBA, they state no species survived multiple testing correction (q<0.1) and concluded "in overt PD, GBA1 genetic status has little or no impact on the microbiome composition" and they proceeded to pool them. But comparing DA in controls w/GBA vs. control w/o GBA did not produce anything reaching q<0.1 (Table S3), yet in text they report finding 43 species with significantly different abundances. The issue of significance needs to be dealt with, a threshold decided, stated at the start, and adhered to consistently.

- We thank the reviewer for the comments about apparent inconsistency in the use of statistical thresholds, which lead us to clarify our approach and thus improve the manuscript. Basically, the use was modified according to the objective we tried to reach.

1) Our first objective was to determine whether microbiome composition in overt PD was impacted by *GBA1* variants or, whether it was impacted more by the disease itself (lines 138-150). To achieve this, we computed species abundance difference A) between PD carriers and non-carriers of the *GBA1* variants, and B) between each of the two PD groups and healthy controls. To assess the magnitude of microbiome composition, we compared the number of species remaining significantly different after multiple testing correction (Benjamini-Hochberg - BH, q). The threshold of 0.1 we chose in our initial submission, was previously used in the microbiome literature but prompted by reviewers' comments, we now report results at q<0.05. There were 50 and 83 significantly different species between PD carriers or non-carriers of *GBA1* variants and healthy controls, respectively, while there were none between patients of the two groups, at the threshold of 0.05 or even at the threshold of 0.6 (lines 141-145). We thus concluded that the disease in overt PD

impacts the microbiome much more than the *GBA1* genetic status and thus we pooled all the PD patients (carriers and non-carriers) together to gain statistical power.

Whether *GBA1* status could influence *per se* the gut microbiome composition was also a preliminary question that we investigated in our recent animal model of heterozygous mice carrying the severe L444P variant in the *GBA1* gene (https://www.frontiersin.org/journals/neuroscience/articles/10.3389/fnins.2025.1546203/full). In this study, we concluded that host *GBA1* genotype does not impact gut microbiome structure and composition in the L444P mouse. Prior to the present study, microbiome changes have not been studied in *GBA1* carriers. Of course, we acknowledge that this is an animal model with limitations in extrapolation to humans.

Overall, we thus cannot rule out that *GBA1* variants do not have some impact on microbiome composition, even if we cannot ascertain it: a significantly larger cohort may be required, with sufficient statistical power. We recognize that the statement in our original manuscript *"We conclude that, in overt PD, GBA1 genetic status has little or no impact on the microbiome composition, whereas the disease itself has a strong impact"*, goes beyond what we have shown (multiple testing correction only indicates that the differences could arise by chance for any individual species but does not show that they are only due to chance).

**Action taken:**
We changed the threshold to q<0.05 and reported this in the text in bold (lines 138-145): *We first compared microbiomes of PD patients, carriers (n=109) or non-carriers (n=162) of GBA1 variants, and found 44 Metagenomic Species Pangenomes (MSPs) out of 627 with prevalence of at least 10% in our cohort that were different in abundance at p<0.05 by Wilcoxon rank sum test. However, none remained significantly different after Benjamini-Hochberg correction for multiple testing at* ***q<0.05*** *(or even q<0.6). In contrast, the comparison of either group with healthy controls (HC, n=150) revealed numerous MSPs significant at* ***q<0.05*** *(50 and 83 for carrier and non-carrier group, respectively; the difference is likely due to the higher statistical power for the more numerous non-carrier group).*

We changed the manuscript to read (lines 148-150):

*We conclude that, in overt PD, GBA1 genetic status impacts the microbiome composition much less than the disease itself.*

2) Our second objective was to determine microbiome alterations in PD relative to HC (lines 151-167). We used an approach standard in the microbiome field (Wilcoxon rank-sum test and BH correction) to determine species with significantly altered abundance in the two groups; all the q values are listed in Supplementary Table 1, which allows the interested reader to assess the data at any desired threshold. The analysis reported in the text considers all species that reached the significance at the p threshold of 0.05. This was done to streamline the manuscript, as further analyses were based on these species, for reasons expanded in point 3, below.

**Action taken:**
We added the sentence at the end of the paragraph (lines 166-167):

*Further analyses were based on the 176 species, differentially abundant at p<0.05.*

3) Our third objective was to assess the microbiome alterations in GBA-NMC relative to HC (lines 170-214, section "An intermediate and consistent microbiome alteration in GBA-NMC"). As the reviewer rightly notes, standard Wilcoxon rank-sum test followed by BH correction revealed only the species significant at p<0.05, but no species differentially abundant at any significant q value. However, differently from the comparison between patients with and without *GBA1* variants, where we could just note that the differences are much smaller than between patients and HC, here we could complement the standard analysis with examination of the coherence of species variations in PD and GBA-NMC, relative to HC, using the Cliff's Delta size effect. The coherence test of the 21 species significant at p<0.05 both in PD and GBA-NMC relative to HC, revealed that all but one were coherent. We could then use a different statistical test, Chi2, assessing variation of many species together rather than each on its own, as the Wilcoxon rank-sum test does, and found it to be very significant (p=5.7E-3). Given the almost total coherence variation of species significant at p<0.05 in both PD and GBA-NMC groups, we tested coherence of variation species significant at p<0.05 in PD group only, abandoning the significance of the test based on individual species in the GBA-NMC group, and found it to be extremely significant (p=3.9E-16). This change of paradigm, shifting from examination of variation of individual species singly to that of the pool of species together, is the reason of the variation of the thresholds in the manuscript, that the reviewer notes and that we wish to maintain.

Second, they used the "significant" results of DA tests, to categorize species into "coherently enriched" or "coherently depleted" and made observations based on "coherence" to place GBA1-carrier controls intermediate to PD and non-carrier controls. I assumed that coherent means concordant. I am using my imagination; there is little explanation on what is being defined and how. For example, if two species have a positive direction in two datasets, are they counted as "coherently enriched", irrespective of the strength of associations and statistical significance? On face value, I find the conclusions of coherent analyses important and interesting, as it seems to map the appearance and progression of microbial alterations from healthy control to control w/GBA to PD. But I need more detail to understand what is being done.

- We thank the reviewer for acknowledgment of the interest and importance of the conclusions and we apologise for the lack of clarity the reviewer notes, which we have tried to correct in the new version of the manuscript.

  The reviewer is absolutely right in assuming that by "coherent" we mean "concordant"; the terms are related and both imply internal consistency. We defined coherence exactly as the reviewer states: if the two species have positive Cliff's Delta direction in the two datasets, they are considered coherently enriched (as shown in Supplementary Table 4). Interestingly, although the coherence is highest for the group of species with statistical significance below p=0.05, it is also found for species with less or no significant abundance difference: (i) a significant bias of coherent vs non-coherent species is found for all groups of species, even those with Wilcoxon p comprised between 0.5 and 1 and (ii) Cliff's Deltas of all species are correlated, at Spearman's Rho of 0.47, as illustrated in the Figure and Table below. This suggests that microbiome alterations in PD and individuals at genetic risk (GBA-NMC) may be even larger than what we describe in the

manuscript, where we restricted analyses to species with statistical significance at the Wilcoxon threshold of p<0.05.

| species group | species number | coherent | non-coherent | Chi2 |
|---|---|---|---|---|
| all | 627 | 432 | 195 | 2,19E-11 |
| p <0.05 | 176 | 142 | 34 | 3,93E-16 |
| p 0.05-0.1 | 41 | 29 | 12 | 7,93E-03 |
| p 0.1-0.25 | 92 | 66 | 26 | 3,04E-05 |
| p 0.25-0.5 | 119 | 79 | 40 | 3,50E-04 |
| p 0.5-1 | 200 | 117 | 83 | 1,62E-02 |

Table: Coherence bias for species with different statistical significance in HC vs PD comparison.

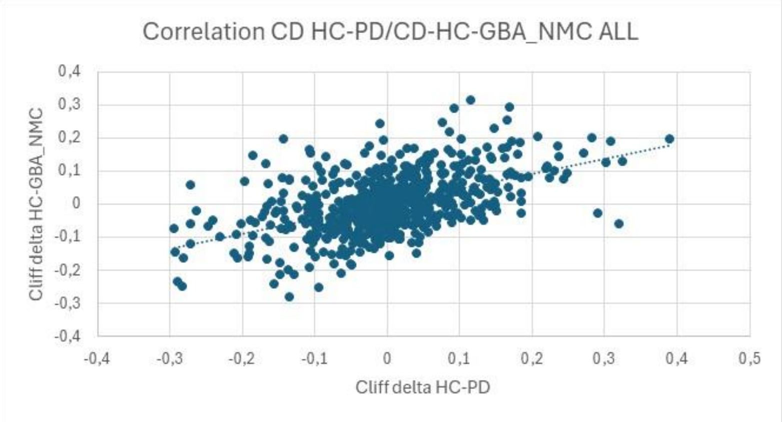


Figure: Correlation of Cliff delta for HC vs PD and HC vs GBA-NMC for all species of our cohort; each dot is a species.

**Actions taken:**

1) We inserted (bold characters) information about the way coherence is deduced (lines 174-179):

   *Of these, 21 were common with the 176 species significantly altered in PD patients compared to HC, and all but one were enriched or depleted in GBA-NMC individuals coherently with enrichment or depletion in PD patients,* ***using the direction of Cliff's delta (CD) as a guide (positive or negative, respectively),*** *which is unlikely to happen by chance (Chi$^2$ test, p=5.7E-3).*

2) We inserted (bold characters) the sentence about terming the coherent enriched and depleted species (lines 181-186).

   *Importantly, using the direction of CD, examination of the coherence of variation of the 176 MSPs significantly different between HC and PD individuals, revealed that 142 were coherently altered in GBA-NMC and PD relative to HC (Supplementary Table 4), which is very unlikely to happen by chance (Chi$^2$ test, p=3.9E-16).* ***Of these, 81 MSPs were enriched and 61 depleted in PD; we term them coherent enriched and depleted species, respectively.***

Third, they stratified subjects into quartiles of “most and least altered microbiome”, or divided them at median, and compared clinical phenotypes. It is not clear what is being divided in four, or two. What measure is being used to quantify “altered microbiome”?

- We thank the reviewer for the comment, which has helped us to improve the manuscript. For stratification in quartiles or at median, we used eight different measures of microbiome alterations: proportion or abundance of enriched or depleted species, of the coherent or non-coherent type. The eight measures are correlated but capture different aspects of the microbiome properties. For instance, an individual could have a higher proportion but lower overall abundance of coherent enriched species than another. The microbiome of the former would be more altered than that of the latter by the measure of proportion but less altered by the measure of abundance. For each of the eight measures, individuals were ordered by the corresponding portion of the microbiome they harbored, and for each partition (quartiles for PD and HC, at median for GBA-NMC) the average microbiome amounts were computed. This is shown in Extended Figure 2 (PD patients, reproduced below for convenience), and Extended Figures 4 (GBA-NMC) and 5 (HC). Details about the computational analysis can be found in the Methods section (lines 874-884).

  We acknowledge the difficulty of small sample size, in particular with the GBA-NMC group: these individuals are challenging to identify and recruit. This is why we divided the group at median, to examine all individuals in the study of intra-group microbiome heterogeneity and its relationship with clinical variables. We could not conceive of a more streamlined design to study intragroup microbiome heterogeneity in the GBA-NMC group.

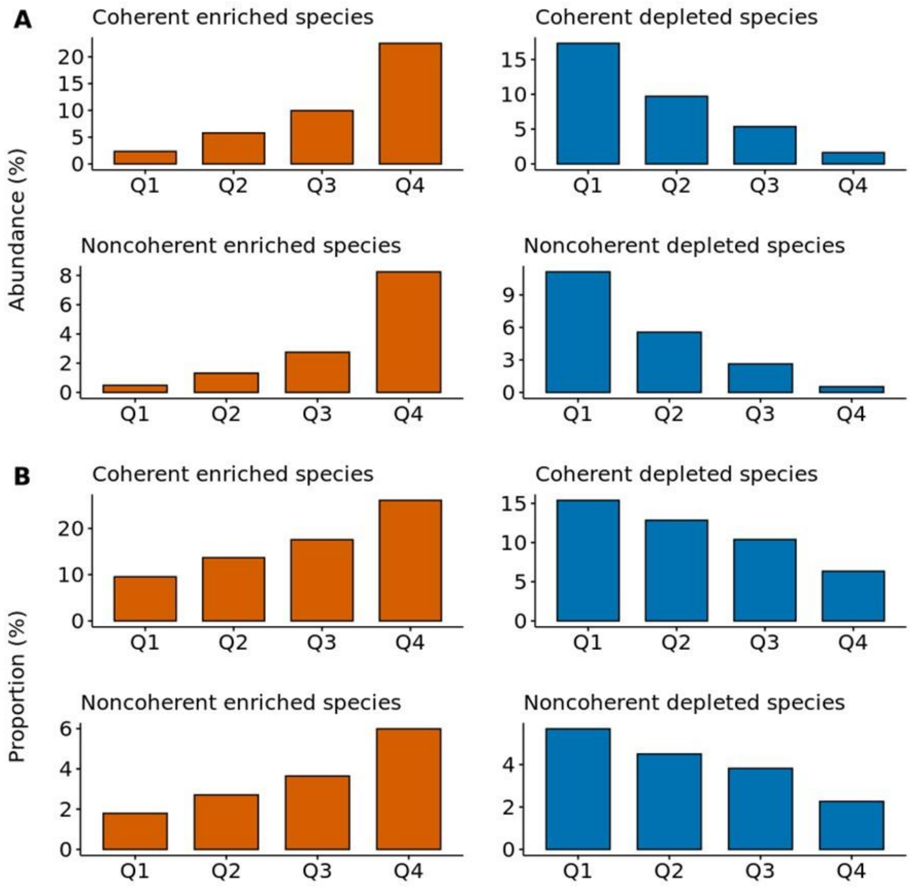


**Action taken:**
We redrafted the first paragraph of the section "Associations of microbial alterations and clinical variables in PD" (lines 217-227) as copied below for convenience:

*We estimated microbiome alterations by 8 different tests, which capture related but different aspects of microbiome composition. The first four tests were based on abundance of different species types (coherent enriched or depleted; non-coherent enriched or depleted), the other four were based on proportion of the same species types. Intuitively, an individual could have a higher proportion of coherent enriched species but a lower overall abundance of these species than another; the microbiome of the former would appear more altered than that of the latter by the test of proportion but less altered by the test of abundance. We ordered PD patients by the fraction of the microbiome estimated by each of the 8 tests and found that different quartiles vary greatly in all cases – Q1 and Q4 differed 10- to 20-fold by abundance and 2- to 3- fold by proportion (Extended Fig. 2A-D). This analysis shows considerable heterogeneity of microbiome composition among PD patients.*

Next, they use another unconventional rule: "When p values were significant in 3 comparisons or less, we considered them to be only weakly or even not correlated with microbiome alterations…(if significant) in at least 6 different comparisons we suggest that these parameters were highly correlated with microbiome alterations". How did they decide on 3 and 6 and their ties to significance?

- We thank the reviewer for this comment and apologise for the lack of clarity. Regarding the numbers of significant comparisons to investigate associations between microbiome alterations and clinical features, our intention was to differentiate the variables significantly different in most of our tests of microbiome alterations from those that are significant in very few. We clarified in the revised version of our manuscript that we considered clinical variables to be weakly or not correlated with microbiome alterations in PD patients when they were associated with microbiome alterations in less than 25% of the comparisons (so, 2 or less tests out of 8 tests). Conversely, we considered that variables were highly correlated with microbiome alterations if they were found to be significantly different in 75% or more of the 8 tests (6 or more out of 8).

  **Action taken:**
  We redrafted the text as follows (lines 228-238).

  *To investigate whether gut microbiome alterations were associated with specific clinical features in PD patients, we compared clinical variables of individuals from quartiles with highest (n=68) and lowest (n=68) microbiome alterations, estimated by each of the 8 tests. Extended Table 2 shows the results from the comparison using the abundance of coherent depleted species as test of microbiome alterations; all comparisons are displayed in Supplementary Table* 5. ***Some variables were significantly different in less than 25% comparisons (≤2/8 comparisons, e.g., age or BMI)****; we considered them to be either weakly correlated or even not correlated with microbiome alterations.* ***In contrast, certain variables were significantly different in more than 75% comparisons (≥6/8 comparisons)****, and we suggest that they are highly correlated with microbiome alterations (in bold in Extended Table 2; Fig. 2).*

Later, they state "Interestingly and importantly, comparison of clinical variables of HC individuals assigned to the extreme quartiles (Supplementary Table 7), revealed significant differences of most variables.", but nothing in Supplementary Table 7 exceeds 3, which was decided not to be significant. The rules should be applied consistently. The clinical data are very rich. Ability to assign prodromal phenotype is cool. There is a struggle with small sample sizes (especially of GBA1-carrier controls) dwindling further with stratification. Could a more streamlined study design overcome the hurdles of power?

- We thank the reviewer for the compliment on the richness of our deep-phenotyped cohort and our ability to define the prodromal status, and once again we apologise for the lack of clarity, which we have attempted to correct in the revised version of our manuscript. For GBA-NMC and non-genetically at-risk HC, we used only 4 tests to measure microbiome alterations, involving coherent species (enriched and depleted; abundance and proportion – see Supplementary Tables 6 and 7); non-coherent species, which change in abundance only in overt disease, were, expectedly, not informative. Detecting variables significantly different in comparisons involving three of four different measures of microbiome alteration is 75%, equivalent to 6/8 tests for PD. Actually, in both GBA-NMC and HC, we observed that one of the measures of alteration, based on the abundance of coherent species, was less informative than the other three. For instance, in healthy controls, the abundance of coherent enriched species, was less informative than the other three (7 significantly different variables compared to 12-15 by other three tests). We do not have an explanation for this observation: it could possibly be due to relatively high abundance variance and relatively limited group size (Q1 and Q4, n=38). That given, we suggest that

even the detection by 2 tests (proportion of enriched and depleted species), instead of 3 tests, merits highlighting, as we report in Extended Table 2.

**Action taken:**
We modified the text (lines 261-268; lines 283-285; lines 297-302) and reported the changes below:

*To investigate whether the PD microbiome signature was associated with any prodromal clinical profile in GBA-NMC, we compared clinical variables of GBA-NMC individuals with lower and higher microbiome alterations,* ***below or above the median of the abundance and proportion of coherent PD-enriched/depleted species – expectedly, noncoherent species, which change significantly in abundance only in overt disease, were not informative*** *(Extended Fig. 4; split around median rather than comparison of quartiles was used to conserve statistical power).* ***We used the threshold of 75% comparisons, also used in PD patients, to define variables highly associated with microbiome alterations****.*

*We then investigated microbiome alterations across quartiles of distribution in healthy individuals, using the* ***same four tests based on coherent species (depleted and enriched; abundance and proportion)*** *…*

*Of note is that for both GBA-NMC and HC, one of the tests, based on the abundance of coherent species, revealed fewer differences than the other three (Supplementary Tables 6 and 7). We do not have an explanation for this observation, which indicates the value of using multiple tests to assess microbiome alterations, and suggest that clinical variables significantly different by at least 2 of the other 3 tests correlate well with microbiome alterations in disease-free individuals (as reported in Extended Table 2).*

Fourth, they create a new metric, PDMS-16, to predict which healthy controls are closer to developing PD. I have concerns about PDMS-16. First, they chose 16 species with largest absolute difference in abundance in PD vs. control. Species that are less common and therefore have a smaller absolute difference, e.g. pathogens, were excluded, while they may have more substantial ties to the origin and progression of PD than say common fiber degraders that made the list. Then, "summing the number of PD-enriched species and deducing the number of PD-depleted species that each individual harbored we computed a score, termed PD Microbiome Score-16 (PDMS-16)." As written, they have now switched from relative abundance to presence/absence of a species in an individual. This is hard to evaluate as all the foundation work done to this point is on relative abundance. Moreover, not every depleted species weighs as much and therefore cancels out just any enriched species. Lacking one PD-associated butyrate producer does not cancel out having a pathogen, the former could be compensated by other butyrate producers whereas the latter could be a triggering cause of disease in of itself.

- We thank the reviewer for sharing concerns about PDMS-16. Our objective was to demonstrate that beyond overall microbiome composition, a species-based test could capture microbiome alterations associated with relevant clinical variables. We used a balance between enriched ("unhealthy") and depleted ("healthy") species for that purpose. A similar concept guided the establishment of the "TOPOSCORE" tool, related to the responsiveness of cancer patients to immunotherapy (Cell. 2024 Jun 20;187(13):3373-3389.e16. doi: 10.1016/j.cell.2024.05.029). In our cohort, PDMS-16 is well correlated in

all study groups with measures of microbiome alteration based on the fraction of the altered microbiome, in particular proportion of enriched and depleted species (Rho above 0.7), as shown in the Figure below. It is thus not surprising that it stratifies individuals into groups with significantly different clinical variables. As for its elaboration, we used presence/absence of species (difference of proportion between PD and HC), not the abundance (abundance was used to estimate correlation between species and clinical variables within a cohort). Presence/absence of species is also the foundation of measures of microbiome alterations by proportion of different species types. However, we acknowledge that different approaches to select species could be also used, resulting in a different species set that captures sufficiently well microbiome alterations associated with relevant clinical variables. We view PDMS-16 as a proof-of-concept score, a biomarker with no relation to the possible functional role in PD of the species that compose it, and no immediate clinical usefulness, but rather as a lead to elaborate a better score. Its name was meant to suggest that it is evolutive: 16 species is what we used for the proof-of-concept, but a better score, likely based on different species number, might be forthcoming when adequately phenotyped cohorts become available.

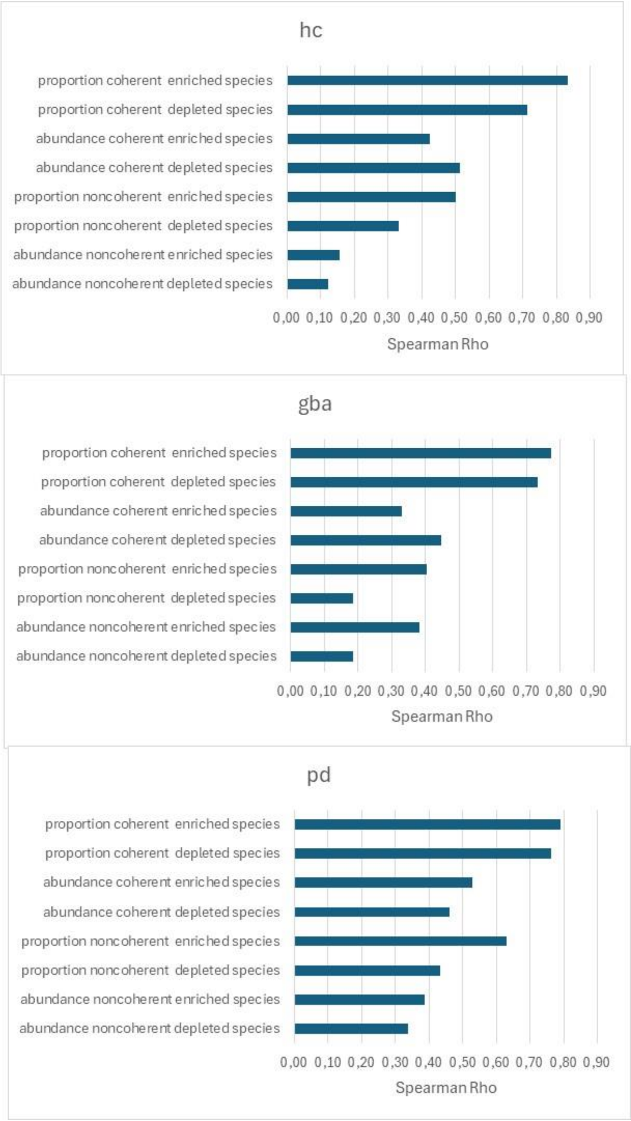


**Actions taken:**

1) We modified the relevant section (lines 340-346) to indicate the hypotheses that led to selection of species:

*To do so, we first selected the species involved in the same significant correlations with clinical variables in PD and the disease-free GBA-NMC (33 species). Among those, we then selected species with the highest prevalence difference in PD relative to HC (over 10%)-**assuming that they should be the best indicators of disease-related microbiome alterations**, and the species correlated coherently with PD duration in patients-**hypothesizing that they indicate alterations of the microbiome due to progression of disease in patients and thus possibly approaching the disease in HC**.*

2) We state that we consider PDMS-16 a proof of concept, which could lead developments of broadly applicable scores indicating risk of disease (lines 357-361):

*We conclude that the PDMS-16 score can identify individuals with a clinical profile closer to that of PD patients than the remainder of the HC group **in our study population and suggest that, as proof of concept, it could be a lead for developing a broadly applicable, species-based score of microbiome alterations indicative of PD risk in disease-free individuals, when much larger, adequately phototyped, cohorts become available**.*

3) We removed the PDMS-16 from our final statement in the Conclusion to focus more on the importance of microbiome testing in general rather than the specific use of PDMS-16 as screening test which might be restrictive at this stage (lines 491-495, in bold):

   *As these alterations appear associated with disease progression in patients, and with the development of prodromal features in individuals at-risk of PD, screening of healthy individuals **using microbiome analysis as proposed here**, may enable identification of healthy individuals at higher risk of neurodegeneration.*

E. Conclusions. Discussion is reasonable. Conclusions made in various sections of results are reasonable. The concluding statement of abstract is not supported by this or any data. It is not known if nutrition can mitigate enrichment of harmful species, there is no evidence that mitigating microbiome alterations could modify disease progression, and no evidence that microbiome alteration can decrease risk of disease development. I am concerned that results and conclusions may be affected by ascertainment bias.

- We thank the reviewer for this. Nutritional variables such as the dietary quality score (DQS) score and fruit and vegetable consumptions showed similar direction in PD patients and healthy controls, being much worse (less healthy diet, less consumption of fruit and vegetables) in individuals with worse microbiome alterations. This observation aligns with existing literature on the Mediterranean diet and other nutritional interventions, which have been shown to reduce inflammation and lower the presence of pathogenic microbes. In PD, several studies suggest that dietary interventions such as Mediterranean diet may help reduce constipation and improve cognitive function (doi: 10.3390/nu14235185; doi: 10.1016/j.cdnut.2024.102216; doi: 10.1016/j.ctim.2020.102366; doi: 10.1080/1028415X.2020.1751509). Moreover, epidemiological studies suggest that the adherence to the Mediterranean diet reduces the risk of PD (doi: 10.1016/j.jnha.2024.100451). Our data seem to align with these findings,

although we acknowledge that longitudinal studies and/or interventional studies are needed to support this.

**Action taken:**
We modified our statements in the abstract, in the Introduction and in the Discussion to mitigate the effect of nutrition or microbiome alterations on disease progression/development, and to highlight the potential utility of microbiome screening as an early marker of disease development in pre-manifest individuals.

Lines 61-63: *We conclude that gut microbiome alterations can identify both genetically and non-genetically at-risk individuals in the general population who may be progressing towards PD, thus serving as an early marker of disease development in the pre-manifest phase.*

Lines 98-105: *By comparing gut microbiomes of a cohort of PD patients (n=271), disease-free GBA1 variant carriers (n=43) and healthy controls (n=150), we find large scale PD-specific alterations in individuals of the last two groups, which are associated with a PD prodromal clinical profile. We suggest that alterations in gut microbiome composition might help explain the incomplete penetrance of PD in GBA1 variant carriers, and identify those at highest risk of conversion to PD. We further suggest that similar changes in healthy individuals without known genetic risk may also help identify those predisposed towards PD development and be used as a marker of early disease development in the pre-manifest phase.*

Lines 491-497: *As these alterations appear associated with disease progression in patients, and with the development of prodromal features in individuals at-risk of PD, screening of healthy individuals using microbiome analysis as proposed here, may enable identification of healthy individuals at higher risk of neurodegeneration. Full assessment of the power of such an approach to combat PD will have to await longitudinal follow-up of sufficiently large groups of individuals and for adequate length of time.*

51% of GBA carriers had a positive family history of PD, far greater than familial component of PD itself. Moreover, 41% GBA carriers were genetic relatives. Skewed ascertainment toward positive family history of PD inadvertently selects for familial risk for PD, and inclusion of genetically related individuals further enriches the familial/genetic predisposition to PD in the cohort. The partial similarity to PD microbiome in this GBA cohort might therefore be partly, or entirely, due to PD component in the GBA1 families. Is there a way to evaluate or correct for this ascertainment bias?

- We thank the reviewer for this comment. The extent to which host genetics can shape the gut microbiome composition is a very important point. Previous studies in twins have shown that gut microbial gene abundance profiles were more similar in monozygotic than dizygotic twins, but that environmental factors such as the age the twins started living apart significantly impacted on the observed divergence (DOI: 10.1016/j.cels.2016.10.004). Whether *GBA1* status could influence *per se* the gut microbiome composition was indeed a preliminary question that we investigated in our recent animal model of heterozygous mice carrying the severe L444P variant in the *GBA1* gene (https://www.frontiersin.org/journals/neuroscience/articles/10.3389/fnins.2025.1546203/f

ull). In this study, we concluded that host *GBA1* genotype does not impact gut microbiome structure and composition in the L444P mouse. Prior to the present study, microbiome changes have not been studied in *GBA1* variant carriers. For this reason, we believe that most of the PD-like microbiome alterations observed in our cohort of GBA-NMC are not secondary to shared host genetics with *GBA1*-PD patients but rather an early sign of prodromal PD. Moreover, our cohort of PD patients was mostly composed of non-carriers of *GBA1* variant (n=186) who do not share any genetic background with the GBA-NMC.

F. Suggested improvements. Included in B-E.

G. References: I suggest adding couple of references on DA analyses of PD vs controls and make the point that despite the concerns over power and significance, it is reassuring that the signals they see recapitulate the previously published associations.

- We thank the reviewer for the suggestion. We included references to previous studies.

  **Action taken:**
  The following references have been added:

  - Ref 19: Integrated Multi-Cohort Analysis of the Parkinson's Disease Gut Metagenome, doi/10.1002/mds.29300
  - Ref 20: Microbiota Composition and Metabolism Are Associated With Gut Function in Parkinson's Disease, doi/full/10.1002/mds.28052

  The text was modified as follows (lines 161-163):
  *Enrichment of Bifidobacterium and depletion of butyrate producers such as Faecalibacterium in PD patients have been previously reported*[19,20].

H. Clarity and context. Abstract, introduction and discussion are clear. Results are easy to read but a challenge to evaluate because methods are not explained sufficiently.

- We apologise for the lack of clarity of the methods. We have tried to improve this in our revised version of the manuscript.

  **Actions taken:**
  1) We introduced the sentence about the use of the Chi2 test to assess the significance of species enrichment in HC and PD groups (lines 856-858):

     *Enrichment of species from the same taxon (ranging from phyla to genera) among HC or PD patients was assessed by the Chi2 test under Excel, by comparing the observed number of species in the two groups with the expected number if they were equipartitioned.*

  2) We modified the paragraph describing computation of species prevalence and abundance across quartiles of HC and PD distribution (lines 874-878):

*Abundance and prevalence of cognate species in the quartiles of PD and HC samples were determined by first sorting the samples by a relevant parameter (abundance or prevalence of coherent or non-coherent, enriched or depleted species) and then computing the mean abundance and prevalence of each species type for the four quartiles and the average fold change of abundance and prevalence between the first and fourth quartiles.*

3) A similar modification was introduced for computing species prevalence and abundance above and below median in GBA-NMC samples (lines 879-882):

   *Abundance and prevalence of cognate species above and below the median in GBA-NMC samples were determined by first sorting the samples by a relevant parameter (abundance or prevalence of coherent enriched species) and then computing the mean abundance and prevalence of each species type.*

4) We defined functional annotation of the species (lines 833-838):

   *First, genes of the catalogues were annotated using KEGG107 database using Diamond[43], and further clustered into functional pathway modules according to KEGG (Kyoto Encyclopaedia of Genes and Genomes) Orthology (KO) groups, Gut Metabolic Modules (GMM)[44], and Gut Brain Modules (GBM)[45], **the latter using the annotations from the three KEGG, enggNOG and TIGRFAm datasets.***

I. Supplementary tables should be made consistent (some show q value, some don't; some tests have full results in supplement, some don't). Please add data dictionary for headings and legends with explanations.

- We thank the reviewer for the suggestions to improve the Supplementary Tables.

  **Actions taken:**

  1) We have added the missing q values in Supplementary Tables 8-12. For conciseness, we show only the statistically significant correlations in these Tables (p below 0.05).

  2) We report the results of all correlation tests in Supplementary Tables 5-9 and in the new Supplementary Tables 22 and 23 (for validation cohort).

  3) The clinical variables (abbreviations and definition) are now defined in Supplementary Table 5. We have included dictionary of variables of the Turkish cohort in the new Supplementary Table 21.

J. Data should be made publicly available. Privacy of subjects can be protected by anonymizing the data before posting.

- We thank the reviewer for their comment and suggestion. It is indeed a requirement that data are appropriately anonymized and kept confidential. We have worked closely with our Funding agency (ASAP) to provide secure access to reviewers and guarantee reviewers' anonymity during the process.

The metagenomic data is available through the ASAP CRN Cloud platform. Instructions to access the CRN Cloud have been submitted as a separate document in the Supplementary Material ("CRN Cloud - Journal Review Access"). We would like to emphasise that to apply for access, when the Editor or the reviewers navigate the CRN Cloud, they can select any collection card (they do not need to search for any specific card related to the present study). In the Intended Use Statement section of the Access Application, the Editor or the reviewers can provide the following information:

- ASAP Team Name = Schapira
- Principal Investigator = Schapira

As mentioned in the Instructions, details of reviewers will not be provided to the submitting researchers to guarantee reviewers' anonymity. The CRN Cloud team is available to assist reviewers at any stage of the process and can be contacted at cloud@parkinsonsroadmap.org.

Clinical data have been deposited and will be released through GP2 (Michael J. Fox Foundation). Access requests for GP2 data should be submitted via AMP PD: https://amp-pd.org. To facilitate timely review, we have made clinical data temporarily available for reviewers through the UCL Research Data Repository at the following link: https://figshare.com/s/1f82998108e487fd47e4. We hope this approach provides a straightforward and convenient solution for the reviewers.

Reviewer #2 (Remarks to the Author):

This is an impressively creative and ambitious manuscript with compelling results. The authors compare people who are undiagnosed with PD but who have GBA1 mutations with both traditional PD and healthy controls, as this group is at high risk for developing PD and may also serve as a proxy for prodromal PD. They use a somewhat non-traditional approach to not only compare differences in specific microbial features, but they also explore differences in the overall microbial trends in a manner designed to be translatable to a clinical setting. While some parts of the manuscript are not novel (comparisons of PD vs healthy controls), they effectively ground the other comparisons which are novel. Significant care has gone into the formatting of the manuscript, making it generally very readable. Due to the complexity of the analysis, some changes are still required to improve interpretability, but overall the authors should be commended for their attention to detail.

With that in mind, there are several key issues with the statistical approach which will need to be addressed before publication. These generally include the statistical tool used for differential abundance analysis, the overreliance on t tests throughout the manuscript, and the limited/absent use of p value correction and covariable correction. The format of some figures and presentation of some data will also need modification, and some conclusions are not fully justified. While these constitute major revisions, I believe that there is a good chance that the core of the manuscript will be conserved after implementing these changes, as currently the results match the literature quite well and the effect sizes are strong. The paper as a whole is of interest to the field, and I would like to see the revised story published. My hope is that the authors are encouraged by my feedback. I have tried to be detailed in each of my comments in order to clarify the expectations for revisions.

Please find minor and major comments below:

Minor:

1. The term ‘Parkinson disease’ should be replaced with ‘Parkinson’s disease’ throughout the manuscript.

- This has been replaced (in the Title and Running Title – line 1 and 51, in the abstract – line 53, and in the text – line 66).

2. Line 51 - the meaning of the phrase "we show that a large component of the gut microbiome evolves from healthy across GBA-NMC towards PD” is a bit unclear - please reword.

- We thank the reviewer for this suggestion. This has been rephrased with the following (lines 57-58):
  *“we show that a large component of the gut microbiome* ***in GBA-NMC is intermediate between healthy controls and PD****.”*

3. Line 72 - there are also non-genetic causes of PD, such as pesticide exposure, that don’t seem to require gene mutations. This sentence is therefore slightly misleading, and it would be a good idea to rephrase this sentence a little.

- We thank the reviewer for their comment. This sentence has been rephrased (lines 77-78):

***The background of PD is likely multifactorial, resulting from both** genetic factors, including single gene, risk gene and polygenic contributions, **and potentially non-genetic factors**.*

4. Line 125: ‘estimated probability’ - is this the estimated prior probability of PD?

- We thank the reviewer for this question. The ‘estimated probability’ is the estimated probability of PD, calculated according to the MDS prodromal criteria (Heinzel S, Berg D, Gasser T, et al. Update of the MDS research criteria for prodromal Parkinson's disease. Mov Disord 2019; 34(10): 1464-70), as reported in the Methods section (lines 720-730). In this study, the MDS diagnostic criteria for prodromal PD were applied to the GBA-NMC group using the online Prodromal PD Calculator (https://www.movementdisorders.org/MDS/Members-Only/Prodromal-PD-Calculator.htm). The estimated prior probability of PD, i.e., the age- adjusted prevalence of PD, was combined with the total likelihood ratio (LR) of prodromal PD obtained by multiplying the LRs of each marker. By doing so, a post-test probability percentage of developing PD (i.e., the ‘estimated probability’) was obtained for each participant. In this study, the following markers were used: age, sex, coffee consumption, smoking status, *GBA1* genetic status, diagnosis of type II, diabetes mellitus, physical inactivity, presence of RBD (as defined by screening questionnaire), history of excessive daytime somnolence, olfactory loss, constipation, urinary dysfunction, severe erectile dysfunction, depression, cognitive deficit, and subthreshold parkinsonism defined as MDS-UPDRS III total score above 6 (excluding postural and action tremor).

5. Line 149: "interestingly, the former two include oral species.” Many families are found in both locations, so I would make the connection to your later results (line 180) a bit clearer or remove this line.

- We thank the reviewer for this comment. We have removed this line.

6. Please include the full name of each species the first time it is introduced.

- We thank the reviewer for this comment. The full name of each species has been added in Pages 8-9.

7. I found the section describing Fig 1 a little hard to follow, as there are many moving parts. The authors should consider rewording this section to make it a little clearer what is being compared, as the results are interesting and this will make it more impactful. From my understanding, they first compared HC with GBA-NMC and PD separately, then looked at the 20 species that were perfectly coherent, then expanded to evaluate all 176 species that were altered in PD (but not necessarily GBA-NMC), where the 142 with coherent effect sizes included the 20 perfectly coherent species. They then looked at the species whose effect sizes were not coherent with PD, and found that the effect sizes were similar to the HC group.

- We thank the reviewer for indicating the complexity of the section describing Figure 1 and we apologise for the lack of clarity. The reviewer’s understanding of what we did is

absolutely correct. We have tried to clarify the section by indicating the comparisons we carried out.

**Action taken:**
We changed the text (lines 170-174) which now reads:

*We then investigated whether some of the PD-related gut microbiome alterations were detectable in the GBA-NMC group (n=43) relative to HC (n=150),* ***by comparing species abundance in the two groups. A total of 43 species were significantly different*** *(Supplementary Table 3, Extended Fig. 1B),* ***fewer than in the comparison of HC with PD****, possibly, at least in part, because of the loss of statistical power due to the low number of GBA-NMC.*

8. Use of clinical data: you mention at one point in the manuscript that when comparing 8 groups of microbial quantiles, you considered variables associated with 6+ groups to be relevant. The cutoffs used later on when assessing 4 groups should also be transparent (ex. supplementary table 7).

- We thank the reviewer for the suggestion, and we apologise for the lack of clarity. We indicate now that we use four tests to assess microbiome alterations in GBA-NMC and healthy individuals, based on coherent species (enriched and depleted; abundance and proportion); non-coherent species, which change in abundance only in overt disease, were, expectedly, not informative. We initially kept the same cut-off used in PD to define variables highly correlated to microbiome alterations (75% of comparisons), which involves three of four different measures of microbiome alteration (equivalent to 6/8 tests for PD). We observed though that for both GBA-NMC and healthy controls, one of the measures of alteration, based on the abundance of coherent species, was less informative than the other three. For instance, in healthy controls, the abundance of coherent enriched species, was less informative than the other three (7 significantly different variables compared to 12-15 by other three tests). We do not have an explanation for this observation: it could possibly be due to relatively high abundance variance and relatively limited group size (Q1 and Q4, n=38). This is why we suggest that even the detection by 2 tests (proportion of enriched and depleted species) merits to be highlighted, as we report in Extended Table 2.

**Action taken:**
We modified the text (lines 261-268; lines 283-285; lines 297-302) and reported the changes below:

*To investigate whether the PD microbiome signature was associated with any prodromal clinical profile in GBA-NMC, we compared clinical variables of GBA-NMC individuals with lower and higher microbiome alterations,* ***below or above the median of the abundance and proportion of coherent PD-enriched/depleted species – expectedly, noncoherent species, which change significantly in abundance only in overt disease, were not informative*** *(Extended Fig. 4; split around median rather than comparison of quartiles was used to conserve statistical power).* ***We used the threshold of 75% comparisons, also used in PD patients, to define variables highly associated with microbiome alterations****.*

*We then investigated microbiome alterations across quartiles of distribution in healthy individuals, using the **same four tests based on coherent species (depleted and enriched; abundance and proportion)** …*

*Of note is that for both GBA-NMC and HC, one of the tests, based on the abundance of coherent species, revealed fewer differences than the other three (Supplementary Tables 6 and 7). We do not have an explanation for this observation, which indicates the value of using multiple tests to assess microbiome alterations, and suggest that clinical variables significantly different by at least 2 of the other 3 tests correlate well with microbiome alterations in disease-free individuals (as reported in Extended Table 2).*

9. Lines 287, 292: Please define 'identical'.

- We thank the reviewer for requesting this definition; we realize that the term was misused and have replaced it now with correct description of our observations:

  Initial text: Some 15% of correlations were identical with those in PD patients (Supplementary Table 10) and an overwhelming majority of those (40/43) were coherent in both groups (Extended Fig. 7A).

  **Action taken:**
  We changed the text (lines 321-324, and lines 327-331) which now reads:

  *Some 15% of correlations were **between the same species and clinical variables as in PD** patients (Supplementary Table 10) and an overwhelming majority of those (39/42) **had the same direction** in both groups (Extended Fig. 7A).*

  *Similar correlations were found in healthy individuals (Fig. 4C, Supplementary Table 11). **The correlation bias was less clear than in the PD group, as observed in the GBA-NMC group, possibly for the same reasons**. The correlations involved 77% of the 176 species, with 24% of them being **between the same species and clinical variables as in the PD group** (Supplementary Table 12) and showing the same direction in both groups (75/77, Extended Fig. 7B).*

10. The plots in general are very nicely arranged; however, I suggest using a colorblind palette since the colours are critical to many of the plots. http://www.cookbook-r.com/Graphs/Colors_(ggplot2)/#a-colorblind-friendly-palette

- We thank the reviewer for their suggestion. We have updated our figure using a colorblind friendly palette.

11. Supplementary data: Type (CD, CE, NE, ND) should be defined.

- We thank the reviewer for this comment. A legend has been placed in the Supplementary Table 4 (Columns P, Q).

12. Line 306: what is the biological rationale for restricting the microbes to those that correlate positively with disease duration?

- We thank the reviewer for raising this point. In the construction of the proof-of-concept PDMS-16 score, we used 3 criteria to select the species which together detect microbiome alterations allowing us to stratify HC controls into a group with clinical variables closer to those of PD patients. We acknowledge that other criteria could have been used (see the response to a related question by Reviewer 1). The rationale for restricting by PD duration was that we hypothesized that species correlated with this variable might be best indicators of microbiome alterations correlated with the progression of the disease in patients and approaching overt disease in HC. We have modified the text to indicate this hypothesis.

  **Action taken:**
  We modified the text (lines 338-346, in bold) which now reads:

  *In view of similar correlations between clinical variables and microbial species in all study groups, we explored the possibility of stratifying HC individuals that may be closer to disease development by the microbial species they harbor. To do so, we first selected the species involved in the same significant correlations with clinical variables in PD and the disease-free GBA-NMC (33 species). Among those, we then selected species with the highest prevalence difference in PD relative to HC (over 10%)-assuming that they should be the best indicators of disease-related microbiome alterations, and the species correlated coherently with PD duration in patients-**hypothesizing that they indicate alterations of the microbiome due to progression of disease in patients and thus possibly approaching the disease in HC.***

13. Not all of the reported methods seem to be relevant to the manuscript - for example, line 721 references the eggNOG and TIGRFAM databases, which don't seem to be mentioned elsewhere. The MOMR package has been removed from CRAN, and so the link provided at line 693 is broken. The link to the GitHub repo is also nonfunctional (maybe the repo is set to private?). I believe Nature requires code availability statements, which is currently missing. Please review the methods and remove/update things as needed.

- We thank the reviewer for these comments and we apologise for the GitHub link not working. Concerning the method for the functional potential analysis, we used the eggNOG and TIGRFAM databases to annotate the genes detected in the individuals, as these annotations are used in addition to the KEGG annotation to build the GBM modules – Gut Brain Modules – that we used in the present study.

  **Action taken:**
  We modified the text (lines 833-838, in bold), that now reads as:

  *First, genes of the catalogues were annotated using KEGG107 database using Diamond[43], and further clustered into functional pathway modules according to KEGG (Kyoto Encyclopaedia of Genes and Genomes) Orthology (KO) groups, Gut Metabolic Modules (GMM)[44], and Gut Brain Modules (GBM)[45], **the latter using the annotations from the three KEGG, enggNOG and TIGRFAm datasets.***

- We apologize for the broken link to the momr package. We provided the correct link for the momr package in the text: https://forge.inrae.fr/metagenopolis/momr in the text (line 805).

- The link to the GiHub page to the CroCoDeEL (line 807) tool has now been made public.

- All the codes used to perform the analysis of the gut microbiome are available at the following GitHub link, which has now been made public: https://github.com/metagenopolis/ASAP_human (line 1167 in the manuscript).

Major:

1. Line 108/Table 1: according to Table 1, there are several significant differences between GBA-NMC and the other two groups which seems to go against the manuscript text.

- We thank the reviewer for raising this point. When we mentioned that no differences in demographic features were detected between GBA-NMC and either HC or PD, we meant no differences in age and sex, which are known to be potential confounders for microbiome analysis.

  **Action taken:**
  We have modified the text accordingly (lines 113-115):

  *When age and sex of GBA-NMC were compared to the other groups, no differences were detected. PD participants were significantly older than HC (p=0.006), and more frequently males (p=0.028).*

Similarly, Line 355, Fig 4d-f: the manuscript reports that similar trends are seen in the non-PD groups, but the results appear to be significantly more random, especially in the GBA group (very few negative green bars in GBA, somewhat opposite trends in the MOCA etc in the HC group, etc). This should be reflected in the text.

- We thank the reviewer for this criticism and acknowledge that the correlations in the GBA-NMC and HC groups are less significant than in the PD group.

  **Action taken:**
  We have modified the text (lines 314-331) to reflect better our observations (bold characters):

  *Importantly, we found a similar profile of correlations between species and clinical variables in GBA-NMC (Fig. 4B), with variables associated with severity being mostly positively correlated with species enriched in PD and negatively with species depleted in PD (Extended Fig. 6B); the converse was observed for variables associated with health.* ***However, the correlation bias was less clear than in the PD group (e.g., cognition or constipation – MOCA or WCS, respectively), possibly because of lower statistical power due to the smaller group size and/or lower variance of the variables in the GBA-NMC group.*** *The correlations involved 64% of the 176 species (Supplementary Table 9). Some 15% of correlations were between the same species and clinical variables as in PD patients (Supplementary Table 10) and an overwhelming majority of those (39/42) had the same direction in both groups (Extended Fig. 7A). Other correlations were different,*

*possibly due to relatively small variations of species abundances within a study group, resulting in a statistical significance in one group but not another.*
*Similar correlations were found in healthy individuals (Fig. 4C, Supplementary Table 11).* ***The correlation bias was less clear than in the PD group, as observed in the GBA-NMC group, possibly for the same reasons****. The correlations involved 77% of the 176 species, with 24% of them being between the same species and clinical variables as in the PD group (Supplementary Table 12) and showing the same direction in both groups (75/77, Extended Fig. 7B).*

2. Table 1: Since the carriers and non-carriers in the PD group are compared in text and there is an unusually high proportion of carriers, the characteristics of these two groups should ideally be compared as well (either by expanding Table 1 or as a supplementary table if it's too cluttered). I'm curious as to whether there is a reason why the proportion of GBA1 carriers is so high in this cohort's PD group (>40% vs ~15% average), as it seems to have been tested after the participant was already recruited (i.e. this wasn't part of the study design).

- We thank the reviewer for raising this point. We would like to clarify that every participant entering the study was previously tested for *GBA1* status, so *GBA1* status was known at the time of recruitment. The reasons why the number of *GBA1* variant carriers is higher than in the normal PD population is because, although our preliminary data from our animal model did not show any evidence that host *GBA1* genotype influences the gut microbiome composition (https://www.frontiersin.org/journals/neuroscience/articles/10.3389/fnins.2025.1546203/full), we wanted to recruit an approximately equal number of *GBA1* mutated PD and non-*GBA1* PD to pursue a preliminary two-group comparison also in humans and as many GBA-NMCs as possible to provide a genetically susceptible but disease-free group at the time of inclusion. Our human data demonstrated very few microbiome differences between these two genetically different groups, confirming the findings from our animal model, hence we considered the PD individuals as a whole. Extended Table 1 displaying clinical differences between idiopathic PD and *GBA*-PD patients has been added (pages 59-60).

3. Line 136: using the Wilcoxon rank-sum test on rarefied data is not ideal for differential abundance analysis - although the momr package was originally designed for microbiome analysis, it is relatively inaccurate due to the quirks of microbiome data distribution and has since been replaced by more sophisticated tools. Unless the authors can provide strong justification for using a deprecated tool, the authors should implement at least one of the top-performing tools that are specially designed for this data type, such as ANCOM-BC2, MaAsLin2, ALDEx2, and LinDA. This will also let the authors control for confounding variables as discussed below. Please see this paper for a general overview of contemporary differential abundance tools: https://www.nature.com/articles/s41467-022-28034-z.

- We thank the reviewer for drawing our attention to the possible pitfalls of differential abundance analysis and followed the suggestion to compare our results based on Wilcoxon rank sum (termed W below) with those obtained by Maaslin2 (termed M below).

Regarding statistical significance, p-values for different species obtained by the two methods were highly correlated, both in the HC vs PD and HC vs GBA-NMC comparisons, graphically illustrated below (Spearman Rho of 0.92 and 0.86, respectively).

Expectedly, p values were much lower in the first than in the second comparison, likely due to the group size difference (150 vs 271 individuals in the former, 150 vs 43 in the latter) but the correlation was almost the same. Importantly, an overwhelming majority, 95.4%, of species found significant in the HC vs PD comparison by W at a $p<0.05$ were also significant by M at the same threshold. Reciprocally, 87.9% of species significant by M were also significant by W. There is thus no indication that one of the methods may be more reliable than the other for significant differential abundance detection in our study population, in keeping with a recent study (https://doi.org/10.1093/bib/bbaf130), and we prefer to keep Wilcoxon for the current manuscript. Of note, in the HC vs GBA-NMC comparison, the values were somewhat lower, 80.2% of species significant by W were also significant by M, possibly due to the lower group size. However, our species selection for further analyses of coherent and non-coherent microbiome changes, was based on the statistical significance in the HC vs PD comparison (see also reply to Reviewer 1, partially reproduced here for convenience):

**Actions taken:**

1) We inserted (bold characters) information about the way coherence is deduced (lines 174-179):

   *Of these, 21 were common with the 176 species significantly altered in PD patients compared to HC, and all but one were enriched or depleted in GBA-NMC individuals coherently with enrichment or depletion in PD patients,* ***using the direction of Cliff's delta (CD) as a guide (positive or negative, respectively),*** *which is unlikely to happen by chance (Chi$^2$ test, p=5.7E-3).*

2) In the following paragraph (lines 181-186), we inserted (bold characters) the sentence about terming the coherent enriched and depleted species:

   *Importantly, using the direction of CD, examination of the coherence of variation of the 176 MSPs significantly different between HC and PD individuals, revealed that 142 were coherently altered in GBA-NMC and PD relative to HC (Supplementary Table 4), which is very unlikely to happen by chance (Chi$^2$ test, p=3.9E-16).* ***Of these, 81 were enriched and 61 depleted in PD; we term them coherent enriched and depleted species, respectively.***

   As our measures of microbiome alterations are based on the enrichment of species in inter-group comparisons, it should be noted that the Cliff Deltas that we use to assess the enrichment of species and the Maaslin2 coefficients are highly correlated, as illustrated below (data for all species are shown for two intergroup comparisons).

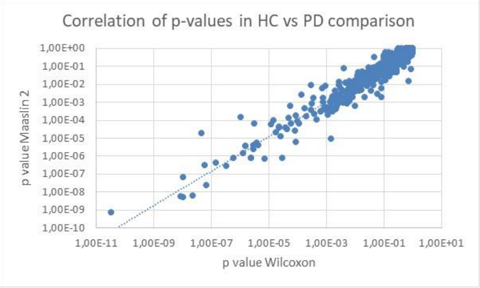


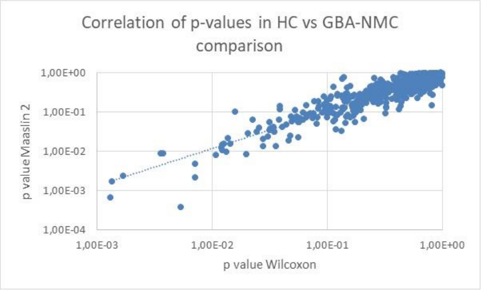

Moreover, concordance between the two was total (100%) for species differentially abundant by Wilcoxon in the HC vs PD comparison and almost complete (94.9%) for species assigned to different categories (coherent enriched, coherent depleted, noncoherent enriched and noncoherent depleted); this assignment is based on the combination of enrichment in HC vs PD and HC vs GBA-NMC comparisons. Given the high correlation of our measures and those by Maaslin 2, both in significance and enrichment, we feel that inclusion of Maaslin based analyses in our manuscript would complicate the presentation without reinforcing the message we convey.

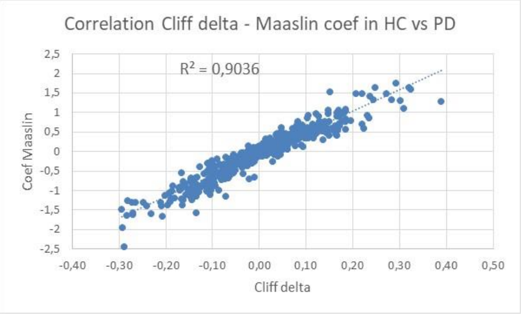


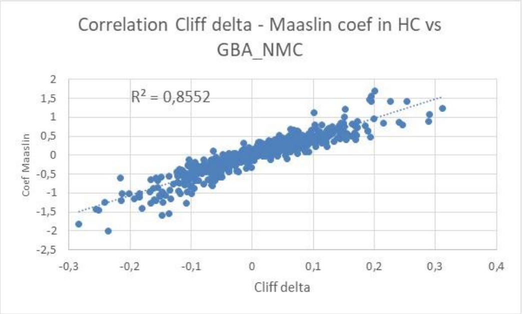


Similarly, it is unclear how the Chi2 test is being used to test microbial enrichment, but similar issues likely apply as microbiome data are not normal.
* Note: the Chi2 test is appropriately used in tests such as line 167 - the above refers to parts such as line 149.

- We apologise for the lack of clarity in the description of the use of Chi2 to test for species enrichment. We used it to estimate the probability that the species assigned to a given taxon are enriched disproportionally in the HC or the PD group. For instance, of the 16 Actinobacteriota species only 1 was enriched in the HC group whereas 15 were enriched in the PD group. The Chi2 test gives a probability of 4.65E-4 for this distribution (see Supplementary Table 2).

  **Action taken:**
  We introduced the following sentence in the Methods section (lines 856-858):

  *Enrichment of species from the same taxon (ranging from phyla to genera) among HC or PD patients was assessed by the Chi2 test under Excel, by comparing the observed number of species in the two groups with the number expected if they were equipartitioned.*

4. Line 137: q<0.05 is the typical cutoff for Benjamini-Hochberg corrected data, especially if only one differential abundance tool is used. Please lower the cutoff or justify the higher cutoff used.

- We thank the reviewer for this reminder and report numbers at q<0.05 now.

5. Line 141: "We conclude that, in overt PD, GBA1 genetic status has little or no impact on the microbiome composition, whereas the disease itself has a strong impact." This conclusion

can’t quite be drawn from the current analysis, as it’s unclear how strongly the 82 and 123 microbial differences overlap. This is a valuable question, however, so I recommend the following two additions (B at the very least, but A would be a strong addition):
- A) Run a beta diversity analysis on the three (or four, including GBA-NMC) groups. If the two PD groups overlap (P>0.05) but are separate from the controls ($P < 0.05$), then this supports your conclusion. This would also be a good test to include for your paper in general.

- B) Instead of running the statistics on each group separately, control for genetic status within the differential abundance tools discussed in 3).
*The authors should also control for other relevant factors such as sequencing depth (necessary for most differential abundance tools except for ANCOM-BC2), constipation/stool firmness if available, anti/probiotic use, geographical location, etc.

- We thank the reviewer for the valuable suggestion and carried out the microbiome beta diversity analysis by PERMANOVA, based on Bray-Curtiss distances. The graphical representation and the adonis p values are shown below.

HC vs GBA-PD: 0.001
HC vs sPD: 0.001
GBA-PD vs sPD: 0.46

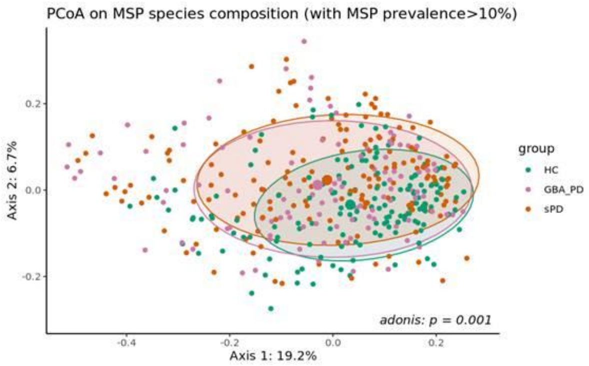


In view of such a clear result, we did not develop the other lines of investigation suggested. As outlined in the response to a comment by Reviewer 1, we agree that trying to identify possible subtle differences due to the genetic status of PD patients is of interest. However, this effort is not the main purpose of the current study, it is rather on the major microbiome alterations due to the overt disease or approaching the overt disease and would better be carried out in a separate study, which we intend to pursue.

**Action taken:**
We introduced the following sentence in the relevant paragraph (lines 145-148):

*Beta diversity analysis of Bray-Curtiss distances by PERMANOVA confirmed this conclusion, as there was no significant microbiome difference between carrier and non-carrier groups (adonis p=0.46), while the difference between HC and either group was very significant (p=0.001).*

6. Line 187 etc: the use of proportion as a metric is a bit confusing to me. I understand that a higher proportion might indicate that the PD microbes are more relevant to a given participant, but the biological interpretation does not seem different enough from abundance to be justified - the number of PD-related species might be interesting, but proportion is arguably confounded by alpha diversity. Because the results with proportion seem to mostly mirror that of the abundance, I would actually suggest removing the proportion half of each analysis while performing revisions. This would dramatically streamline the manuscript, both for the reader and also in terms of the statistics (since testing two closely related variables is

tricky due to the idea of multiple comparisons, as discussed in #8 below). If kept in, the authors should justify/better highlight the value of its inclusion.

- We thank the reviewer for raising this point. Proportion and abundance of different species types are correlated, but not perfectly, as shown in the Figure below. They capture different aspects of microbiome alteration – for instance, an individual could have higher proportion but lower overall abundance of coherent enriched species than another; the microbiome of the former would appear more altered than that of the latter by the test of proportion but less altered by the test of abundance. Composition of groups having more and less altered microbiome, which we compare throughout the manuscript, thus varies according to the test used, entailing variation of their average clinical parameters. Using both the proportion and the abundance of species allows us to identify a higher number of clinical parameters which associate significantly with microbiome alterations, in particular in the disease-free individuals, where the statistical power is lower due to a lower number of individuals and the overall variation of parameters is also lower.

  Furthermore, the abundance of a species across individuals fluctuates much more than that of the proportion – the bloom of a single species could affect measures of microbiome alteration greatly, while the loss or acquisition of a single species (or even several species) would shift the overall proportion little. This is illustrated across our study groups by the ratio of standard deviation of abundance over proportion, which is always above 1 (see the table below); a higher variance dampens significance. Specifically, in the GBA-NMC and the HC groups, one of the abundance-based tests detects fewer associations of microbiome alterations and clinical variables than the other three tests.

  As to the biological significance of abundance and proportion, one might think about the former as a bloom having occurred and the latter as a bloom waiting to occur. Abundance is a proxy of the biomass represented by a given species type at the time of sampling; proportion represents the potential of a species type to reach certain biomass in the future. It is interesting to note that the 176 species our study focuses on represent collectively a higher fraction of the microbiome by proportion than by abundance in all study groups (cf the Table below), indicating that they tend to be, on average, subdominant in the microbiome and thus have a potential to increase to dominance in some individuals.

  Given their informative value, we wish to maintain proportion-based tests in the manuscript.

| | coherent PD enriched species | coherent PD depleted species | Noncoherent PD enriched species | Noncoherent PD depleted species |
|---|---|---|---|---|
| SD of abundance/SD of proportion | | | | |
| GBA-NMC | 1,43 | 2,48 | 2,87 | 5,23 |
| HC | 1,12 | 2,15 | 1,83 | 4,04 |
| PD | 1,28 | 1,84 | 2,24 | 3,14 |

| All significant species (n=176) | mean abundance | mean proportion |
|---|---|---|
| GBA-NMC | 26,5% | 34,1% |
| HC | 24,4% | 33,7% |
| PD | 26,8% | 35,6% |

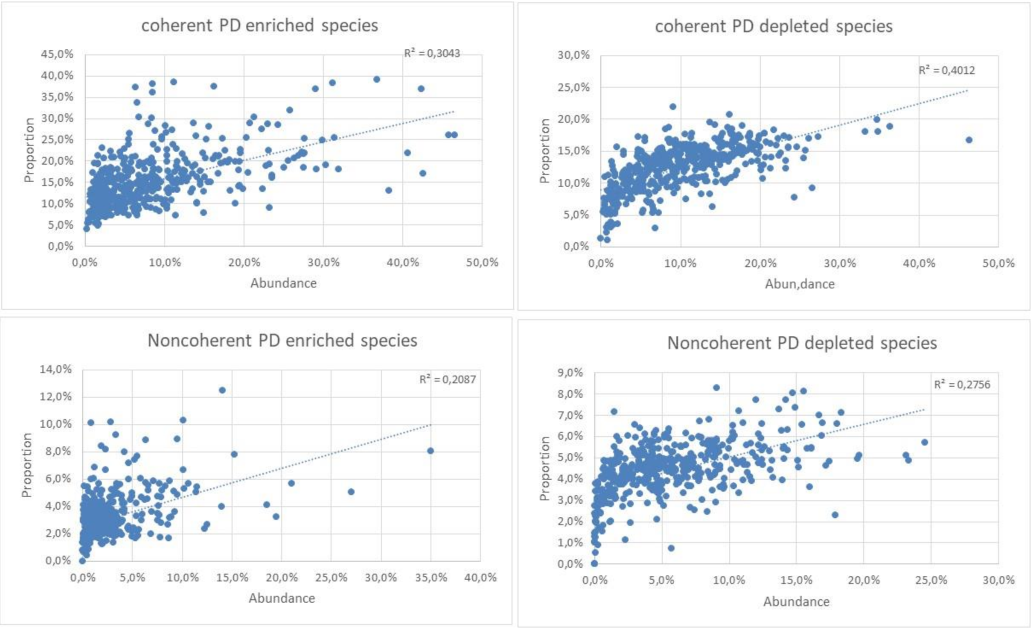


**Action taken:**
We have redrafted the first paragraph of the section "Associations of microbial alterations and clinical variables in PD" (lines 217-227), also copied below for convenience:

*We estimated microbiome alterations by 8 different tests, which capture related but different aspects of microbiome composition. The first four tests were based on abundance of different species types (coherent enriched or depleted; non-coherent enriched or depleted), the other four were based on proportion of the same species types. Intuitively, an individual could have higher proportion of coherent enriched species but lower overall abundance of these species than another; the microbiome of the former would appear more altered than that of the latter by the test of proportion but less altered by the test of abundance. We ordered patients by the fraction of the microbiome estimated by each of the 8 tests and found that different quartiles vary greatly in all cases – Q1 and Q4 differed 10- to 20-fold by abundance and 2- to 3- fold by proportion (Extended Fig. 2A-D). This analysis shows considerable heterogeneity of microbiome composition among PD patients.*

7. Fig. 1:
- a,c) A non-parametric correlation test (ex. Wilcoxon, Kendall) should be used instead of Pearson as the data are not normally distributed. The bimodality artificially increases significance. In cases of strong bimodal separation (ex. Ext Fig 7), it might be best to run the correlation in each cluster individually to prove that the trend is truly conserved (or otherwise control for the variable that causes bimodality).

- We thank the reviewer for the comment. Overall, the distribution of Cliff delta is approximately normal, as illustrated by the Figure below for the HC vs PD comparison, but we acknowledge that taking only a part of the distribution could accentuate parametric correlation.

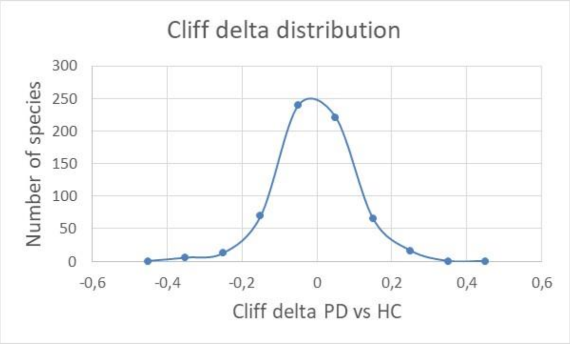


**Action taken:**
We show the non-parametric Spearman correlation values now in Figure 1.

- b) It would be helpful to provide boxplots instead of means to visualize the distribution.
- d-g) Similar to the above, boxplots should be used if comparing individual species/samples, or chi square if comparing means with the existing plots.
*Please apply the above recommendations throughout all analyses as appropriate.

- We thank the reviewer for suggesting a more informative representation of our data.

  **Action taken**
  We show the boxplots now for Fig. 1B, and 1D-G.

8. Fig 2: It's very interesting that the HC and GBA trends are nearly identical to the PD trends, just with smaller effect sizes. I assume that the magnitude of microbial differences in the PD quartiles are much larger than in the other groups (since by definition that should be true) - can this be touched upon via an analysis or in the discussion? For example, a sensitivity analysis could be to apply the same quantile cutoffs to the HC as was used in the PD group - the sample sizes will be much smaller, but you'd likely still have enough to see if the effect sizes are comparable to PD.

- We thank the reviewer for this observation. Indeed, the values of the extreme quartiles for the enriched and depleted species are higher for the former and lower for the latter, respectively, in the PD patient than in the control group, as can be seen in the Extended Figures 2 and 5, also reproduced here below for convenience (Left and Right, respectively).

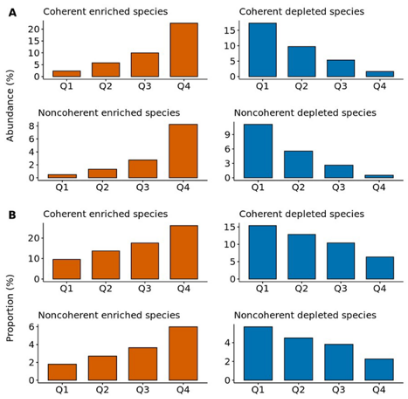


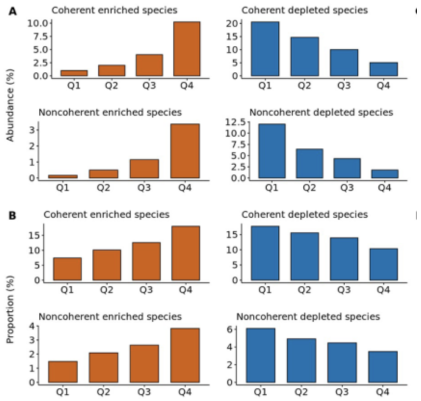

**Action taken:**
We changed the text (lines 283-287) to read:

*We then investigated microbiome alterations across quartiles of distribution in healthy individuals, using the same four tests based on coherent species (depleted and enriched; abundance and proportion) and found very significant differences, resembling those observed for PD patients,* ***albeit with lower values for the PD-enriched and higher for the PD-depleted species in the extreme quartiles (Extended Fig. 5).***

9. Fig. 2:
- The words 'diet quality' appear to have been mistakenly added to line 514.

- Diet quality refers to the variable 'diet quality score' which belong to the health-related variables shown in Panel C of Figure 2. We have added a comma in this sentence, to clarify that the encoded 1 and 2 values refer to appendectomy only, and not to diet quality (Legend of Figure 2, line 624).

- Since appendectomy is a Boolean variable, a Boolean statistical test should be used (i.e. chi2).

- We thank the reviewer for this comment. Chi2 test has been applied, and we now specify this in the Legend of Figure 2 (line 629).

  **Action taken:**
  The Legend of Panel C, Figure 2 now reads:

  *Health related variables for all groups (diet quality score, appendectomy colored in grey if individuals did not undergo appendectomy, and colored in black if people underwent appendectomy). The values of clinical variables are the means listed in Extended Table 2, standard deviations and statistical significance are indicated by the thin bars and stars indicate the significance level of the Student t test for each comparison (one-sided t-test), except for appendectomy where comparisons are tested using* ***Chi2 test.***

- The bar plot/use of 1 and 2 isn't quite appropriate with Boolean variables - please replace with something like a dot plot, or bars representing the fraction of participants who underwent appendectomies.
* Note: the above points apply to all Boolean variables, such as sex (Ex. extended table 1).

- We thank the reviewer for the remarks and introduced the requested changes.

10. Some necessary P value correction is missing. It's good that the authors applied corrections to the differential abundance results, but the rest of the manuscript also contains a very large number of correlations between microbes/microbial groups/functions and clinical data. In some areas the results are used to characterize the cohort (ex. table 1) or are discussed as a group (ie. the number of significant correlations rather than focusing on individual correlations), and so using uncorrected p values is more justifiable; however, there are some areas where this is not true, leaving the analysis vulnerable to false positives. For illustration: the authors report how many species/modules correlate with at least one clinical variable, but

with 25 variables, the chance that there will be at least one random $p<0.05$ correlation is already roughly 70% for each species/module (depending on sample size). The authors should take multiple comparisons into account and integrate some p value correction into their manuscript where appropriate.

- We acknowledge the need for multiple corrections in some of the comparisons and have introduced the corrected values in the Supplementary Tables 9-12. However, we do investigate the bias of correlations and find it to be very significant – variables of disease severity mostly correlate positively with species/modules enriched in PD and negatively with those depleted in PD, while the opposite is true for variables of health; we now report the significance of the correlation bias in the manuscript. This bias would not be observed if the correlations we describe were due to chance.

  **Action taken:**
  The values corrected for multiple testing are reported in Supplementary Tables 9-12.

11. Please include a discussion of the study limitations.

- We thank the reviewer for their comment. We have added a section on Limitations in our manuscript.

  **Action taken:**
  The text now reads (lines 482-489):

  *The present study has some limitations. First, this is a cross-sectional study, and longitudinal studies are needed to follow-up the potential conversion to PD in some individuals. Second, the relatively small number of GBA-NMC individuals might have undermined the results. Third, the lack of independent cohorts with adequate phenotyping of disease-free individuals limited the full validation of our results, even if we could show inter- and intra-group microbiome alterations similar to the ones we report in three independent cohorts with wide geographic distribution (USA, Turkey and Korea) and the association of the alterations with clinical phenotypes in PD patients in the one with clinical data available.*

12. One of the main conclusions of the study is that better nutrition rescues the microbiome; while the data are encouraging, the trends do not seem strong enough to reach this conclusion. For PD, the microbiome correlates with disease duration - those in later PD stages have difficulty eating, which likely contributes to the observed trends. Otherwise, there are many lifestyle factors that may be acting as lurking variables - for example, the healthy controls may be more likely to have a PD-like diet since many of them live with one of the PD participants. Please temper the conclusions related to nutrition in order to reflect the uncertainty.

- We thank the reviewer for this helpful insight. It is correct that advanced PD may influence dietary choice through difficulties in swallowing. However, only 2 of our PD participants were sufficiently advanced to have this problem. In PD, several studies suggest that dietary interventions such as Mediterranean diet may help reduce constipation and improve cognitive function (doi: 10.3390/nu14235185; doi:

10.1016/j.cdnut.2024.102216; doi: 10.1016/j.ctim.2020.102366; doi: 10.1080/1028415X.2020.1751509). Moreover, epidemiological studies suggest that adherence to the Mediterranean diet reduces the risk of PD (doi: 10.1016/j.jnha.2024.100451). Our data seem to align with these findings, although we acknowledge that only longitudinal studies and/or interventional studies are needed to fully support this. We have thus tempered our conclusions to focus on the potential role of the gut microbiome as a marker of early disease detection rather than interventions based on current knowledge.

**Actions taken:**
We removed the below sentence from the Discussion section:
*In such individuals, modification of factors such as diet, which is associated with microbiome alterations in our study, could help reduce the risk for PD.*

Reviewer #2 (Remarks on code availability):

The above link is invalid, and there is no code availability statement in their manuscript. I am unable to review their code.

- We apologise for this inconvenience. The link to the repository has now been made public to provide the reviewers with access to the code (lines 1166-1167).

  It is a requirement that data are appropriately anonymized and kept confidential. We have worked closely with our Funding agency (ASAP) to provide secure access to reviewers and guarantee reviewers' anonymity during the process.

  The metagenomic data is available through the ASAP CRN Cloud. Instructions for Journal Review Access through the CRN Cloud have been submitted as a separate document in the Supplementary Material ("CRN Cloud - Journal Review Access"). We specify that to apply for access, when reviewers navigate the CRN Cloud, they can select any collection card (reviewers do not need to search for any specific card related to the present study). In the Intended Use Statement section of the Access Application, reviewers can provide the following information:
    - ASAP Team Name = Schapira
    - Principal Investigator = Schapira

  As mentioned in the Instructions, details of reviewers will not be provided to the submitting researchers to guarantee reviewers' anonymity. The CRN Cloud team is available to assist reviewers at any stage of the process and can be contacted at cloud@parkinsonsroadmap.org.

  Clinical data have been deposited and will be released through GP2 (Michael J. Fox Foundation). Access requests for GP2 data should be submitted via AMP PD: https://amp-pd.org. To facilitate timely review, we have made clinical data temporarily available for reviewers through the UCL Research Data Repository at the following link: https://figshare.com/s/1f82998108e487fd47e4. We hope this approach provides a straightforward and convenient solution for the reviewers.

Reviewer #3 (Remarks to the Author):

The manuscript by Menozzi and coworkers explores the potential influence or connection between mutations in GBA1, Parkinson's disease (PD), and the gut microbiome. In a large and adequately phenotype cohort, the study deeply profiles the stool microbiome and correlates microbial species and biosynthetic pathway (ie., functions) to PD status and disease phenotypes. Overall, this is an important and timely area of research as the microbiome has been widely implicated in PD, but whether there are specific gene-microbiome interactions remains speculative and whether the microbiome can be used as a diagnostic for predicting who may develop PD is unknown. This last point is relevant to the field and to this study, due to the long prodromal phase prior to PD which may open the possibility of interventions to slow disease progression and the fact that the authors do identify microbiome signatures across the samples that may help identify individuals at risk for developing PD beyond genetic risk.

While the study finds several interesting outcomes, overall the lack of a GBA specific microbiome profile and lack of validation for many of the claims in the work limit enthusiasm. Firstly, the goal of the work, presumably, was to determine if individuals with GBA mutations show stereotypical changes in the gut microbiome—the study finds no or minor differences in the microbiome of individuals with PD regardless of GBA status. Secondly, many of the most striking differences in taxa and in some functions that distinguish PD from non-PD (HC or GBA-NMC) have been previously reported in numerous prior studies. Therefore, the proposal that the gut microbiome may explain the incomplete penetrance of GBA mutations to PD manifestation are not supported by the data generated.

- We thank the reviewer for these comments. The primary goal of our work was not to determine whether individuals with *GBA1* variants showed specific alterations in the gut microbiome *per se* (i.e., alterations driven by the host *GBA1* genotype). We addressed this question in our past work on animal models. In this previous work, we compared the gut microbiome of mice carrying a heterozygous variant in the *GBA1* gene and non-mutated mice over time, and we did not identify substantial differences, suggesting that the host *GBA1* genotype does not influence the gut microbiome composition (https://www.frontiersin.org/journals/neuroscience/articles/10.3389/fnins.2025.1546203/full). In the present work in humans, we thus wanted to test the hypothesis that beyond the genetic risk associated with carrying *GBA1* variants, alterations in the composition of the gut microbiome could better stratify individuals for their risk of PD and identify, among the GBA-NMC, those at highest risk of conversion. We know that only a minority of individuals with *GBA1* variants will develop PD over their lifetime, suggesting that additional factors, beyond *GBA1* genotype, play a role in determining the penetrance for PD. Our findings support the notion that changes in the microbiome similar to those observed in overt PD, occur in some GBA-NMC, predominantly in those with more pronounced prodromal clinical features of PD. Our data do not answer the question as to whether these GBA-NMC individuals will be the ones developing PD in the future but represent the foundation for the design of future studies to address this question. Moreover, since we identify similar changes in a proportion of healthy controls also showing more pronounced prodromal features, we suggest that gut microbiome alterations could be early signs of neurodegeneration affecting the peripheral autonomic nervous system, which would be expected to occur in body-first Lewy body diseases. Regarding the lack of validation, we provide an answer in the next response.

It is interesting that there is a gradient of microbiome effects from HC to GBA-NMC to PD, which may be one of the key findings of this study. However, the assertion that this observation may delineate a "prodromal PD microbiome" is unlikely since the actual at-risk population in the GBA-NMC is not the entire group but some subset of this stratification. While some attempts are made to find people who may indeed have prodromal PD in the GBA-NMC group, the small number (n=4) makes this comparison unsubstantiated. A discovery-validation study design is needed to determine if the microbiome can in fact identify people who may go on the develop PD, and the specificity of this prediction needs to be strong (and validated by other studies) before it may serve as a clinical biomarker.

- We thank the reviewer for raising the point of the prodromal microbiome. The term prodrome refers to an early set of symptoms presenting before the motor signs of Parkinson's disease develop. In our manuscript, we suggest that changes of the microbiome composition in disease-free individuals that resemble those found in PD individuals may be prodromal. Conceptually, they resemble prodromal symptoms such as constipation, RBD or olfaction loss, in contrast to motor symptoms more typical of overt disease. The microbiome changes we report correlate with many of these symptoms, which is the basis of our suggestion. They even identify in our study population the fraction of GBA-NMC which are considered "prodromal" by accepted clinical criteria. Admittedly the numbers are low, but the likelihood that our observations are due to chance are extremely small; the values we report are p=4.7exp-3 for the prodromal individuals to be above the median of altered microbiome distribution and 5.7exp-4 for the four being at the very top (lines 272-275). Nevertheless, we fully agree that validation across other cohorts is needed before these observations may be translated into clinically useful biomarkers. Therefore, we examined three independent cohorts of patients and healthy controls, from USA, Korea and Turkey. In all cases, we found microbiome alterations like the ones we report in our cohort from Europe (UK and Italy), both in PD patients and within each of the groups (see the new section "Validation of microbiome alterations across independent cohorts" – lines 404-448), indicating that they are consistent across these global communities. Due to the lack of adequate phenotyping of sufficient number of healthy individuals in these and any other studies, we could not examine the association of microbiome alterations and clinical variables as performed in our paper and indicate this among the limitations of the study.

  **Action taken:**
  A new section "Validation of microbiome alterations across independent cohorts" was added to Results (lines 404-448) and a paragraph to indicate the study limitations to the Discussion section (lines 485-489), also reproduced below for convenience.

  *Third, the lack of independent cohorts with adequate phenotyping of disease-free individuals limited the full validation of our results, even if we could show inter- and intra-group microbiome alterations similar to the ones we report in three independent cohorts with wide geographic distribution (USA, Turkey and Korea) and the association of the alterations with clinical phenotypes in PD patients in the one with clinical data available.*

Finally, the authors spend significant effort to correlate microbiome features with symptoms and PD severity, where they do make some interesting observations of microbial species in HC and GBA-NMC that may predispose to disease. However, additional work is needed to

determine if these signatures are indeed specific to PD or are more generally associated with lack of health. Many of the PD-related phenotypes mapped to microbiome alterations are general features of health, and thus the study needs to cross reference their findings with non-PD phenotypes (e.g., cognition, olfaction, poor diet are too broad to assign as PD-related). The notion that dysbiosis (whether in PD or otherwise) is a function of diet and can be improved by increased nutrition is generalizable and doesn't provide actionable insights into PD.

- We thank the reviewer for these interesting points. There is no single prodromal clinical feature specific for PD, although >85% of REM sleep behavior disorder (RBD) cases convert to Lewy body disorders. However, the combination of constipation, hyposmia, depression, autonomic dysfunction and cognitive impairment is strongly indicative of underlying neurodegenerative disease. Our hypothesis is that a particular microbiome signature predisposes to PD through intestinal inflammation, enhanced alpha-synuclein synthesis by enteroendocrine cells and transit through the vagus to the dorsal motor nucleus and onward to the substantia nigra and beyond. All components of this pathway have been modelled in our and other laboratories and support the hypothesis (reviewed in https://pmc.ncbi.nlm.nih.gov/articles/PMC8078923/). The present study provides an association between the microbiome signature and the evolution of pathology from controls to PD. That said, we acknowledge that longitudinal studies are needed to confirm this association.

  **Action taken:**
  We have added a sentence reflecting the study limitations in the Discussion (lines 482-483):

  *The present study has some limitations. First, this is a cross-sectional study, and longitudinal studies are needed to follow-up the potential conversion to PD in some individuals.*

  We agree with the reviewer that currently available data do not support the notion that increased nutrition can alter microbiome composition and thus modify PD pathogenesis. In PD, several studies suggest that dietary interventions such as Mediterranean diet may help reduce constipation and improve cognitive function (doi: 10.3390/nu14235185; doi: 10.1016/j.cdnut.2024.102216; doi: 10.1016/j.ctim.2020.102366 ; doi: 10.1080/1028415X.2020.1751509), and there is epidemiological evidence suggesting that adherence to the Mediterranean diet reduces the risk of PD (doi: 10.1016/j.jnha.2024.100451). Our data seem to align with these findings, although only longitudinal studies and/or interventional studies are needed to fully support this. We have thus tempered our conclusions and rephrased our Discussion section.

  **Action taken:**
  We removed the sentence below from the Discussion section:
  *In such individuals, modification of factors such as diet, which is associated with microbiome alterations in our study, could help reduce the risk for PD.*

Reviewers' Comments:

Reviewer #1 (Remarks to the Author):

I gave a full review for the first submission. A major concern in the first submission was statistical significance. If not addressed properly, all other concerns are of no consequence. Unfortunately, this study fails statistical rigor. I empathize with authors on difficulty to identify and recruit GBA group, but the fact remains that study does not have sufficient sample size for the analyses presented. As a result, claims are not reliable. Starting with Clinical profile of GBA-NMC Lines 127-143: Having conducted 22 tests in table 1, none of the reported p-values are significant, hence claims in this section are unsupported. Moving next to "Significant microbiome alterations in PD. A total of 176 species were differentially abundant ($p<0.05$, Supplementary Table 1)." $P<0.05$ is unacceptable for differential abundance analysis. Supplementary Table 1 is not results of differential abundance. "Further analyses were based on the 176 species differentially abundant at $p<0.05$." Indeed, the rest of the paper is further mining/testing of results that were probably not significant to begin with. Dicing the small dataset into smaller pieces, adding test after test with numerous variables, is bound to have high false positive rate. I liked the idea. It is truly unfortunate that sample size was so small and study design did not or could not correct.

- We thank the reviewer for their comment and for their understanding regarding the difficulty in recruiting and enrolling *GBA1* variant carriers, who are matched for demographics with the PD population. We recognise that analysing clinical data and metagenomics data separately to estimate PD risk is limited, hence our hypothesis to use combined datasets (clinical and microbiome) to refine our ability to estimate PD risk at an individual level.

  Regarding the clinical data, we have followed the Reviewer's suggestion and applied the Benjamini-Hochberg procedure for multiple testing for the comparison of clinical features (19 variables for the comparison between HC vs GBA-NMC, and for the comparison between HC vs PD, as indicated in Table 1; MDS-UPDRS part IV cannot be calculated in non-PD individuals, hence this variable has not been considered, and for RBDSQ and MOCA scores, we have adjusted the p values from the binary regression models as these are more indicative than the p values from the comparison of the raw scores, as explained in the Legend of Table 1). After adjustment for multiple testing, the GBA-NMC group shows more severe motor disability and non-motor features, and overall global severity, as represented by higher scores in MDS-UPDRS part II, I and total scores respectively. We appreciate the reviewer's suggestion, and we believe that these additional analyses further strengthen the robustness of our findings. We fully acknowledge that clinical features distinguishing GBA-NMC individuals from healthy controls are not, on their own, definitive indicators of individual PD risk. Indeed, prior studies have similarly encountered challenges in identifying consistent clinical markers that reliably predict progression to PD within GBA-NMC cohorts (reviewed in https://doi.org/10.1007/s11910-023-01259-1 ). This is precisely why our work emphasises the combined evaluation of clinical and microbiome features: integrating these complementary domains provides a more refined and potentially more accurate approach to estimate individualised PD risk.

We acknowledge that the sample size, especially for GBA-NMC, is limited and our study is cross-sectional, hence the limited conclusions that we can draw on disease progression, which can only arise from assessment of longitudinal cohorts over several years. We have been transparent about both limitations, in our Limitations section (Lines 490-492). However, as Reviewer 1 pointed out in his first revision of the manuscript, in our study, we were able to leverage an exceptionally rich clinical dataset, which includes detailed prodromal phenotyping that, to our knowledge, has not been presented in any previous clinical-microbiome study. By integrating these deeply characterised clinical features with shotgun metagenomic data and analysing them using a novel and conceptually distinct approach, we provide a robust framework for identifying GBA-NMC individuals at higher prodromal PD risk. These individuals exhibit both more pronounced prodromal features and a microbiome profile more closely resembling that of patients with manifest PD. We believe that this integrative framework has strong potential for translation and could be applied to other prodromal populations in the broader context of neurodegenerative disorders.

**Actions taken:**

1) We updated the section "Clinical profile of GBA-NMC" (Lines 122-130) and Table 1 with the p and corrected p values (q values) after adjustment for multiple testing.
2) We revised our Methods section ("Statistical analysis for clinical data"; Lines 774-778).
3) We added one sentence at the end of the section "Clinical profile of GBA-NMC" to reflect the importance of integrating complementary domains beyond clinical features to estimate PD risk (Lines 136-137, also reported below):

   *Additional biological markers indicating PD proximity are needed to better refine individualised risk of PD.*

Regarding microbiome alterations, we respectfully disagree with the reviewer that raw p-values are not acceptable for differential abundance analysis, but we acknowledge that we should be more precise in our expression and therefore we have changed the text to read "A total of 176 species were differentially abundant at $p<0.05$" (Line 156). All the corrected p-values and p values are reported in the relevant Supplementary Tables, for interested readers. We have also centred the text describing comparisons of healthy controls and PD patients on corrected p-values for both taxonomy and functions, aligning with the comment of another reviewer. However, for the downstream analysis we have kept the features significant at $p<0.05$, because we complement the usual individual species by species differential abundance analysis with the innovative coherence analysis of species pools, which gives much higher statistical power and indicates very strong statistical significance. What we deploy is the exact opposite of "dicing" – we pool and thus acquire power.

**Actions taken:**

1) We changed the text at Line 156 as indicated above ("differentially abundant at $p<0.05$").
2) Differential abundance of microbial species is now centred on the q-values in the results (Lines 157-168) and Supplementary Table 2.
3) Differential abundance of modules is now centred on the q-values (Lines 369-372) and Supplementary Table 14.

Reviewer #2 (Remarks to the Author):

The authors have done a good job revising the manuscript, but there are some remaining concerns. In my original review, my primary concerns revolved around certain aspects of the statistical analysis. While I accept many of their justifications, there are a few items that will need to be addressed before the manuscript could be considered for publishing.

I previously noted that the lack of P value correction was an issue in this manuscript. While I would argue this is appropriate in certain contexts (the demographics table, the differential results that are then aggregated and assessed as groups of features), there are multiple instances in the manuscript where it is objectively incorrect:

• Results/Discussion: differential abundance of individual microbes and features are discussed at some length, but use P values instead of corrected P values.

- We thank the reviewer for their comments.

  Regarding clinical features, we now report p values and corrected p values (q values) after Benjamini-Hochberg procedure for multiple testing for the comparison between HC vs GBA-NMC, between HC vs PD (indicated in Table 1) and for the comparison between GBA-PD and idiopathic PD (indicated in Supplementary Information, Appendix A). After adjustment for multiple testing, the GBA-NMC group shows more severe motor disability and non-motor features, and overall global severity, as represented by higher scores in MDS-UPDRS part II, I and total scores respectively. The results for the other 2 comparisons also remain largely unchanged.

  Regarding microbiome features, we now present Benjamini-Hochberg corrected p-values in the text, as detailed below; the overall messages remain unchanged.

  **Actions taken:**

  1) We updated the section "Clinical profile of GBA-NMC" (Lines 122-130) and Table 1, and Supplementary Information, Appendix A, with the p and corrected p values (q values) after adjustment for multiple testing.
  2) We revised our Methods section ("Statistical analysis for clinical data"; Lines 774-778).
  3) Differential abundance of microbial species is now centred on the q-values in the results (Lines 157-168) and Supplementary Table 2.
  4) Differential abundance of modules is now centred on the q-values (Lines 369-372) and Supplementary Table 14.

• Downstream analyses: To be considered significant, variables need to pass 6/8 tests (etc). However, these tests also use P values. The issue with this is that the tests are not orthogonal – as shown in the Reviewer Response, the abundance and prevalence outcomes are relatively strongly correlated. While the correlation isn't perfect, this still means that their correlations with any other variable are still much more likely to be similar, potentially making correlations artifactually appear more robust. Using this approach is therefore not a robust substitution for P value correction, although I very much like the idea of looking at the microbiome from multiple angles.

o I recommend keeping the 6/8 tests, but using corrected P values instead of raw P values. This may be slightly more strict than choosing one metric, but this is fair given the instability of such a downstream analysis, and would lend more credence to the results given that the approach is non-standard (but interesting).

- We thank the reviewer for their comment regarding the statistics. We show now the corrected p-values (p.adj) in Supplementary Tables 5-7 and 22-23. However, we keep the “raw” p-values in Extended Table 1, as we feel that they illustrate more fully the extent of correlation between microbiome alterations and clinical variables. Indeed, all the clinical variables significant at $p<0.05$, without exception, go in the expected direction: severity variables are higher for the group with more altered microbiome, and the health variables are lower, as stated in the text (Lines 241-249). This is totally unexpected for false positives, which the FDR is supposed to eliminate – there is no reason that they would display such bias. We conclude that the variables eliminated upon FDR would be eliminated incorrectly and the message would thus be weakened wrongly.

o This statement in the Reviewer Response exemplifies the potential issue: “Using both the proportion and the abundance of species allows us to identify a higher number of clinical parameters which associate significantly with microbiome alterations.” While this approach will increase sensitivity, it loses specificity. An increase in significance suggests that the results may be becoming less robust. The researchers seem to be working under the assumption that the microbiome will correlate with clinical features (ex. lines 233-5 of the revised manuscript).

- The reviewer is quite right that we assume that the microbiome alterations will correlate with clinical features and we now explicitly state that this is our hypothesis.

- **Action taken:**
  We modified the text which now reads:
  1) Lines 230-231: “*We hypothesized that higher gut microbiome alterations were associated with worse clinical features in PD patients.*”
  2) Lines 241-242: “*The first class corresponded to variables associated with disease severity, found to be higher in individuals in the top quartile of microbiome alterations, in agreement with our hypothesis.”*
  3) Lines 247-248: *“The second class included variables associated with health, found to be worse in individuals with more altered microbiome, also in agreement with our hypothesis.”*

In line with the above, abundance and proportion should also be defined in the manuscript as the way the manuscript/response is written suggests that they may have been used differently than is typical in the field. Without a definition, it is difficult to follow the related discussions (ex. ‘Associations of microbial alterations and clinical variables in PD’).

- We thank the reviewer for the comment and now refer to the Methods section in the Main text.

  **Actions taken:**
  1) The text now reads (Lines 221-222): *“Abundance and proportion were computed as described in the Methods.”*

2) The relevant Methods section reads (Lines 887-889): *"Proportion was computed as the number of cognate species present in the sample, divided by the total number of species in the sample. Abundance was computed as total abundance of cognate species divided by abundance of all species in the sample."*

Supplementary Table 1 seems to be an abundance table instead of the differential abundance results – please update.

- This Table has both species abundances in each sample (columns B-QW) and the differential abundance result for the PD/HC comparison (columns RB-RD).

I have one very minor remaining point that does not constitute a barrier to publishing: after looking at the code, I believe I misunderstood how 'proportion' was defined in the original manuscript. Adding the definition into the manuscript would be very helpful since there are multiple ways that it can be interpreted.

- The way proportion was computed is described in the Methods (Lines 887-888): *"Proportion was computed as the number of cognate species present in the sample, divided by the total number of species in the sample.*

Reviewer #2 (Remarks on code availability):

The code looks to be very straightforward and understandable. There are plenty of comments and the code chunks are well partitioned. There are no obvious errors. I anticipate no difficulties in replicating the findings.

- We thank the reviewer for this comment.

Reviewer #3 (Remarks to the Author):

The manuscript has been satisfactorily revised and my comments addressed.

- We thank the reviewer for this comment.
